# Richness of non-timber forest products in Himalayan communities—diversity, distribution, use pattern and conservation status

**DOI:** 10.1186/s13002-020-00405-0

**Published:** 2020-09-23

**Authors:** Haseeb Ul Rashid Masoodi, R. C. Sundriyal

**Affiliations:** 1 G.B. Pant National Institute of Himalayan Environment, Kosi-Katarmal, Almora, Uttarakhand 263643 India; 2 Department of Forestry and Natural Resources, HNB Garhwal University, Srinagar, Uttarakhand 249161 India

**Keywords:** NTFPs, Livelihoods, Diversity, Distribution, Use pattern, Conservation status, Threat categorization, Western Himalaya, Himachal Pradesh

## Abstract

**Background:**

Non-timber forest products (NTFPs) are important resources for sustenance of rural communities; a systematic planning to manage diverse NTFPs may immensely contribute to food and livelihood security of forest dwellers. Considering this, the present study has been undertaken in the Himachal Pradesh state in north India. It aims to provide detailed information on diversity, distribution, use pattern, and conservation status of selected NTFPs that have market potential, and suggest a possible way for their sustained management and possible role in livelihood upgradation of dependent communities.

**Methodology:**

An inventory of NTFP species was prepared by collecting secondary information from published scientific studies in journals, books, and other periodicals as well as species being traded as per Forest department records. Search on various online databases were also used (Scopus, Google Scholar, PubMed, ISI Web of Science) using specific search terms such as “non-timber forest products,” “NTFPs,” “medicinal plants,” “wild edible plants,” and “Himachal Pradesh,” “Western Himalaya,” and “Northwest Himalaya.” A list of potential NTFPs was prepared having market value. To evaluate the relative usefulness of different species, a quantitative valuation was also used by calculating various indices, such as use value (UV), relative frequency of citation (RFC), relative importance index (RI), cultural importance index (CI), and cultural value (CV).

**Results:**

A total of 811 species have been screened that has significant potential for the State, and categorized in 18 groups as per their use. The family use value was highest for Asteraceae (FUV = 76.75). Among plant parts used, whole plants, roots (including rhizomes and tubers), leaves, flowers, fruits, seeds, stems, and barks were used by the forest dwellers. Maximum NTFPs were collected from the warm temperate zone, followed by the temperate, sub-alpine, sub-tropical, and alpine zones. Sixty-one percent of species had medicinal importance, followed by species used for food and fodder purposes. Although species richness of medicinal plants decreased with altitude, however, most plants extracted from high altitudes were high-value species fetching better income. As many as 125 NTFPs were identified under the diverse level of threats according to IUCN criteria and as per the local stakeholders’ perceptions.

**Conclusion:**

High dependence on NTFPs by poor and marginal communities for domestic needs as well as market demand of selected species leads to create excessive pressure on them. Unfortunately, the state agencies are not having any robust conservation plan for NTFPs. For long-term management of NTFPs sector, a species-specific conservation strategy, proper harvesting protocol, cultivation practices, the supply of quality planting material, product development and diversification, value chain development, and ensured market is greatly desired. This will not only lead to conserving NTFPs resources in their natural habitats but also lead a sustainable livelihood generation for forest dwellers.

## Introduction

Non-timber forest products (NTFPs) play an important role in the livelihoods of the rural poor by satisfying food, fiber, fodder, medicine, construction materials, and income needs. Nearly 60% of the world’s forests that cover approximately 2.4 billion hectares of land are primarily or partially used for the production of wood and non-wood forest products [1]. The role of NTFP is particularly important in the Himalayan region, where a large proportion of the rural population depend on forests for meeting their livelihood needs [2, 3]. A wide variety of animal and plant products are sourced as food, nutrition, fodder, fiber, medicine, condiment, dye, and various other uses for meeting household needs and/or for commercial purposes that generate substantial revenue [4, 5]. Livelihood security of rural people depends greatly on the status and condition of the natural resources [6, 7]. It has been estimated that many village communities derive as much as 10–50% of their household income from the sale of forest products [8–12]. Although, NTFPs do not guarantee a high or regular income for forest people [13]. The community attitude toward forest resources varies depending on the distance of forest, availability of resource, and access tenure [5, 14] and acts as a buffer during times of hardships [15–17]. Food and Agriculture Organization (FAO) estimates that 80% population in the developing countries relies on NTFPs for nutritional and health needs [18] and over 1.2 billion of the rural population generally depend on NTFPs that supplement their basic needs [19]. In India, more than 95% of the total medicinal plants used in preparing medicines by various industries are harvested from the wild [20].

Sustainable extraction of NTFPs is considered the best feasible strategy for forest conservation in biodiversity-rich areas [20]. The past decade has witnessed a rapid growth of interest among conservation and development organizations [15]. The growing commercial trade of natural products, in particular plant medicines and crafts, has resulted in an increase in the harvest volume from wild areas that leads overexploitation of many species [6, 19, 21–24]. Some species are considered commercially viable and may provide better livelihoods to communities residing in far-flung areas of the state [2]. Therefore, scientific documentation and information on diversity, distribution and use pattern, and economic importance of species can prove pivotal in the conservation and sustainable use of such plant resources in any given state and region. Further, quantitative on the relationship between biological and cultural diversity and the relative importance of natural resources for the local population can play an important role in the sustainable use and conservation of many NTFPs [25]. Considering this, the present study has been undertaken with a focus to provide baseline information on diversity, distribution, use pattern, and conservation status of selected NTFPs that have market potential and can support the livelihood along with enhancement of the ecological, natural, cultural, and socio-economic capital assets and values in a Himalayan state. By collecting data and information from all possible sources, it is expected that a comprehensive strategy may be developed for the conservation and sustainable uses of NTFPs in the target state. Considering that many other states and countries have similar circumstances, the information may be used to improve the NTFPs sector in such areas as well.

## Study area

**Fig. 1:**
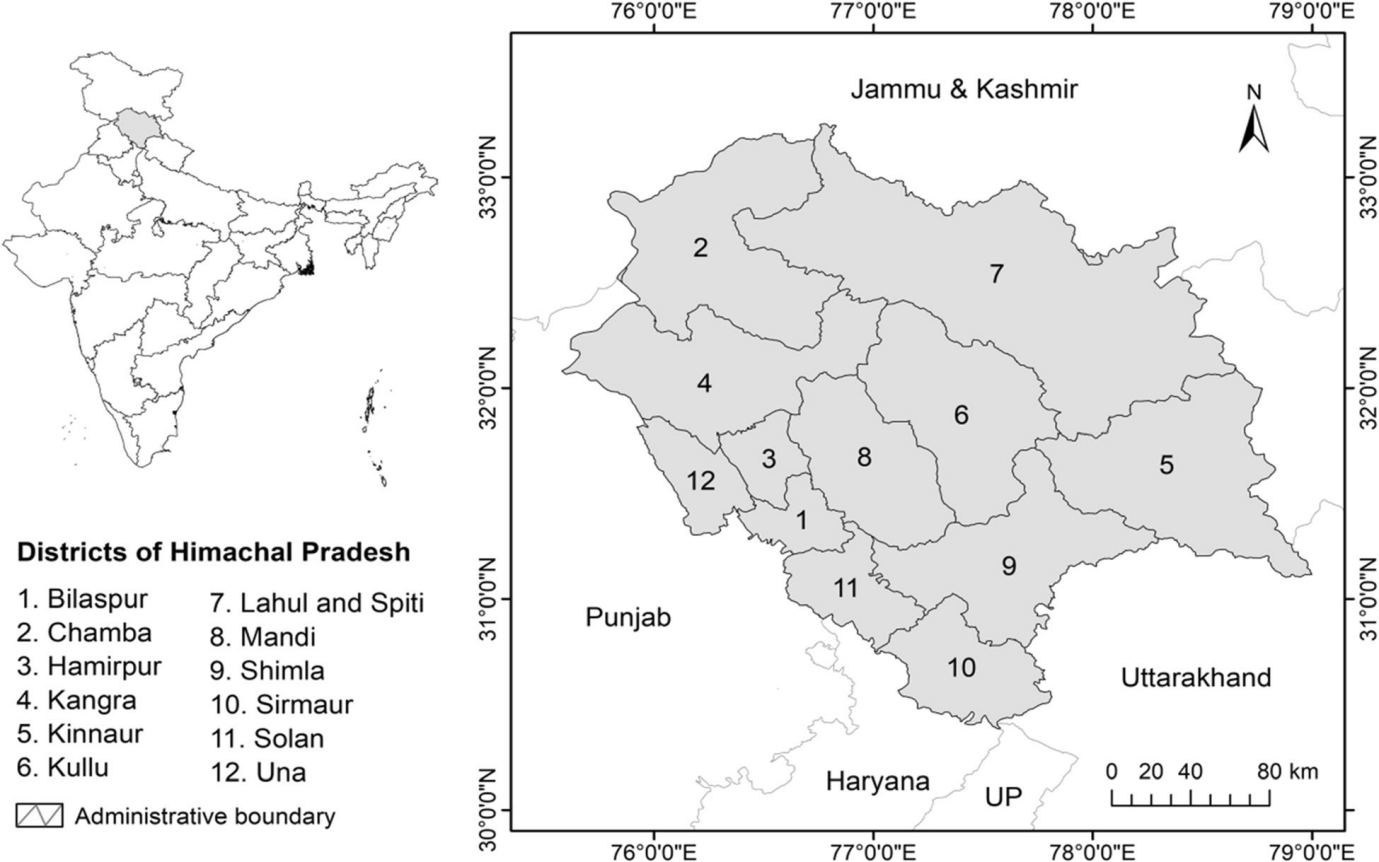
Map of the study area in Himachal Pradesh, India

The study was conducted in the state of Himachal Pradesh (30° 22′ 40″–33° 12′ 40″ N to 75° 45′ 55″–79° 04′ 20″ E) that falls in the western Himalayan region in north India. The state covers an area of 55,673 km^2^ representing 9% of the IHR. The State is bordered by Jammu and Kashmir on North, Punjab on West and South-West, Haryana on South, Uttarakhand on South-East, and China on the East (Fig. 1). It supports a hilly and mountainous terrain with all possible natural and physical features to support significant biodiversity that is used intensively by local communities for various socio-economic purposes. In view of significant dependence on NTFPs, this sector has been undertaken for investigation in the present study.

## Methodology

The study is entirely based on the literature review. A comprehensive list of NTFP species was prepared by collecting information from secondary sources, such as from published scientific studies in journals, books, periodicals, published floras, Ph.D. theses, conference proceedings, forest working plans, as well as species being traded as per Forest department records. Search on various online databases (Scopus, Google Scholar, PubMed, ISI Web of Science) was made using specific search terms such as “non-timber forest products,” “NTFPs,” “medicinal plants,” “wild edible plants,” and “Himachal Pradesh,” “Western Himalaya,” and “Northwest Himalaya.” Species with potential market value were recorded. Thus, a total of 210 articles have been separated of which 141 were taken for detailed investigation (list of references). The precision of species identification in this review was dependent on the original source. Species were verified from there currently accepted name(s) in online nomenclature sources (http://www.theplantlist.org and http://www.tropicos.org). Identifying potential species having a market value, their use patterns by communities, and trends at a regional scale. A master list was prepared to depict vernacular and botanical name(s). Data have been arranged on plant life forms, and plant parts used and sold. All inventoried NTFPs were also classified into 18 use groups, viz. medicines, edibles, resin, construction, agricultural tools, firewood, incense, spices, fodder, dyes, religious, perfumes, oil/essential oil, insecticidal, fibre, beverages, and others. The medicine category includes plants used for treating human as well as animal diseases.

## Data analysis

To further evaluate the relative usefulness of different species, a quantitative valuation was also used by calculating various indices, such as use value (UV), relative frequency of citation (RFC), relative importance index (RI), cultural importance index (CI), and cultural value (CV) as provided below.

### Use report

All the ethnobotanical indices are founded on the basic structure of the ethnobotanical information: informant “*i*” mentions the use of the species “*s*” in the use-category “*u*.” The event resulting from the combination of these three variables has been defined as a use-report [25]. For studying the cultural importance of plants, one of the most commonly used tools is the total number of use-reports (UR) for each species, i.e., fixing the variable “*s*.” This can be mathematically expressed as:

$$ {\mathrm{UR}}_S=\sum \limits_{u=u1}^{u_{\mathrm{NC}}}\sum \limits_{i=i1}^{i_N}{\mathrm{UR}}_{ui} $$

First, we sum the “UR” of all the literature sources (from “*i*_1_” to “*i*_N_”) within each use-category for that species(s); i.e., the number of literature sources who mention each use-category for the species. Second, we sum all the “UR” of each use-category (from “*u*_1_” to “*u*_NC_”).

### Use value

UV is a widely used statistic employed by ethnobotanists to provide a measure of the relative usefulness of plants to people [26, 27]. To calculate the use value of each species (i), we use the formula

$$ {\mathrm{UV}}_i=\sum {U}_i/N $$

“*U*_i_” referring to the number of categories of use mentioned for a species in a particular literature source and “*n*” the total number of literature sources mentioning the species [26–29].

### Family use value

FUV provides a measure of the relative usefulness of plant families. FUV for a particular family is calculated using the formula [27]:

$$ {\mathrm{FUV}}_i=\raisebox{1ex}{$\sum \left(\mathrm{U}{\mathrm{V}}_{\mathrm{i}}\right)$}\!\left/ \!\raisebox{-1ex}{$n$}\right. $$

where UV_i_ is the use value of species *i* and *n* is the number of species in the family.

### Relative frequency of citation

The statistic RFCs are used as a measure of consensus between the information provided by different literature sources. The RFC value describes the local importance of each recorded species. RFC for a species is calculated as

$$ {\mathrm{RFC}}_{\mathrm{s}}=\frac{{\mathrm{FC}}_{\mathrm{s}}}{N}=\frac{\sum \limits_{i={i}_1}^{i_N}{\mathrm{UR}}_i}{N} $$

where FC_s_ is the number of literature sources mentioning species *s* and *N* the total number of literature sources consulted [30].

### Relative importance index

This index takes into account only the use-categories using the following formula [30]:

$$ \mathrm{R}{\mathrm{I}}_{\mathrm{S}}=\frac{{\mathrm{RFC}}_{S\left(\max \right)}+\kern0.5em {\mathrm{RNU}}_{S\left(\max \right)}}{2} $$

where RFC_s(max)_ is the relative frequency of citation over the maximum, i.e., it is obtained by dividing FC_s_ by the maximum value in all the species of the literature sources [RFC_s(max)_ = FC_s_/max (FC)], and RNU_s(max)_ is the relative number of use-categories over the maximum, obtained dividing the number of uses of the species by the maximum value in all the species of the literature sources [RN_s(max)_ = NU_s_/max (NU)].

### Cultural importance index

The cultural importance index (CI) is defined by the following formula:

$$ {\mathrm{CI}}_s=\sum \limits_{u={u}_1}^{u_{\mathrm{NC}}}\sum \limits_{i={i}_1}^{u_{\mathrm{NC}}}\raisebox{1ex}{${\mathrm{UR}}_{ui}$}\!\left/ \!\raisebox{-1ex}{$N$}\right. $$

This index, the third factor of the previously defined CV index, also can be seen as the sum of the proportion of literature sources that mention each species use.

### Cultural value

The cultural value (or importance value) of species in a given culture and the comparative importance of species inter culturally are receiving growing attention in ethnobotanical studies [26, 31]. This index is calculated using the following formula [32]:

$$ {\mathrm{CV}}_S=\left[\frac{N\kern0.5em {U}_S}{\begin{array}{cc}N& C\end{array}}\right]\times \left[\frac{\begin{array}{cc}F& {C}_S\end{array}}{N}\right]\times \left[\sum \limits_{\mu =\mu 1}^{\mu_{\mathrm{NC}}}\sum \limits_{\iota =\iota 1}^{\iota_N}\frac{{\mathrm{UR}}_{\mu \iota}}{N}\right] $$

**Fig. 2:**
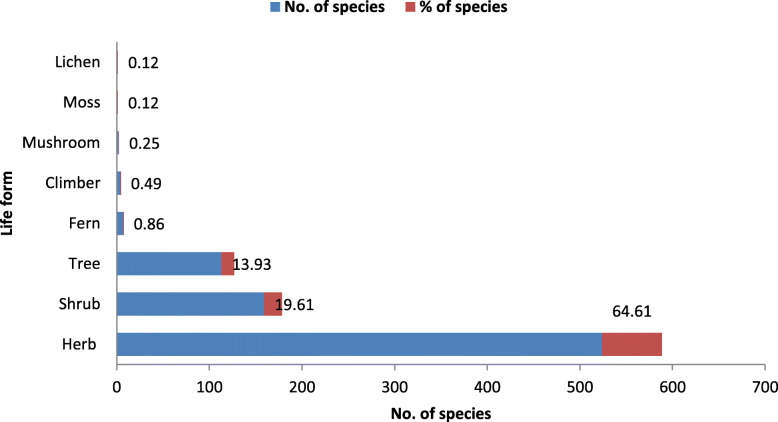
Plant habit of NTFPs in the State of Himachal Pradesh

Where the first factor is the relationship between the number of different uses reported for the species (“ethnospecies” in the original work) and the total number of use-categories considered in the study (NUs divided by NC). The second factor is the relative frequency of citation of the species (previously defined). Finally, the third factor is the sum of all the UR for the species (defined at the beginning of this section), i.e., the sum of number of literature sources who mentioned each use of the species, divided by *N*. These three factors are then multiplied together (Fig. 2).

## Results and discussion

### Diversity and distribution

**Fig. 3:**
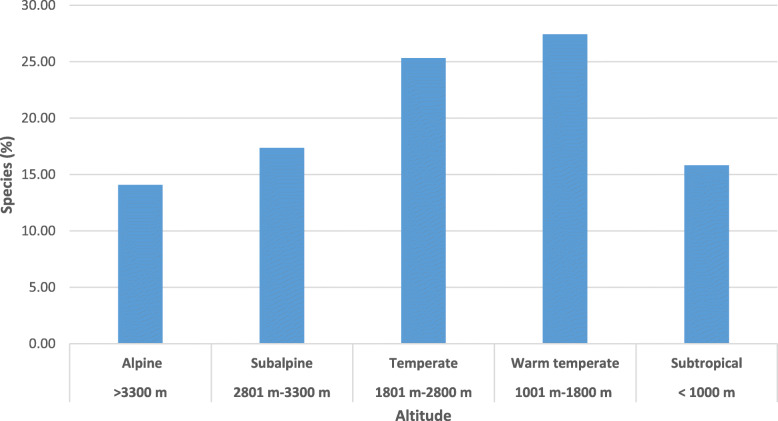
Altitudinal distribution of NTFPs in Himachal Pradesh

The State exhibits a high dependence on NTFPs for satisfying diverse domestic needs for house use and to sell it for cash needs. A total of 811 NTFPs (excluding Lichens and Moss), belonging to 128 families and 495 genera, have been screened and listed as per the name of the species, life form, use category, and additional use(s) (Additional file 1). Asteraceae, Lamiaceae, Fabaceae, Rosaceae, Ranunculaceae, Polygonaceae, Apiaceae, Poaceae, Euphorbiaceae, and Gentianaceae were top ten families that collectively encompass 44% of all species (Table 1). The herbaceous was the most dominant life form (64.6% of all species) followed by shrubs (19.6%) and trees (13.9%). Most high-value NTFPs were recorded at high altitudes, used for medicinal purposes, and dominated with herbaceous form [33, 34]. The regional patterns of species richness are a consequence of many interacting factors, such as plant productivity, competition, geographical area, regional species dynamics, regional species pool, environmental variables, and human activity [35, 36]. An analysis of NTFPs distribution pattern revealed that maximum species (27.4%) was found in warm temperate zone (1001–1800 m), followed by the temperate (1801–2800 m) (25.3%), sub-alpine (2801–3300 m) (17.4%), sub-tropical (< 1000 m) (15.81%), and alpine (> 3300 m) (14.1%) (Fig. 3). Species use has been highly dependent on the local socio-economic conditions and distribution pattern may vary from place to place [33–39]. Different families made very different contributions to different use categories. Use of NTFPs for 18 different categories show that these species are very important for the sustenance of the inhabitants. The study revealed high dependence on and a wide variety of NTFPs for the medicinal purpose (739 species), followed by edible (141 species) and fodder (109 species) purposes (Tables 2 and 3).

**Table 1:** The taxonomic composition of plants used traditionally and family use values (FUV) based on literature research

S. No	Family	No. of genus (%)	No of species (%)	Family use value (FUV)
1	Asteraceae	31 (6.26)	64 (7.91)	76.75
2	Lamiaceae	29 (5.86)	50 (6.18)	56.75
3	Fabaceae	32 (6.46)	47 (5.81)	52.58
4	Rosaceae	18 (3.64)	44 (5.44)	35.58
5	Ranunculaceae	10 (2.02)	37 (4.57)	40.17
6	Polygonaceae	7 (1.41)	29 (3.58)	31.25
7	Apiaceae	17 (3.43)	28 (3.46)	36.75
8	Poaceae	24 (4.85)	23 (2.84)	13.75
9	Euphorbiaceae	7 (1.41)	17 (2.10)	43.75
10	Gentianaceae	6 (1.21)	16 (1.98)	15.17
11	Orchidaceae	13 (2.63)	16 (1.98)	12.67
12	Liliaceae	8 (1.62)	12 (1.48)	17.50
13	Malvaceae	10 (2.02)	12 (1.48)	13.50
14	Moraceae	2 (0.40)	12 (1.48)	21.42
15	Solanaceae	8 (1.62)	12 (1.48)	29.75
16	Acanthaceae	8 (1.62)	11 (1.36)	17.25
17	Scrophulariaceae	8 (1.62)	11 (1.36)	13.50
18	Zingiberaceae	7 (1.41)	11 (1.36)	11.75
19	Boraginaceae	8 (1.62)	10 (1.24)	11.42
20	Rubiaceae	8 (1.62)	10 (1.24)	13.75
21	Rutaceae	8 (1.62)	10 (1.24)	16.00
22	Caesalpiniaceae	4 (0.81)	9 (1.11)	22.00
23	Caprifoliaceae	4 (0.81)	9 (1.11)	8.25
24	Amaranthaceae	4 (0.81)	8 (0.99)	8.25
25	Berberidaceae	2 (0.40)	8 (0.99)	10.75
26	Oleaceae	4 (0.81)	8 (0.99)	5.75
27	Urticaceae	5 (1.01)	8 (0.99)	10.75
28	Cyperaceae	3 (0.61)	7 (0.87)	6.50
29	Ericaceae	4 (0.81)	7 (0.87)	14.75
30	Araceae	4 (0.81)	6 (0.74)	8.75
31	Commelinaceae	4 (0.81)	6 (0.74)	6.50
32	Cupressaceae	2 (0.40)	6 (0.74)	11.75
33	Pinaceae	3 (0.61)	6 (0.74)	14.75
34	Plantaginaceae	2 (0.40)	6 (0.74)	5.50
35	Anacardiaceae	5 (1.01)	5 (0.62)	16.75
36	Apocynaceae	5 (1.01)	5 (0.62)	7.25
37	Convolvulaceae	3 (0.61)	5 (0.62)	14.00
38	Crassulaceae	5 (1.01)	5 (0.62)	6.25
39	Cuccurbitaceae	5 (1.01)	5 (0.62)	11.25
40	Elaeagnaceae	3 (0.61)	5 (0.62)	7.75
41	Fumariaceae	2 (0.40)	5 (0.62)	6.25
42	Hypericaceae	1 (0.20)	5 (0.62)	7.00
43	Menispermaceae	4 (0.81)	5 (0.62)	5.50
44	Pteridaceae	2 (0.40)	5 (0.62)	3.58
45	Rhamnaceae	2 (0.40)	5 (0.62)	9.00
46	Verbenaceae	5 (1.01)	5 (0.62)	13.00
47	Agavaceae	2 (0.40)	4 (0.49)	6.50
48	Asclepiadaceae	4 (0.81)	4 (0.49)	8.75
49	Betulaceae	5 (1.01)	4 (0.49)	6.50
50	Brassicaceae	4 (0.81)	4 (0.49)	4.00
51	Caryophyllaceae	4 (0.81)	4 (0.49)	4.25
52	Celastraceae	3 (0.61)	4 (0.49)	9.50
53	Geraniaceae	1 (0.20)	4 (0.49)	6.00
54	Lauraceae	4 (0.81)	4 (0.49)	4.50
55	Lythraceae	4 (0.81)	4 (0.49)	12.25
56	Meliaceae	3 (0.61)	4 (0.49)	11.00
57	Primulaceae	3 (0.61)	4 (0.49)	5.25
58	Sapindaceae	4 (0.81)	4 (0.49)	7.50
59	Saxifragaceae	3 (0.61)	4 (0.49)	5.75
60	Teliaceae	2 (0.40)	4 (0.49)	4.75
61	Violaceae	1 (0.20)	4 (0.49)	7.75
62	Vitaceae	3 (0.61)	4 (0.49)	3.50
63	Asparagaceae	1 (0.20)	3 (0.37)	13.50
64	Balsaminaceae	1 (0.20)	3 (0.37)	3.67
65	Combretaceae	1 (0.20)	3 (0.37)	12.50
66	Fagaceae	1 (0.20)	3 (0.37)	6.00
67	Iridaceae	1 (0.20)	3 (0.37)	2.00
68	Linaceae	3 (0.61)	3 (0.37)	2.50
69	Onagraceae	3 (0.61)	3 (0.37)	2.75
70	Oxalidaceae	1 (0.20)	3 (0.37)	7.50
71	Saurauiaceae	3 (0.61)	3 (0.37)	4.75
72	Ulmaceae	2 (0.40)	3 (0.37)	3.00
73	Alliaceae	1 (0.20)	2 (0.25)	1.00
74	Araliaceae	2 (0.40)	2 (0.25)	2.00
75	Arecaceae	2 (0.40)	2 (0.25)	2.25
76	Athyriaceae	1 (0.20)	2 (0.25)	1.00
77	Bignoniaceae	2 (0.40)	2 (0.25)	2.25
78	Buddlejaceae	1 (0.20)	2 (0.25)	1.00
79	Buxaceae	2 (0.40)	2 (0.25)	1.75
80	Campanulaceae	1 (0.20)	2 (0.25)	1.42
81	Dioscoreaceae	1 (0.20)	2 (0.25)	4.00
82	Ephedraceae	2 (0.40)	2 (0.25)	2.00
83	Flacourtiaceae	2 (0.40)	2 (0.25)	1.75
84	Hypoxidaceae	2 (0.40)	2 (0.25)	1.00
85	Myrsinaceae	2 (0.40)	2 (0.25)	2.25
86	Myrtaceae	2 (0.40)	2 (0.25)	4.75
87	Nyctaginaceae	2 (0.40)	2 (0.25)	8.75
88	Papaveraceae	2 (0.40)	2 (0.25)	2.25
89	Parnassiaceae	1 (0.20)	2 (0.25)	1.00
90	Simaroubaceae	2 (0.40)	2 (0.25)	2.00
91	Sterculiaceae	2 (0.40)	2 (0.25)	1.75
92	Symplocaceae	2 (0.40)	2 (0.25)	2.50
93	Thymeleaceae	2 (0.40)	2 (0.25)	2.00
94	Valerianaceae	1 (0.20)	2 (0.25)	6.67
95	Zygophyllaceae	2 (0.40)	2 (0.25)	1.00
96	Achyranthaceae	1 (0.20)	1 (0.12)	5.50
97	Adoxaceae	1 (0.20)	1 (0.12)	0.50
98	Agaricaceae	1 (0.20)	1 (0.12)	0.75
99	Balanophoraceae	1 (0.20)	1 (0.12)	1.00
100	Begoniaceae	1 (0.20)	1 (0.12)	1.00
101	Cactaceae	1 (0.20)	1 (0.12)	1.00
102	Cannabinaceae	1 (0.20)	1 (0.12)	5.50
103	Capparaceae	1 (0.20)	1 (0.12)	3.00
104	Chenopodiaceae	1 (0.20)	1 (0.12)	1.00
105	Coriariaceae	1 (0.20)	1 (0.12)	1.25
106	Cuscutaceae	1 (0.20)	1 (0.12)	2.50
107	Datiscaceae	1 (0.20)	1 (0.12)	1.25
108	Gesneriaceae	1 (0.20)	1 (0.12)	0.25
109	Juglandaceae	1 (0.20)	1 (0.12)	3.75
110	Martyniaceae	1 (0.20)	1 (0.12)	0.50
111	Melanthiaceae	1 (0.20)	1 (0.12)	0.50
112	Melastomataceae	1 (0.20)	1 (0.12)	2.00
113	Morchellaceae	1 (0.20)	1 (0.12)	0.50
114	Myricaceae	1 (0.20)	1 (0.12)	3.25
115	Pedaliaceae	1 (0.20)	1 (0.12)	3.00
116	Phyllanthaceae	1 (0.20)	1 (0.12)	3.75
117	Phytolaccaceae	1 (0.20)	1 (0.12)	2.00
118	Pittosporaceae	1 (0.20)	1 (0.12)	1.00
119	Plumbaginaceae	1 (0.20)	1 (0.12)	0.75
120	Podophyllaceae	1 (0.20)	1 (0.12)	5.50
121	Portulacaceae	1 (0.20)	1 (0.12)	0.33
122	Punicaceae	1 (0.20)	1 (0.12)	3.00
123	Salicaceae	1 (0.20)	1 (0.12)	1.50
124	Santalaceae	1 (0.20)	1 (0.12)	0.50
125	Smilacaceae	1 (0.20)	1 (0.12)	1.75
126	Taxaceae	1 (0.20)	1 (0.12)	1.50
127	Trillidiaceae	1 (0.20)	1 (0.12)	0.50
128	Woodsiaceae	1 (0.20)	1 (0.12)	0.50

**Table 2:** NTFPs use categories identified and corresponding numbers of species from Himachal Pradesh

	Species use category	No. of species	Percentage of the total (%)
1	Medicinal	739	61.43
2	Edible (vegetable, fruit)	141	11.72
3	Fodder	109	9.06
4	Fuel	45	3.74
5	Other	28	2.33
6	Oil/essential oil	27	2.24
7	Construction	19	1.58
8	Dyes	16	1.33
9	Agricultural tools	15	1.25
10	Religious	15	1.25
11	Spices	12	1.00
12	Incense	10	0.83
13	Insecticidal	10	0.83
14	Resin	8	0.67
15	Fibre	3	0.25
16	Perfumes	2	0.17
17	Beverages	2	0.17
18	Mushroom	2	0.17

**Table 3:** Contributions of the top 10 family (in term of numbers of species) to different use categories

S. No	Family	ME (%)	EO (%)	ED	Res (%)	CO	AT	FW	IN	SP	FO	DY	RE	PE	IS	FI	BO	OT
1	Asteraceae	62 (8.39)	3 (11.11)	2 (1.98)	0 (0.00)	0 (0.00)	0 (0.00)	0 (0.00)	1 (10.00)	0 (0.00)	4 (3.67)	2 (12.50)	0 (0.00)	0 (0.00)	2 (20.00)	0 (0.00)	0 (0.00)	0 (0.00)
2	Lamiaceae	47 (6.36)	5 (18.52)	5 (4.95)	0 (0.00)	0 (0.00)	0 (0.00)	0 (0.00)	0 (0.00)	2 (16.67)	1 (0.92)	1 (6.25)	1 (6.67)	0 (0.00)	0 (0.00)	0 (0.00)	0 (0.00)	1 (3.57)
3	Fabaceae	40 (5.41)	0 (0.00)	1 (0.99)	0 (0.00)	1 (5.26)	3 (20.00)	11 (24.44)	0 (0.00)	0 (0.00)	15 (13.76)	0 (0.00)	1 (6.67)	0 (0.00)	0 (0.00)	0 (0.00)	0 (0.00)	1 (3.57)
4	Rosaceae	37 (5.01)	1 (3.70)	26 (25.74)	0 (0.00)	2 (10.53)	2 (13.33)	1 (2.22)	1 (10.00)	0 (0.00)	7 (6.42	0 (0.00)	1 (6.67)	0 (0.00)	0 (0.00)	0 (0.00)	1 (50.00)	5 (17.86)
5	Ranunculaceae	35 (4.92)	0 (0.00)	0 (0.00)	0 (0.00)	0 (0.00)	0 (0.00)	0 (0.00)	0 (0.00)	0 (0.00)	3 (2.75)	0 (0.00)	0 (0.00)	0 (0.00)	0 (0.00)	0 (0.00)	0 (0.00)	1 (3.57)
6	Polygonaceae	28 (3.79)	0 (0.00)	7 (6.93)	0 (0.00)	0 (0.00)	0 (0.00)	0 (0.00)	0 (0.00)	0 (0.00)	1 (0.92)	0 (0.00)	0 (0.00)	0 (0.00)	0 (0.00)	0 (0.00)	0 (0.00)	0 (0.00)
7	Apiaceae	25 (3.38)	4 (14.81)	2 (1.98)	0 (0.00)	0 (0.00)	0 (0.00)	0 (0.00)	3 (30.00)	2 (16.67)	3 (2.75)	0 (0.00)	0 (0.00)	2 (100.00	2 (20.00)	0 (0.00)	0 (0.00)	0 (0.00)
8	Gentianaceae	16 (2.17)	0 (0.00)	0 (0.00)	0 (0.00)	0 (0.00)	0 (0.00)	0 (0.00)	0 (0.00)	0 (0.00)	0 (0.00)	0 (0.00)	0 (0.00)	0 (0.00)	0 (0.00)	0 (0.00)	0 (0.00)	0 (0.00)
9	Euphorbiaceae	14 (1.89)	0 (0.00)	2 (1.98)	0 (0.00)	0 (0.00)	0 (0.00)	1 (2.22)	0 (0.00)	0 (0.00)	3 (2.75)	1 (6.25)	1 (6.67)	0 (0.00)	0 (0.00)	0 (0.00)	0 (0.00)	0 (0.00)
10	Poaceae	14 (1.89)	0 (0.00)	2 (1.98)	0 (0.00)	0 (0.00)	0 (0.00)	0 (0.00)	0 (0.00)	0 (0.00)	11 (10.09)	0 (0.00)	1 (6.67)	0 (0.00)	0 (0.00)	0 (0.00)	0 (0.00)	0 (0.00)
	Total	316 (44.38)	14 (51.85)	47 (46.53)	0 (0.00)	3 (15.79)	5 (33.33)	13 (28.89)	5 (50.00)	4 (33.33)	49 (44.95)	4 (25.00)	5 (33.33)	2 (100.00	4 (40.00)	0 (0.00)	1 (50.00)	8 (28.57)

*ME* medicine, *EO* oil/essential oil, *ED* edible, *Res* resin, *CO* construction, *AT* agricultural tools, *FW* fuel wood, *IN* incense, *SP* spices, *FO* fodder, *DY* dyes, *RE* religious, *PE* perfumes, *IS* insecticidal, *FI* fiber, *OT* other

### Collection procedure

The NTFPs collectors are generally local right-holders who are allowed to collect species from the forests by paying a collection fee and issuing a permit by the Forest department. Although, people among themselves by mutual consensus have further sub-divided the forests and common lands. All NTFPs collected from the government-managed forest and traded from the district of origin are subject to a royalty payment. In practice, taxation and issuing the permits is the only government policy for NTFP management. However, such a mechanism does not encourage sustainable harvesting of NTFPs. The main high-value products are herbs collected from vast areas of government-owned lands, such as from alpine pasture during summer months. After collection and harvesting from wild areas, NTFPs pass through a series of middlemen who gather the material from different parts into large volumes for trade. In some instances, women also join the menfolk on their trekking to the alpine pastures for the collection of the herbs. As NTFPs-derived cash income is the principal cash earner among the poorer households, NTFPs do not, however, serve as mere gap fillers or just a safety net; they are a cornerstone in household livelihood strategies as has been found elsewhere [40–42]. The role of NTFPs is particularly important in the Himalayan region where a large proportion of the rural population depends on them as a source of wild fruits, vegetables, fodder, medicinal plants, food, fibre, dye, and other useful materials for daily needs and trade [37].

### NTFPs use pattern

Many plants and plant products taken from forests are used as food for humans and animals. These include whole plants, leaves, roots, fruits, nuts, etc. Different families made very different contributions to different use categories. Maximum species were used for the medicinal purpose (61.43%), followed by edibles (11.72%), whereas less than 10% species used for fodder, fuelwood, oil/essential oil, construction, dyes, agricultural tools, spices, incense, insecticidal, resin, perfumes, beverages, fiber, mushroom, and religious purposes. The major wild edible products were vegetables, mushrooms, root tubers, nuts, seeds, etc. (Table 2). Indian Himalayan Region (IHR) is the rich repository of medicinal plants [43, 44]. Local people in the Himalayan region use a wide range of wild and non-cultivated edible plants for food, spice, and cultural purposes [45]. Many plants have commercial importance, particularly for medicinal and aromatic purposes, and are traded in large quantities to earn wages and cash [46–48]. In the Himalayan region, consumption of wild species as food has been reported high and round the year, more during the lean period [5]. Wild edible plants are crucial not only for their role as a source of food and nutrition but are also an integral part of the culture and traditions of the Himalayan societies [2, 49, 50]. Locally available and commercially valuable natural resources have the potential to improve the livelihoods of rural mountain people [51, 52]. Such diversity supports to health care and nutrition and can significantly contribute to rural wellbeing through proper planning [37, 53, 54].

**Fig. 4:**
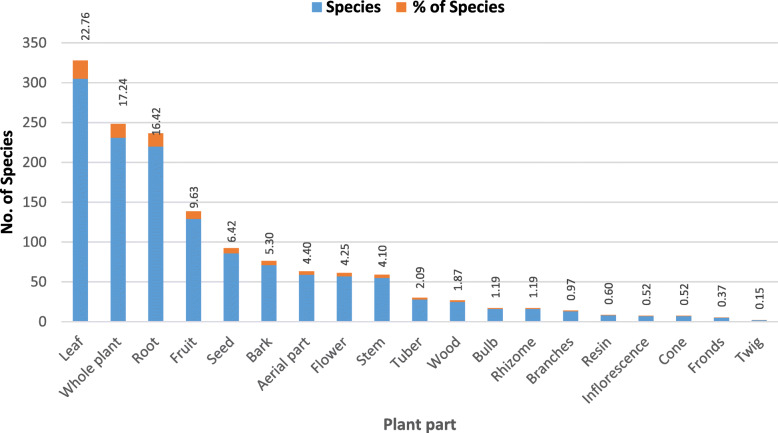
Diversity of plant part used in Himachal Pradesh

The top ten families contributed 44.3% of all species used for medicinal purposes and 47% as edible species. In the case of fodder, almost all the families contributed (45%) except Gentianaceae. Three of the top ten families contributed 50.00% of species used as incense. Asteraceae, Lamiaceae, Rosaceae, and Apiaceae together contributed 51.8% of species used for oil purposes. Fabaceae, Rosaceae, and Euphorbiaceae greatly contributed to fuelwood (Table 3). The use of NTFPs is great characteristics by people’s knowledge regarding plant habitats, time of availability, and plant parts used [55]. Forest dwellers collect edible wild plants very frequently. Selected species were commercially exploited and sold to middlemen as their market was outside the state. Various plant parts were used to prepare different medicinal formulations. The most frequently used plant parts were leaves (22.8%) and whole plant (17%) (Fig. 4). Roots and rhizomes were also used very frequently in the preparation of traditional remedies in view of high concentrations of bioactive compounds [56–61].

Local communities still rely on medicinal plants for primary healthcare and treating common ailments. A most common use was for treating skin diseases, cuts and wounds (150 species each), cough and cold (143 species), stomach infection, and rheumatism (94 species). It was also recorded that a single species may be used for curing many ailments. Though, the majority of the plants are available in the vicinity of forests, however, select people travel to far off areas such as alpine regions to collect species like *Astragalus candoleanus*, *Eritrichium canum*, *Aconitum heterophyllu*, *Polygonum viviparum*, *Picrorhiza kurroa*, *Fritillaria roylei*, *Nepeta govaniana*, *Gentianopsis detonsa*, *Saussurea gossypiphora*, *Saussurea graminifolia*, *Cortia depressa*, *Physochlaina praealta*, and *Nardostachys jatamansi*.

Forest dwellers were aware of collection seasons, mode of collection, and frequency of collection of specific parts of plant species. Gastrointestinal disorders; fever; cold, cough, and sore throat; musculoskeletal disorders; dermatological infections; respiratory system disorders; and nutritional disorders were treated with the highest diversity of medicinal plant species. Traditional knowledge is always related to local people’s contact with their resources and surroundings [28].

NTFPs did not figure high on the agenda of various forest policies till recent past; therefore, least importance was accorded to the sustainability of this important forest wealth. Unscientific extraction of these resources was most common by the collectors/contractors. However, of late, government agencies are becoming more aware of the pressures on NTFPs and some restrictions on the collection patterns have been placed. There were a large number of stakeholders and institutions involved in the trade; therefore, involving all stakeholders in decision-making and formulating the proper strategy for sustainable development of NTFPs sector is highly desirable.

### Quantitative indices of NTFPs

Various quantitative indices were applied to analyze the collected ethnomedicinal data that include informant consensus use value (UV), relative frequency citation (RFC), cultural importance index (CI), cultural value (CV), and relative importance index (RI). The UV of species varied from 0.10 to 5.20 and the highest use value was reported for the plant species which had multiple uses (Additional file 1). *Ricinus communis* (UV = 5.20), *Withania somnifera* (UV = 4.4), *Woodfordia fruticosa* (UV = 3.30), and *Terminalia chebula* and *Asparagus racemosus* (UV = 3.10) recorded maximum UV probably because of its high demand in the market. The overall plant use was proportionate to the number of plant species used in ethnomedicine. UV is one of the most frequently used indexes for evaluating “the relative usefulness of plants” [26, 28]. UV reflects both the number of uses made as well as the number of literature sources mentioning it. So the NTFPs with high UV value does not necessarily mean that it has multiple uses nor that it is necessarily mentioned in many publications [55, 62]. RFC varied between 0.30 (522 species, nearly 64.36% of the total) to 1.50 (8 species, nearly 0.99% of the total) (Table 4). Many species with high RFC scores were likely to be used over extensive geographical areas, while many of those scoring just 0.30 were likely to be used only locally. Scores for family use value (FUV) fell between 0.25 (Gesneriaceae) and 76.75 (Asteraceae). There was very little correlation between FUV and the number of species used per family (Table 1).

**Table 4:** Medicinal use of plant species in target area

Ailments treated	Species no.
Cuts and wounds	150
Skin diseases	150
Cough and cold	143
Fever	102
Dysentery and diarrhea	100
Stomach infection	94
Rheumatism	94
Tonic	83
Snake-bite	81
Urinary disorder	76
Asthma	75
Rheumatism	63
Headache	52
Sores	48
Bronchitis	45
Gastric complaints	43
Boils	43
Eye complaints	43
Stomachache	38
Toothache	37

CI ranged from 0.01 (26 species) to 1.40 (Additional file 1). The cultural value (or importance value) of species in a given culture and the comparative importance of species interculturally are receiving growing attention in ethnobotanical studies, especially those concerned with medicinal plants [26, 31]. Based on the analysis of data, the CV ranged from 0.0002 to 0.1167*.* The lowest value was for *Ficus nemoralis* while the highest cultural value was for *Verbascum thapsus* (Additional file 1).

Species with high cultural value generally have more than one uses. The greater the number of uses for the NTFP species, the more possible it is to have high cultural significance for a community. Values for particular NTFPs differ from location to location, because they relate to the evaluation by the local community of the quality, intensity, and exclusivity of the plant species within the community. A species may have high usability in one location, but not so for people in other locations [63]. The high cultural significance of a plant will stimulate the public to carry out plantings in order to obtain benefits in the near future [64]. RI varied from 0.33 (106 species) to (1.75) *Juglans regia* and *Zanthoxylum armatum* followed by *Cedrus deodara* (1.67) and *Rubus ellipticus* (1.58). High values of quantitative indexes suggest that families rich in NTFPs are more likely to be used than others, the key factor being the local presence of NTFPs potentially available for people’s attention and possible use. This result is similar to those reported for other regions [28]. The use of such indexes can make it possible to compare results between different regions or cultural groups, as well as undertaking meta-analyses [55].

### Conservation status

**Fig. 5:**
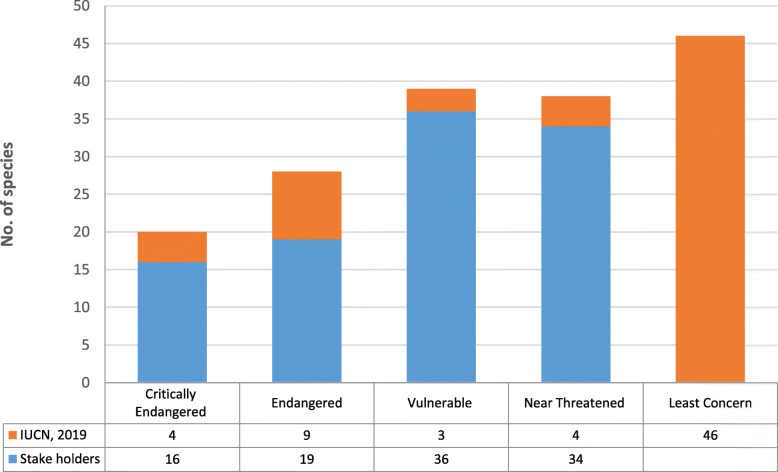
Number of threatened species mentioned by IUCN, 2019 and different stake holders

Unsustainable harvesting of NTFPs, mostly medicinal and edible plants, is the major threat to the conservation and management of NTFPs in Himachal Pradesh. Listed NTFPs of the state reveals that 125 medicinal plant species are facing various categories of a threat as per IUCN criteria and local perception. At least four species are identified as “critically endangered,” another four as “near threatened,” nine species as “endangered,” 3 species as “vulnerable,” and 46 species as least concern. According to various stakeholders, as many as 105 species are considered threatened (Fig. 5). The most common threatened species are *Aconitum heterophyllum*, *Angelica glauca*, *Gentiana kurroo*, *Nardostachys jatamansi*, *Saussurea costus*, *Lilium polyphyllum*, and *Thalictrum foliolosum*. The major concern is over-harvesting due to trade pressure. In addition, habitat destruction, livestock grazing, forest fires, etc. are also responsible for the depletion of many species. Conservation and cultivation of Himalayan medicinal plants is a key priority in the Indian Himalayan region [9, 65–67]. The high economic potential of NTFPs thus resulted in the “conservation by commercialization” hypothesis [68]. Conservation and management of NTFPs are challenged by various factors. A major lacuna is lack of appropriate policies and regulations for sustainable collection, use, trade, and management of NTFPs [46, 69]. Wild populations of many species have recently declined due to continued habitat destruction and over-exploitation [70].. Depending on the plant part harvested, lack of management may also result in overexploitation, diminished vigor of populations, and economic exhaustion of the resource [71, 72]. Many significant NTFPs are on the verge of becoming endangered due to the large quantum of collection from the wild, non-availability of any baseline data about their harvesting potential, non-availability of any field identification guide, and lack of trained staff. In the opinion of the gatherers, traders, and the forest field staff, there has been a decline in the quantum of the harvest of some species and subjective ocular assessment of their population status. A temporary ban on the collection of some species by the state government is the usual response to this situation. There has been no focused action initiated in the state to strengthen the populations of any of the species already assessed as threatened, which is a matter of great concern. In view of the increasing global demand for herbal products, the pressure on the wild populations of NTFP species, including the threatened ones, is likely to increase further which demands immediate attention.

### Implications for management

The majority of forest resource-based policies in the country have a focus on sustainable timber exploitation, thus dictated by the economic considerations for revenue generation. NTFPs have not been adequately considered in the forest management and planning process and are exposed to various challenges including the economic priorities, political will, inadequate information on the potential role of NTFPs, the irregular trade, and inadequate information on NTFPs. This has impacted negatively on the promotion and development of NTFPs in the State. An appropriate policy framework for the sustainable promotion of NTFPs is necessary to help to ensure effective development, promotion, and sustainable harvesting of NTFPs in the country. Such a strategy will also encourage the right holders to domesticate select species on their farms to reduce pressure on the forest resources. The occurrence of near-endemic, endemic, critically endangered, endangered, vulnerable, and near threatened species indicates high anthropogenic pressure on them as most of these species are commercially viable. The knowledge of diversity, its consumption pattern, contribution to rural income, and forest revenue may enable planners to accurately plan sustainable management of NTFP resources and community development in the near future. The authentic taxonomic inventory of listed NTFPs and their use can provide information for sustainable utilization of such plant wealth, which can play a pivotal role in regional sustainable development. The appropriate policy framework for conservation and development of NTFPs in the region should comprise sustainable use, conservation of gene-pool in wild areas, development of harvesting protocol, domestication of species on high demand, development of agronomic practices, availability of quality planting material, value chain development, product diversification, and value-added products with ensured market with active community participation and benefit-sharing mechanism for the local community. It would not only lead to conserving NTFPs resources in their natural habitats but also lead a sustainable livelihood generation for forest dwellers.

## Supplementary information

**Additional file 1.** Ethnobotanical inventory and some quantitative indexes of NTFPs in Himachal Pradesh.

**Additional file 2.** List of Source of literature (references) used in this study for synthesizing information.

## Data Availability

All data generated or analyzed during this study are included in this published article.
