# Supplementary material for: Richness of non-timber forest products in Himalayan communities—diversity, distribution, use pattern and conservation status
Source: J Ethnobiol Ethnomed. 2020 Sep 23;16:56. doi: 10.1186/s13002-020-00405-0 (PMC7513279; doi:10.1186/s13002-020-00405-0)
Supplement: Supplementary file 1 — Additional file 1. Ethnobotanical inventory and some quantitative indexes of NTFPs in Himachal Pradesh. [file 13002_2020_405_MOESM1_ESM.docx]

**Additional file 1: Ethnobotanical inventory and some quantitative indexes of NTFPs in Himachal Pradesh**

| **S.No** | **Botanical Name** | **Vernacular name/Local Name** | **Altitudinal Rande (m)** | **Life Form** | **Part's used** | **Threat Status** | **Indigenous uses** | **UV** | **RFC** | | **CI** | | **CV** | | **RI** | |  |
| --- | --- | --- | --- | --- | --- | --- | --- | --- | --- | --- | --- | --- | --- | --- | --- | --- | --- |
|  | **Acanthaceae** |  |  |  |  |  |  |  |  | |  | |  | |  | |  |
| 1 | *Andrographis paniculata* (Burm. f.) Nees | Kal-megh | 900-1400 | H | Wp |  | Medicinal (Cold) | 0.10 | | 0.30 | | 0.30 | | 0.0050 | | 0.33 | |
| 2 | *Barleria cristata* L. |  | 800-2000 | H | Wp |  | Medicinal (Anaemia, body pain, headache, swellings, toothache); Edible | 0.60 | 0.60 | | 0.24 | | 0.0160 | | 0.67 | |  |
| 3 | *Dicliptera bupleuroides* (Nees) | Ban pipli | 500-2000 | H | Fl |  | Medicinal (Tonsillitis) | 0.10 | 0.30 | | 0.30 | | 0.0050 | | 0.33 | |  |
| 4 | *Dicliptera roxburghiana* Nees |  | 800-2300 | H | Wp |  | Medicinal (Leucorrhoea, tonic, stomach-infection) | 0.30 | 0.60 | | 0.60 | | 0.0200 | | 0.58 | |  |
| 5 | *Justicia adhatoda* L. | Basuti/ Bansa/Pili basuti | upto 1300 | S | Wp |  | Medicinal (Anthelmintic, asthma, antipyretic, sedative, malaria, antiseptic, antispasmodic, constipation, whooping-cough, digestive-disorders, chronic-bronchitis, bronchial-asthma) | 1.20 | 0.90 | | 0.90 | | 0.0450 | | 0.83 | |  |
| 6 | *Justicia japonica* Don |  | 800-2000 | H | Rt, Fr |  | Medicinal (Asthma, boils, child birth, dislocation of joints); Edible; Fodder | 0.60 | 0.30 | | 0.30 | | 0.0050 | | 0.33 | |  |
| 7 | *Justicia simplex* D.Don. | Juffa | 700-1200 | H | Wp |  | Medicinal (Cough, cold, chest pain, pimples, blisters, itching, asthma, fever, flatulence, rheumatism); Fodder | 1.10 | 0.30 | | 0.06 | | 0.0020 | | 0.42 | |  |
| 8 | *Lepidagathis cuspidata* Nees | Billi, Bralu | Upto 1000 | H | Wp |  | Medicinal (Boils, scabies, ulcers, sores, blisters, fever, antioxidant-activity, antifungal-activity) | 0.80 | 0.60 | | 0.60 | | 0.0200 | | 0.58 | |  |
| 9 | *Peristrophe bicalyculata* (Retz.) Nees |  | 700-1800 | H | Wp |  | Medicinal (Eye disorder, fracture, sprain) | 0.30 | 0.30 | | 0.30 | | 0.0050 | | 0.33 | |  |
| 10 | *Strobilanthes atropurpureus* Nees |  | 1300–3600 | H | Infl |  | Medicinal (Diarrhoea) | 0.10 | 0.30 | | 0.04 | | 0.0013 | | 0.42 | |  |
|  | **Adoxaceae** |  |  |  |  |  |  |  |  | |  | |  | |  | |  |
| 11 | *Viburnum mullaha* Buch.-Ham. ex D. Don | Tilhanj, Titmuya | 1500–2200 | T | Fr |  | Fruit-edible | 0.10 | 0.30 | | 0.30 | | 0.0050 | | 0.33 | |  |
|  | **Agaricaceae** |  |  |  |  |  |  |  |  | |  | |  | |  | |  |
| 12 | *Agaricus campestris*L. | Mushroom |  | Mushroom | Wp | Least Concern | Medicinal (Pthisis, aphrodisiac, emaciation rhinitis) | 0.30 | 0.30 | | 0.03 | | 0.0010 | | 0.42 | |  |
|  | **Agavaceae** |  |  |  |  |  |  |  |  | |  | |  | |  | |  |
| 13 | *Agave americana* L. | Ramban | 700-1600 | S | Lf, Wp |  | Medicinal (Boils, burns, constipation, cuts, dropsy, dysentery, fever, goiter, skin diseases, syphilis, veterinary diseases) | 1.10 | 0.30 | | 0.30 | | 0.0050 | | 0.33 | |  |
| 14 | *Agave angustifolia* Haw. | Ramban | 800-1600 | S | Lf | Least Concern | Medicinal (Burns, skin-infections, digestive-disorders) | 0.30 | 0.30 | | 0.30 | | 0.0050 | | 0.33 | |  |
| 15 | *Yucca gloriosa* L. |  | 800-1600 | S | Lf |  | Medicinal (Asthma, ulcers, leprosy) | 0.20 | 0.30 | | 0.30 | | 0.0050 | | 0.33 | |  |
| 16 | *Yucca superba* Roxb |  | 1200-2000 | H | Wp |  | Medicinal (Inflammation, arthritis, blood pressure, gout, diabetes, genitourinary disorders, indigestion, constipation, diuretic, skin cleansing) | 1.00 | 0.30 | | 0.30 | | 0.0050 | | 0.33 | |  |
|  | **Alliaceae** |  |  |  |  |  |  |  |  | |  | |  | |  | |  |
| 17 | *Allium humile* Kunth |  | 3200–3700 | H | Bb |  | Medicinal (Stomach-ache) | 0.10 | 0.60 | | 0.60 | | 0.0200 | | 0.58 | |  |
| 18 | *Allium stracheyi* Baker |  | 3000–4200 | H | Wp |  | Medicinal (Stomach-ache) | 0.10 | 0.60 | | 0.60 | | 0.0200 | | 0.58 | |  |
|  | **Amaranthaceae** |  |  |  |  |  |  |  |  | |  | |  | |  | |  |
| 19 | *Achyranthes aspera* L. | Putkanda | 800-2500 | H | Wp |  | Medicinal (Asthma, boils, bronchitis, cold, cough, dysentery, germicide, headache, laxative leucodemia, piles, pneumonia, rheumatism, scabies, skin diseases, sore, stomachache, tonic, toothache, whooping cough, wounds, veterinary diseases); Edible | 2.20 | 0.90 | | 0.32 | | 0.0486 | | 1.00 | |  |
| 20 | *Achyranthes bidentata* Blume | Puthkanda | 1000-2200 | H | Lf , Wp |  | Medicinal (Constipation, cough fever, blisters, cholera, scorpion-sting, swelling, whooping-cough, blisters in mouth) | 0.80 | 0.60 | | 0.60 | | 0.0200 | | 0.58 | |  |
| 21 | *Amaranthus caudatus* L. | Chaliara | 1000-2600 | H | Lf |  | Medicinal (Boils); Edible | 0.10 | 0.30 | | 0.04 | | 0.0013 | | 0.42 | |  |
| 22 | *Amaranthus paniculatus* L. | Seul | 800-2000 | H | Sd |  | Medicinal (Diarrohea, blood purifier, piles, sores, diuretic) | 0.50 | 0.60 | | 0.60 | | 0.0200 | | 0.58 | |  |
| 23 | *Amaranthus spinosus* L. |  | 1200-1800 | H | Wp |  | Medicinal (Digestion, snake-bite) | 0.20 | 0.60 | | 0.60 | | 0.0200 | | 0.58 | |  |
| 24 | *Amaranthus viridis* L. | Sariyara | 1200-2300 | H | Ap |  | Medicinal (Stomach-disorders) | 0.10 | 0.30 | | 0.30 | | 0.0050 | | 0.33 | |  |
| 25 | *Cyathula capitata* Moq. | Litra | 1500- 2500 | H | Lf, Sd, Rt |  | Medicinal (Emetic, abortifacient, veterinary diseases, diarrhea excessive-menustration, Emetic, abortifacient, Skin diseases) | 0.70 | 0.60 | | 0.60 | | 0.0200 | | 0.58 | |  |
| 26 | *Cyathula tomentosa* (Roth.) Moq. | Kutha | 1000- 1400 | S | Lf, Fr, Sd |  | Medicinal (Skin diseases, Liver protective); Fodder | 0.30 | 0.30 | | 0.04 | | 0.0013 | | 0.42 | |  |
| 27 | *Deeringia amaranthoides* (Lam.) Merr. |  | 700-1200 | S | Lf, Fr |  | Medicinal (Sores) | 0.10 | 0.30 | | 0.30 | | 0.0050 | | 0.33 | |  |
|  | **Anacardiaceae** |  |  |  |  |  |  |  |  | |  | |  | |  | |  |
| 28 | *Cotinus coggygria* Scop. |  | 700-1300 | S | Fr, Fl |  | Medicinal (Astringent); Dye | 0.20 | 0.30 | | 0.04 | | 0.0013 | | 0.42 | |  |
| 29 | *Lannea coromandelica* (Houtt.) Merr. |  | 700-1400 | T | Bk, Lf |  | Medicinal (Astringent, bruises, skin eruptions, heart diseases, dysentery, mouth sores, toothache, swellings, pains) | 0.90 | 0.30 | | 0.30 | | 0.0050 | | 0.33 | |  |
| 30 | *Mangifera indica* L. | Amb | 700-1800 | T | Lf, fr, Sd |  | Medicinal (Abortifacient, cancer, anasarca, antifertility, dropsy, fever, cholera, diarrohea, digestion, tonic, dysentery, eye complaints, jaundice, laxative, rheumatism, scabies, stomachache, toothache); Edible; Fuel; Religious, (Sculptures on temples, pillars, with maidens of Jain, Hindu called (Vrikshikas), Abode of God, decoration on festivals and newly build houses | 2.80 | 0.30 | | 0.01 | | 0.0004 | | 0.58 | |  |
| 31 | *Pistacia integerrima* Stew. Ex | Kakarsingi | 1200-1600 | T | Lf |  | Medicinal (Tonic, expectorant, cough, cold, asthma, fever, appetite, chronic pulmonary affection, dyspeptic, hepatitis, liver disorders, infections, diabetes, pain, inflammatory conditions, jaundice, snake bites, vomiting and diarrohea); Edible; Fodder; Timber; Dye | 2.20 | 1.20 | | 0.30 | | 0.0806 | | 1.33 | |  |
| 32 | *Rhus javanica* L. | Titri | 2000-2500 | T | Twig, Fr, Lf | Least Concern | Medicinal (Gastric problem, skin diseases, cholera, indigestion, purgative, stomachache) | 0.60 | 0.60 | | 1.00 | | 0.0333 | | 0.58 | |  |
|  | **Apiaceae** |  |  |  |  |  |  |  |  | |  | |  | |  | |  |
| 33 | *Anethum graveolens* L. | Kadwisaunf |  | H | Sd |  | Medicinal (bloating) | 0.10 | 0.30 | | 0.30 | | 0.0050 | | 0.33 | |  |
| 34 | *Angelica glauca*Edgew. | Chora | 2700-3800 | H | Rt | Endangered | Medicinal (Fever, cold, dysentery, gastric complaints, menorrhoea, stomach complaints, vomiting) | 0.70 | 1.20 | | 0.63 | | 0.0840 | | 1.17 | |  |
| 35 | *Apium leptophyllum* (Pers.) F. Muell. ex Benth. |  | 1100-1800 | H | Fr |  | Medicinal (Germicide, disinfectant, antiseptic); Flavoring agent in food products | 0.40 | 0.30 | | 0.06 | | 0.0020 | | 0.42 | |  |
| 36 | *Bunium persicum*(Boiss.) B. Fedtsch. | Kala zira | 3000–4000 | H | Fr |  | Medicinal (Liver complaints, gastric) | 0.20 | 0.30 | | 0.30 | | 0.0050 | | 0.33 | |  |
| 37 | *Bupleurum hamiltonii* N.P.Balakr. |  | 900-2000 | H | Ap, Rt |  | Medicinal (Diaphoretic, antiseptic, anti- inflammatory, liver complaints) | 0.40 | 0.30 | | 0.30 | | 0.0050 | | 0.33 | |  |
| 38 | *Bupleurum lanceolatum* Wall. ex DC. |  | 1500–2500 | H | Fr |  | Medicinal (Snake bite) | 0.10 | 0.30 | | 0.30 | | 0.0050 | | 0.33 | |  |
| 39 | *Bupleurum longicaule* var. himalayense (Kl.) Cl. | | 3300–4500 | H | Wp |  | Medicinal (Colic, gastrointestinal diseases, renal complication) | 0.30 | 0.30 | | 0.30 | | 0.0050 | | 0.33 | |  |
| 40 | *Bupleurum tenue* Buch.-Ham. ex D.Don |  | 1000-1600 | H | Wp |  | Fodder | 0.10 | 0.30 | | 0.30 | | 0.0050 | | 0.33 | |  |
| 41 | *Bupleurum thompsoni* Cl. |  | 2000–3000 | H | Wp |  | Medicinal (Stomach ache) | 0.10 | 0.30 | | 0.30 | | 0.0050 | | 0.33 | |  |
| 42 | *Carum carvi* L. | Singoo (Carwey seed)/Shahi Jeera/ Shingu Zira | 2500-4100 | H | Sd |  | Medicinal (Abortifacient, anthelmintic, aromatic, cardic, carminative, stimulant, stomachic and tonic); Spice and flavouring agent; Perfumes | 0.90 | 0.30 | | 0.01 | | 0.0004 | | 0.58 | |  |
| 43 | *Centella asiatica* (L.) Urb. | Brahmi | 700-1500 | H | Wp | Least Concern | Medicinal (Antihelmintic, brain tonic, cholera, cough, diarrohea, diuretic, dysentery, eye complaints, fever, headache, leprosy, liver complaints, nervine diseases, respiratory diseases, skin diseases, stomachache, epilepsy, tonic for brain, syphilis, tumor, urine complaints, wounds) | 2.20 | 0.90 | | 0.90 | | 0.0450 | | 0.83 | |  |
| 44 | *Chaerophyllum reflexum* Lindl. |  | 1700-2000 | H | Rt |  | Edible | 0.10 | 0.30 | | 0.30 | | 0.0050 | | 0.33 | |  |
| 45 | *Cortia depressa (Don)* Norm |  | 3300–4900 | H | Wp |  | Medicinal (Abdominal diseases, antiinflammatory, rheumatism, sedative, stomach ache) | 0.50 | 0.30 | | 0.30 | | 0.0050 | | 0.33 | |  |
| 46 | *Ferula jaeschkeana* (L.) Vatke |  | 2800–3800 | H | Rt |  | Medicinal (Rheumatism, toothache, wounds, poultices) | 0.40 | 0.30 | | 0.30 | | 0.0050 | | 0.33 | |  |
| 47 | *Heracleum candicans* Wall. | Patishan/ Patrala | 1800-3300 | H | St, Rt |  | Medicinal (leucoderma, eczema, itches, stomach disorders (liver complaints), arthritis and toothache, aphrodisiac nerve tonic, intestinal parasites, abdominal colic, digestive and flatulence complaints, phlegm and wind disorders, earache, bleeding, leprosy, fever due to wounds, blood pressure, Eczema, ring worm infection, leucoderma, menstrual disorder, piles); Fodder | 2.10 | 0.90 | | 0.56 | | 0.0560 | | 0.92 | |  |
| 48 | *Heracleum lanatum* Michx. | Patishan/ Patrala | 2500–4500 | H | Rt, Fr, Wp |  | Medicinal (Menstrual complaints, leucoderma, piles) | 0.30 | 0.60 | | 0.60 | | 0.0200 | | 0.58 | |  |
| 49 | *Heracleum nepalense* D.Don |  | 1800–3600 | H | Rt |  | Medicinal (Menstrual complications, leucoderma) | 0.20 | 0.30 | | 0.30 | | 0.0050 | | 0.33 | |  |
| 50 | *Hydrocotyle javanica* Thunb |  | 800- 2000 | H | Wp, Lf | Least concern | Medicinal (Brain tonic, cures tonsils, dysentery, indigestion, fever) | 0.50 | 0.60 | | 0.20 | | 0.0067 | | 0.58 | |  |
| 51 | *Pimpinella acuminata* (Edgew.) Cl. |  | 2500- 3700 | H | Lf |  | Medicinal (Dysentery) | 0.10 | 0.30 | | 0.10 | | 0.0017 | | 0.33 | |  |
| 52 | *Pimpinella diversifolia* DC. |  | 1200-3500 | H | Rt, Wp |  | Medicinal (Cold, cough, digestive disorder); Edible | 0.40 | 0.60 | | 0.60 | | 0.0200 | | 0.58 | |  |
| 53 | *Pleurospermum angelicoides* (DC.) Cl. |  | 2500–4000 | H | Rt |  | Medicinal (Antihelminthic, gastric, stomach ache) | 0.30 | 0.60 | | 0.60 | | 0.0200 | | 0.58 | |  |
| 54 | *Pleurospermum brunonis* Benth. ex C.B.Clarke | Hewan | 3300-4500 | H | Wp |  | Medicinal (Skin infection) | 0.10 | 0.30 | | 0.06 | | 0.0020 | | 0.42 | |  |
| 55 | *Pleurospermum candollii* (DC.) Cl. |  | 3500–4200 | H | Fr |  | Medicinal (Dyspepsia, renal pain, stomach ache) | 0.30 | 0.60 | | 0.20 | | 0.0067 | | 0.58 | |  |
| 56 | *Pleurospermum densiflorum* Hk.f. | Nesar/Lossar | 3000–4000 | H | Wp |  | Medicinal (Cough, cold); Incense; Insecticide; Essential oil; Perfumery industry | 0.60 | 0.30 | | 0.06 | | 0.0050 | | 0.67 | |  |
| 57 | *Selinum tenuifolium* Salisb. | Bhutkesi | 2500-4800 | H | Rt |  | Medicinal (Swelling, nervine, sedative); Incense; Insecticidal | 0.50 | 0.60 | | 0.08 | | 0.0080 | | 0.75 | |  |
| 58 | *Selinum vaginatum* (Edgew.) C. B. Cl. | Bhutkesi | 3000-3500 | H | Rt |  | Medicinal (Nervine tonic, sedative, skin diseases, antibacterial and analgesic, wounds, nervine sedative, hysteria, dysmenorrheal, liquor preparation); Incense | 1.00 | 0.60 | | 0.20 | | 0.0133 | | 0.67 | |  |
| 59 | *Trachydium roylei* L. |  | 1000-2000 | H | Ap |  | Fodder | 0.10 | 0.30 | | 0.30 | | 0.0050 | | 0.33 | |  |
| 60 | *Trachyspermum ammi* (L.) Sprague | Anjwan | 1500-2500 | H | Fr |  | Medicinal (Antimicrobial activity; essential oil; nosocomial infection, relieving flatulence, dyspepsia, spasmodic disorders, common cold, acute pharyngitis, sore and congested throat) | 0.90 | 0.30 | | 0.20 | | 0.0067 | | 0.42 | |  |
|  | **Apocynaceae** |  |  |  |  |  |  | 0.00 | 0.00 | | 0.00 | | 0.0000 | |  | |  |
| 61 | *Calotropis procera* (Aiton) Dryand. | Aak |  | S |  |  | Medicinal (Jaundice) | 0.10 | 0.30 | | 0.30 | | 0.0050 | | 0.33 | |  |
| 62 | *Carissa opaca* Stapf ex Haines | Garnoin | 700-1500 | S | Lf, Fr |  | Fodder; Edible | 0.20 | 0.30 | | 0.08 | | 0.0027 | | 0.42 | |  |
| 63 | *Catharanthus roseus* (L.) G.Don |  | 700-1500 | S | Wp, Rt, Lf |  | Medicinal (Cancer, diabetes, menon^-^hea, body vomiting, anti-cancer, purgative, vennifuge, swelling, depurative, hemostatic, antibacterial, antifungal, antiviral); Misc. (Ornamental) | 1.40 | 0.30 | | 0.04 | | 0.0013 | | 0.42 | |  |
| 64 | *Rauwolfia serpentina* (L.) Benth. ex Kurz | Sarpagandha | Upto 1000 | S | Rt |  | Medicinal (Antihelminthic, antidote, blood pressure, fever, stomach trouble) | 0.50 | 0.60 | | 0.60 | | 0.0200 | | 0.58 | |  |
| 65 | *Vinca rosea L.* | S adab ahar | 700-1800 | H | Wp |  | Medicinal (Hypotensive, sedative, cancer, muscle pain) | 0.40 | 0.30 | | 0.30 | | 0.0050 | | 0.33 | |  |
|  | **Araceae** |  |  |  |  |  |  |  |  | |  | |  | |  | |  |
| 66 | *Acorus calamus* L. | Bach, Bahere, Bajh, Goodverch | 1100-2600 | H | Rf, Lf | Least Concern | Medicinal (Abdominal-pain, asthma, bodyache, bronchitis, cold, cough, constipation, cuts, dysentery, epilepsy, headache, hysteria, indigestion, inflation, malaria, neck-pain, skin-disease, snake-bite, stomachache, tonic, insecticidal, fever, asthma, carminative) | 2.40 | 1.20 | | 1.20 | | 0.0800 | | 1.08 | |  |
| 67 | *Arisaema flavum* (Forsk.) Schott | Kira aloo | 1200-2000 | H | Bb |  | Medicinal (Skin disease, veterinary diseases) | 0.20 | 0.30 | | 0.30 | | 0.0050 | | 0.33 | |  |
| 68 | *Arisaema jacquemontii* Bl |  | 2200- 3300 | H | Bb | Least Concern | Medicinal (Sprains and regains muscular strength) | 0.20 | 0.60 | | 0.60 | | 0.0200 | | 0.58 | |  |
| 69 | *Arisaema tortuosum* (Wall.) Schott | Shaungal/ Leetu/Galgal | 1800-2500 | H | Tb, Wp |  | Medicinal (Veterinary diseases); Edible | 0.20 | 0.90 | | 0.20 | | 0.0200 | | 0.92 | |  |
| 70 | *Colocasia affinis* Schott |  | 1200- 1700 | H | Bb |  | Medicinal (Jaundice) | 0.10 | 0.30 | | 0.30 | | 0.0050 | | 0.33 | |  |
| 71 | *Sauromatum venosum* Kunth. | Kidachali | 1000-2000 | H | Tu | Least Concern | Medicinal (Skin disease, tumors, veterinary sores); Edible | 0.30 | 0.30 | | 0.04 | | 0.0013 | | 0.42 | |  |
|  | **Araliaceae** |  |  |  |  |  |  |  |  | |  | |  | |  | |  |
| 72 | *Aralia cachemirica* Decne. | Bal chora | 2500–4000 | H | Lf , Rf |  | Medicinal (Gastric complaints) | 0.10 | 0.90 | | 0.90 | | 0.0450 | | 0.83 | |  |
| 73 | *Hedera nepalensis* Koch. | Katari | 900-2000 | S | Fr, Lf |  | Medicinal (Cold, cough, stimulant, diaphoretic, cathartic, rheumatism); Edible | 0.70 | 0.30 | | 0.08 | | 0.0027 | | 0.42 | |  |
|  | **Arecaceae** |  |  |  |  |  |  |  |  | |  | |  | |  | |  |
| 74 | *Phoenix sylvestris* (Linn.) Roxb. |  | 700-1700 | S | Lf, Fr |  | Medicinal (Cardiotonic, constipation, heart complaints, abdominal complaints, fevers, vomiting, toothache) | 0.70 | 0.30 | | 0.30 | | 0.0050 | | 0.33 | |  |
| 75 | *Zalacca beccarii* Hk.f. |  | Up to 1000 | T | St, Rt |  | Medicinal (Stomach disorder) | 0.10 | 0.30 | | 0.30 | | 0.0050 | | 0.33 | |  |
|  | **Asclepiadaceae** |  |  |  |  |  |  |  |  | |  | |  | |  | |  |
| 76 | *Asclepias curassavica* L. |  | 700-1500 | S | Lf, Rt |  | Medicinal (Emetic, laxative, abortifacient, expectorant, pneumonia, pleurisy, lung problems, ringworm, stop bleeding, fever, intestinal, troubles, diaphoretic, anthelmintic, purgative, stomach tumours, piles, gonorrhoea, warts); Fodder | 2.00 | 0.30 | | 0.09 | | 0.0030 | | 0.42 | |  |
| 77 | *Ceropegia bulbosa* Roxb. |  | Up to 1500 | H | Tb |  | Medicinal (For vitality) | 0.10 | 0.30 | | 0.30 | | 0.0050 | | 0.33 | |  |
| 78 | *Cryptolepis buchanani* Roem. & Schult. | Taern | 1100-1900 | S | Wp |  | Medicinal (Rickets, abdominal pain, anasarca, bodyache, cholera, dropsy, dysentery, cuts, stomachache, venereal diseases); Fibre | 1.10 | 0.30 | | 0.06 | | 0.0020 | | 0.42 | |  |
| 79 | *Marsdenia roylei* Wight |  | 700-1500 | H | Wp |  | Medicinal (Cold, eye complaints, gonorrhea) | 0.30 | 0.30 | | 1.00 | | 0.0167 | | 0.33 | |  |
|  | **Asparagaceae** |  |  |  |  |  |  |  |  | |  | |  | |  | |  |
| 80 | *Asparagus adscendens* Roxb. | Shatavari/ Sanspai/ Safed Musali | upto 1400 | H | Rt |  | Medicinal (Nausea, vomiting, headache, aphrodisiac, appetizer, astringent, leucorrhoea, coolant for cattle) | 0.80 | 0.60 | | 0.60 | | 0.0200 | | 0.58 | |  |
| 81 | *Asparagus filicinus* Buch.-Ham ex Roxb |  | 2000-3200 | S | Fr, Rt |  | Medicinal (Urinary problems, Antihelminthic, aphrodisiac, rheumatism, bleeding from nose, blood in urine, cough, diarrhoea, dysentery, febrifuge, gonorrhoea, headache, menstrual, gastric); Religious | 1.50 | 0.60 | | 0.60 | | 0.0200 | | 0.58 | |  |
| 82 | *Asparagus racemosus* Willd. |  | 1100-1600 | S | Rt |  | Medicinal (Anthelmintic, aphrodisiac, rheumatism, bleeding from nose, cough, dysentery, febrifuge, gastric complaints, gonorrhea, headache, menstrual complaints, snake bite, stomachache, tonic, urine complaints, infertility, decreased libido, miscarriage, menopause, leucorrhea, sexual debility, impotence, spermatorrhea, inflammation of sexual organs, hyperacidity, stomach ulcers, dysentery, and bronchial infections, uterine tonic, galactogogue, general health tonic); | 3.10 | 0.90 | | 0.90 | | 0.0450 | | 0.83 | |  |
|  | **Asteraceae** |  |  |  |  |  |  |  |  | |  | |  | |  | |  |
| 83 | *Achillea millefolium*L. | Dhooplakar | 3000-6000 | H | Fl, Wp | Least Concern | Medicinal ( Diphtheria, epilepsy, fever, gastric complaints, hysteria, piles, stimulant, toothache, ulcer, bleeding piles, kidney diseases, atonic dyspepsia, carminative, tonic, cough, cold); Insect repellent; Aromatic | 1.80 | 0.90 | | 0.14 | | 0.0210 | | 1.00 | |  |
| 84 | *Ageratum conyzoides* L. | Okalbuti | 700-1700 | H | Lf, Rt, Sd, Fr, Fl |  | Medicinal (Antiseptic, boils, bums, cancer, cuts, diarrohea, headache, leprosy,muscularpain, piles, ringworm, scabies, snake bite, sores, swellings, tumor, uterine disorders) | 1.70 | 0.30 | | 0.30 | | 0.0050 | | 0.33 | |  |
| 85 | *Ageratum houstonianum* Mill. | Okalbuti | 700-1300 | H | Wp |  | Medicinal (Diarrohea, tonic, skin diseases) | 0.30 | 0.30 | | 0.30 | | 0.0050 | | 0.33 | |  |
| 86 | *Ainsliaea aptera* DC. | Sath jalori | 1500-3500 | H | Rt |  | Medicinal (Stomachache, gastric problems) | 0.20 | 0.90 | | 0.90 | | 0.0450 | | 0.83 | |  |
| 87 | *Anaphalis busua* (Buch.-Ham.) DC. | Bacha | 1600-2200 | H | Lf |  | Medicinal (Anodyne, antiseptic, astringent, expectorant, sedative, burns, sores, ulcers, bruises, swellings and rheumatic joints) | 0.60 | 0.30 | | 0.08 | | 0.0027 | | 0.42 | |  |
| 88 | *Arctium lappa* L. | Jungli Kuth/ Nakli kuth | 2100-3700 | H | Rt |  | Medicinal (Diuretic, arexigenic, cutaneous eruptions, rheumatism, cytitis, gout and specifically for eczema blood sugar and psoriasis) | 0.70 | 0.30 | | 0.30 | | 0.0050 | | 0.33 | |  |
| 89 | *Artemisia absinthium* L. | Jaue | 2000-3500 | H | Lf |  | Medicinal (Dandruff, wounds, vermifuge) | 0.30 | 0.30 | | 0.30 | | 0.0050 | | 0.33 | |  |
| 90 | *Artemisia brevifolia* Wall. ex DC. | Seski | 2000-3500 | H | Ap |  | Medicinal (appetizing, diuretic, aphrodisiac, laxative, astringent, useful in dysentery, diarrhoea, throat complaints and leprosy) | 0.80 | 0.30 | | 0.30 | | 0.0050 | | 0.33 | |  |
| 91 | *Artemisia capillaris* Thunb. | Malaria buti | 1000-4300 | H | Wp |  | Medicinal (Spleen, stomach, liver, gall bladder) | 0.40 | 0.30 | | 0.30 | | 0.0050 | | 0.33 | |  |
| 92 | *Artemisia dracunculus* L |  | 2700-4700 | H | Ap , Lf |  | Medicinal (Anti-inflammatory, hepatoprotective, antihyperglycemic effects) | 0.30 | 0.30 | | 0.30 | | 0.0050 | | 0.33 | |  |
| 93 | *Artemisia japonica* Pamp. | Chamber | 1200-1700 | H | Lf |  | Medicinal (Decoction, throat infections, vaginitis, skin diseases) | 0.40 | 0.30 | | 0.30 | | 0.0050 | | 0.33 | |  |
| 94 | *Artemisia maritima* L. | Shoma, Atong | 2600–4500 | H | Wp |  | Medicinal (Antihelminthic, cuts, gastric complaints, blood purifier) | 0.40 | 0.30 | | 0.30 | | 0.0050 | | 0.33 | |  |
| 95 | *Artemisia nilagirica* (C.B. Clarke) | Charmara | Upto 1800 | S | Lf |  | Medicinal (Antiallergic, headache, menstrual problem, repellent, digestive disorders); Religious | 0.60 | 0.30 | | 0.06 | | 0.0020 | | 0.42 | |  |
| 96 | *Artemisia parviflora* Roxb. | Jhau | 1400-2000 | H | Lf, Rt, Sd |  | Medicinal (Carminative, wormifuge, throat problems); Fodder | 0.40 | 0.30 | | 0.30 | | 0.0050 | | 0.33 | |  |
| 97 | *Artemisia roxburghiana* Bess |  | 2400–5600 | H | Lf |  | Medicinal (Eczema, pimples, sores) | 0.30 | 0.60 | | 0.60 | | 0.0200 | | 0.58 | |  |
| 98 | *Artemisia scoparia* Waldst and Kit. |  |  | H | Lf |  | Medicinal (Bums, cold, headache, earache, constipation, pain, burns, depurative, jaundice, hepatitis, inflammation of the gall bladder); Fodder | 1.20 | 0.30 | | 0.04 | | 0.0013 | | 0.42 | |  |
| 99 | *Artemisia sieversiana* Willd. | Charmara | 2500-3200 | H | Lf , Wp |  | Medicinal (Abortifacient, wounds to cure pain and swelling) | 0.30 | 0.30 | | 0.30 | | 0.0050 | | 0.33 | |  |
| 100 | *Aster tibeticus* Hk.f. |  | 3000–4000 | H | Rt |  | Medicinal (Malaria) | 0.10 | 0.30 | | 0.03 | | 0.0005 | | 0.33 | |  |
| 101 | *Bidens biternata* (Lour.) Merr. & Sherff | Badigumbri | 700-1900 | H | Fr, Lf, Fl, Rt |  | Medicinal (Appetizer, cough, cuts, inflammation, snake bite, sores, toothache, ulcers); Edible | 0.90 | 0.30 | | 0.02 | | 0.0007 | | 0.42 | |  |
| 102 | *Bidens  pilosa* L |  | 1000-2000 | H | Wp |  | Medicinal (Cough, cuts, ear and eye complaints, headache, inflammation, leprosy, skin diseases, snake bite, sores, wounds); Edible | 1.10 | 0.30 | | 0.30 | | 0.0050 | | 0.33 | |  |
| 103 | *Blumea laciniata* (Roxb.) DC. |  | 1200-1700 | H | Lf |  | Medicinal (Eczema, skin disease) | 0.20 | 0.30 | | 0.30 | | 0.0050 | | 0.33 | |  |
| 104 | *Carduus edelbergii* Reich.f. |  | 2700–4000 | H | Wp |  | Medicinal (Blood purifier, diuretic, tonic) | 0.30 | 0.30 | | 0.30 | | 0.0050 | | 0.33 | |  |
| 105 | *Chrysanthemum indicum* L. | Guldaudi | 700-2500 | H | Lf |  | Medicinal (Boils) | 0.10 | 0.25 | | 0.30 | | 0.0050 | | 0.38 | |  |
| 106 | *Cirsium wallichii* DC. | Bursa | 1800-2500 | H | Rt, Wp |  | Medicinal (Gastric problems) | 0.10 | 0.30 | | 0.30 | | 0.0050 | | 0.33 | |  |
| 107 | *Conyza japonica* (Thunb.) Less. ex Less. | Gaadi | 1000-1800 | H | Wp |  | Medicinal (Killing or expelling worms from the body) | 0.10 | 0.30 | | 0.30 | | 0.0050 | | 0.33 | |  |
| 108 | *Conyza stricta* Willd. |  | 700-2000 | H | Wp |  | Medicinal (Bone fracture, swellings) | 0.20 | 0.30 | | 0.30 | | 0.0050 | | 0.33 | |  |
| 109 | *Cosmos caudatus* Kunth |  | 900-1400 | H | Lf |  | Medicinal (Blood circulation, potent antioxidants) | 0.20 | 0.30 | | 0.30 | | 0.0050 | | 0.33 | |  |
| 110 | *Dichrocephala bicolor* (Roth) Schltdl. |  | 700-1600 | H | Rt |  | Medicinal (Antihypertensive agent) | 0.10 | 0.30 | | 0.30 | | 0.0050 | | 0.33 | |  |
| 111 | *Eclipta alba* (L.) Hassk. | Bringraj | 700-1500 | H | Wp | LC | Medicinal (Antifertility, asthma, bronchitis, fever, headache, itching, jaundice, leucoderma, swelling, toothache, antiseptic) | 1.10 | 0.30 | | 0.30 | | 0.0050 | | 0.33 | |  |
| 112 | *Elephantopus scaber* L. |  | 700-1600 | H | Lf, Rt |  | Medicinal (Abortificant, amoebic dysentery, blood purifier) | 0.30 | 0.30 | | 0.30 | | 0.0050 | | 0.33 | |  |
| 113 | *Erigeron bellidioides* (Don) Benth. |  | 1400–4300 | H | Wp |  | Medicinal (Blood purifier) | 0.10 | 0.30 | | 0.03 | | 0.0005 | | 0.33 | |  |
| 114 | *Erigeron bonariensis* L. |  | 800-1700 | H | Lf |  | Medicinal (Rheumatism, mouth, throat and skin diseases) | 0.30 | 0.60 | | 0.50 | | 0.0167 | | 0.58 | |  |
| 115 | *Erigeron canadensis* L. syn. | Koda |  | H | Lf |  | Medicinal (stimulant, astringent, diuretic and to treat dysentery, diarrhea and uterine haemorrhage, bronchial catarrh and cystitis); Fodder | 0.60 | 0.60 | | 0.08 | | 0.0075 | | 0.75 | |  |
| 116 | *Gerbera gossypina* (Royle) Beauv. | Bacha | 1300-2200 | H | Rt |  | Medicinal (Blood pressure, gastric disorders, measles) | 0.20 | 0.60 | | 0.60 | | 0.0200 | | 0.58 | |  |
| 117 | *Inula cappa* (Buch.-Ham. ex D.Don) DC. |  | 1000-1900 | Sh | Lf |  | Medicinal (Headache, urinary complaints); Fodder | 0.30 | 0.30 | | 0.02 | | 0.0007 | | 0.42 | |  |
| 118 | *Inula cuspidata* Hk.f. |  | 900-1800 | Sh | Lf |  | Fodder | 0.10 | 0.30 | | 0.30 | | 0.0050 | | 0.33 | |  |
| 119 | *Inula racemosa* Hook.f. | Manu | 2500-3699 | H | Rt, Fl |  | Medicinal (Gastrointestinal, rheumatism); Incense | 0.30 | 0.30 | | 0.06 | | 0.0020 | | 0.42 | |  |
| 120 | *Inula royleana* Cl. |  | 2100–4000 | H | Wp |  | Medicinal (Dermitis, stimulant, high blood pressure) | 0.30 | 0.30 | | 0.30 | | 0.0050 | | 0.33 | |  |
| 121 | *Jurinella macrocephala* (Royle) Aswal & Goel | Dhoop | 3000-4500 | H | Rt |  | Medicinal (Antiseptic, colic, child birth, laxative, skin eruption) | 0.50 | 0.30 | | 0.30 | | 0.0050 | | 0.33 | |  |
| 122 | *Ligularia jacquemontiana* (Decne) Rau |  | 3200–4000 | H | Rt |  | Medicinal (Unripe boils) | 0.00 | 0.30 | | 0.30 | | 0.0050 | | 0.33 | |  |
| 123 | *Parthenium hysterophorus* L. | Chikadu | 700-1600 | H | Wp |  | Medicinal (Tonic, analgesic, tonic, febrifuge, amniotic, dysentery) | 0.60 | 0.30 | | 0.30 | | 0.0050 | | 0.33 | |  |
| 124 | *Saussurea gossypiphora* D. Don |  | 3800–5600 | H | Wp |  | Medicinal (Burns, cuts) | 0.20 | 0.30 | | 0.30 | | 0.0050 | | 0.33 | |  |
| 125 | *Saussurea graminifolia* Wall. |  | 3500–5000 | H | Wp |  | Medicinal (Headache, fever) | 0.20 | 0.30 | | 0.30 | | 0.0050 | | 0.33 | |  |
| 126 | *Saussurea heteromalla* (D.Don) Hand.-Maz. |  | 3000–4000 | H | Sd |  | Medicinal (Carminative, horse bite, Leucoderma, wounds, colic) | 0.50 | 0.60 | | 0.08 | | 0.0027 | | 0.58 | |  |
| 127 | *Saussurea obvallata* (DC.) Sch. – Bip. |  | 3600–4500 | H | Wp, Rt |  | Medicinal (Burns, cuts) | 0.20 | 0.60 | | 0.12 | | 0.0040 | | 0.58 | |  |
| 128 | *Saussurea simpsoniana* (Field & Gard.) Lipsch | | 3800–5600 | H | Infl |  | Medicinal (Fever, snakebite) | 0.20 | 0.30 | | 0.03 | | 0.0005 | | 0.33 | |  |
| 129 | *Saussurea auriculata* (DC.) Sch.-Bip |  | 3000–3800 | H | Lf |  | Medicinal (Renal diseases) | 0.10 | 0.30 | | 0.30 | | 0.0050 | | 0.33 | |  |
| 130 | *Saussurea bracteata* Decne. |  | 3800–4800 | H | Wp |  | Medicinal (Cold, cough, fever) | 0.30 | 0.30 | | 0.30 | | 0.0050 | | 0.33 | |  |
| 131 | *Saussurea ceratocarpa* Decne |  | 3500–5000 | H | Wp |  | Medicinal (Colic, headache, lumbar pain, renal pain) | 0.40 | 0.30 | | 0.30 | | 0.0050 | | 0.33 | |  |
| 132 | *Senecio chrysanthemoides* DC. |  | 2000-3600 | H | Wp |  | Medicinal (Inflammation of mouth, sore throat) | 0.20 | 0.30 | | 0.30 | | 0.0050 | | 0.33 | |  |
| 133 | *Senecio graciliflorus* (L.) DC. | Zerjum | 3200-4000 | H | Wp |  | Medicinal (Insect bites, ringwonn diseases, earache) | 0.30 | 0.60 | | 0.60 | | 0.0200 | | 0.58 | |  |
| 134 | *Senecio nucficaulis* Buch.- Ham. |  | 1200-2000 | H | Rt |  | Medicinal (Cough, cold) | 0.20 | 0.30 | | 0.30 | | 0.0050 | | 0.33 | |  |
| 135 | *Senecio nudicaulis* Buch.-Ham. ex D. Don |  | 1200-3350 | H | Ap |  | Medicinal (colic, fever, skin diseases) | 0.30 | 0.60 | | 0.60 | | 0.0200 | | 0.58 | |  |
| 136 | *Senecio rufinervis* DC. | Dhuda | 1800−3500 | H | Wp |  | Medicinal (Cure fever, perfumery) | 0.20 | 0.30 | | 0.30 | | 0.0050 | | 0.33 | |  |
| 137 | *Sigesbeckia orientalis* L. |  | 700-1800 | H | Wp |  | Medicinal (Boils, sores, ulcer, cardiac, skin diseases) | 0.50 | 0.30 | | 0.30 | | 0.0050 | | 0.33 | |  |
| 138 | *Sonchus oleraceus* L. |  | 1500-2000 | H | Fl, Ap, Lf , Lt |  | Medicinal (Cuts, injuries, febrifuge, jaundice, galactagogue, liver complaints, tonic) | 0.70 | 0.30 | | 0.30 | | 0.0050 | | 0.33 | |  |
| 139 | *Spilanthes acmella* (L.) L. | Karkara | 300-2300 | H | Lf , Fl |  | Medicinal (Purify blood, high blood pressure) | 0.20 | 0.30 | | 0.30 | | 0.0050 | | 0.33 | |  |
| 140 | *Tagetes minuta* L. | Marigold grass, Jungli gainda | 1000-2400 | H | Fl |  | Medicinal (Anti-viral, ranikhet,asthmatic, aperients, diuretic and diaphoretic, tranquilizing, hypotensive, spasmolytic, bronchodilatory, anti- inflammatory properties); Insect repellent | 1.10 | 0.90 | | 0.04 | | 0.0054 | | 1.00 | |  |
| 141 | *Tanacetum gracile* Hk.f. & Th. |  | 2800–3600 | H | Wp |  | Medicinal (Febrifuge) | 0.10 | 0.30 | | 0.30 | | 0.0050 | | 0.33 | |  |
| 142 | *Tanacetum tenuifolium* Jacq. |  | 3400–4200 | H | Wp |  | Medicinal (Angina, body ache, headache, renal colic) | 0.40 | 0.30 | | 0.30 | | 0.0050 | | 0.33 | |  |
| 143 | *Tanacetum tomentosum* DC. |  | 3500–4500 | H | Wp |  | Medicinal (Colic, diarrhoea, earache) | 0.30 | 0.30 | | 0.30 | | 0.0050 | | 0.33 | |  |
| 144 | *Tanacetum dolichophyllum* (Kitam.)Kitam. | Dhoop/Guggul | 3000-4400 | H | Wp |  | Medicinal (Fever, headache, body ache, kill intestinal worms, calm the nerves) | 0.50 | 0.30 | | 0.30 | | 0.0050 | | 0.33 | |  |
| 145 | *Taraxacum officinale* Weber ex Wigg. | Dhudhi/Dandelion | 300-5400 | H | Rt |  | Medicinal (Blisters, blood purifier, bowel complaints, diuretic, dysentery, gastric, ulcer, headache, backache, kidney diseases, liver complaints, tonic, wounds); Edible | 1.40 | 0.60 | | 0.12 | | 0.0080 | | 0.67 | |  |
| 146 | *Tridax procumbens* L. |  | 900-1500 | H | Wp |  | Medicinal (Antiseptic, blister, boils, cuts, diarrohea, dysentery, eczema, eye disease, fever, leprosy, scorpion bite, skin disease, sores, stomachache, stone in urine bladder, toothache, ulcer) | 1.70 | 0.30 | | 0.30 | | 0.0050 | | 0.33 | |  |
|  | **Athyriaceae** |  |  |  |  |  |  |  |  | |  | |  | |  | |  |
| 147 | *Diplazium esculentum* (Retz.) Sw. | Linger | 800-1800 | Fern | Frd |  | Medicinal (Constipation); Edible | 0.20 | 0.30 | | 0.04 | | 0.0013 | | 0.42 | |  |
| 148 | *Diplazium maximum* (D.Don) C. Chr. | Khasrod | 1400-2400 | Fern | Wp |  | Medicinal (Body pain); Edible | 0.20 | 0.30 | | 0.04 | | 0.0013 | | 0.42 | |  |
|  | **Balanophoraceae** |  |  |  |  |  |  |  |  | |  | |  | |  | |  |
| 149 | *Balanophora involucrata* Hk.f. |  | 2100–3500 | H | Wp |  | Medicinal (Cough, cold) | 0.20 | 0.30 | | 0.30 | | 0.0050 | | 0.33 | |  |
|  | **Balsaminaceae** |  |  |  |  |  |  |  |  | |  | |  | |  | |  |
| 150 | *Impatiens balsamina* L. | Nanteela | 1200-1900 | H | Wp |  | Medicinal (Inflammation, bums, scalds, ulcers, constipation, arthritis, urinary retention); Fodder | 0.80 | 0.60 | | 0.20 | | 0.0133 | | 0.67 | |  |
| 151 | *Impatiens glandulifera* Royle | Mewa | 2500-3600 | H | Fl, Sd |  | Medicinal (Cooling, tonic); dye | 0.30 | 0.30 | | 0.06 | | 0.0020 | | 0.42 | |  |
| 152 | *Impatiens scabrida* DC. |  | 700-1500 | H | Sd, Wp |  | Medicinal (Abortion); Fodder | 0.20 | 0.30 | | 0.04 | | 0.0013 | | 0.42 | |  |
|  | **Begoniaceae** |  |  |  |  |  |  |  |  | |  | |  | |  | |  |
| 153 | *Begonia picta* Sm. | Khattu | 600–2800 | H | Wp, Lf |  | Medicinal (Body pain, heat stroke, dysentery, mouth ulcers) | 0.40 | 0.60 | | 0.60 | | 0.0200 | | 0.58 | |  |
|  | **Berberidaceae** |  |  |  |  |  |  |  |  | |  | |  | |  | |  |
| 154 | *Berberis jaeschkeana* Sch. | Kashamal | 3000–3500 | S | Rt, Fr, Wd |  | Medicinal (Astringent, blood purifier, diuretic, eye disease, jaundice, menorrhoea, skin disease, eye trouble, ever, stomach disorders, blood purifier, astringent, diuretic) | 1.30 | 0.60 | | 0.60 | | 0.0200 | | 0.58 | |  |
| 155 | *Berberis kashmiriana* Ahrendt |  | 2000–3300 | S | Rt |  | Medicinal (Fever) | 0.10 | 0.30 | | 0.30 | | 0.0050 | | 0.33 | |  |
| 156 | *Berberis pseudumbellata* Parker |  | 2000–2800 | S | Rt, Lf |  | Medicinal (Intestinal disorder) | 0.10 | 0.30 | | 0.30 | | 0.0050 | | 0.33 | |  |
| 157 | *Berberis asiatica* Roxb. ex DC. | Kashmal | 600-2600 | S | Fr, Rt, Bk, St | Least Concern | Medicinal (Antidote to snake bite, boils, eye complaints, kidney stones, jaundice, malaria, piles); Edible | 0.80 | 0.90 | | 0.30 | | 0.0300 | | 0.92 | |  |
| 158 | *Berberis chitria* Edward | Kashamal | 2000-3000 | S | Fr |  | Medicinal (Snake bite, boils, eye complaints, malaria, piles); Edible | 0.60 | 0.30 | | 0.06 | | 0.0020 | | 0.42 | |  |
| 159 | *Berberis lycium* Royle | Kashmal/ Daruhaldi | 1600-3000 | S | Rt, St, Bk |  | Medicinal (Jaundice, eye diseases, dysentery, malaria, stomach diseases); Edible; Fuel | 0.70 | 1.20 | | 0.06 | | 0.0160 | | 1.33 | |  |
| 160 | *Berberis petiolaris* Wall. ex G. Don |  | 1800–2700 | S | Rt |  | Medicinal (Eye & skin complaints) | 0.10 | 0.30 | | 0.30 | | 0.0050 | | 0.33 | |  |
| 161 | *Sinopodophyllum hexandrum* (Royle) T.S.Ying | Ban Kakri | 2400-4500 | H | Fr, Rt, Sd |  | Medicinal (Asthma, joint pain, prevent bloating in cattles) | 0.30 | 0.60 | | 0.60 | | 0.0200 | | 0.58 | |  |
|  | **Betulaceae** |  |  |  |  |  |  |  |  | |  | |  | |  | |  |
| 162 | *Alnus nitida* (Spach) Endl. | Kosh cones | 1000-2700 | T | Bk | Least Concern | Medicinal (Antipoisonous); fodder | 0.20 | 0.60 | | 0.08 | | 0.0053 | | 0.67 | |  |
| 163 | *Betula alnoides* Buch.-Ham.ex D.Don | Shoed | 1500-2500 | T | Bk, Lf | Least Concern | Medicinal (Snake antidote) | 0.10 | 0.30 | | 0.30 | | 0.0050 | | 0.33 | |  |
| 164 | *Betula utilis* D.Don | Bhoj patra/Birch | 2700-4300 | T | St, Bk | Least Concern | Medicinal (Urinary infection, antiseptic, fever, cuts, ear complaints, hysteria, jaundice, wounds, burns, contraceptive, veterinary ailments); thatching roofs | 1.20 | 1.20 | | 0.35 | | 0.0467 | | 1.17 | |  |
| 165 | *Carpinus viminea* Wall.ex Lindl. | Mandu | 1800-2700 | T | Lf , Bk | Least Concern | Fodder; making shoes | 0.20 | 0.30 | | 0.06 | | 0.0020 | | 0.42 | |  |
| 166 | *Corylus jacquemontii* Decne. | Jamun | 2400–2800 | T | Fr, Lf | Data deficient | Medicinal (Fever) | 0.10 | 0.60 | | 0.01 | | 0.0006 | | 0.67 | |  |
|  | **Bignoniaceae** |  |  |  |  |  |  |  |  | |  | |  | |  | |  |
| 167 | *Oroxylum indicum* (L.) Kurz | Shyonak, Tatpalanga | Upto 1200 | T | Lf , Rt, Bk, Sd |  | Medicinal (Diarrhoea, dysentery, rheumatism, fruits are refreshing, purgative, enlarged spleen) | 0.70 | 0.30 | | 0.30 | | 0.0050 | | 0.33 | |  |
| 168 | *Tecoma stans* (L.) Juss. ex Kunth |  | 800-1600 | T | Wp | Least Concern | Medicinal (Diabetes, stomach pains, diuretic, syphilis) | 0.20 | 0.30 | | 0.30 | | 0.0050 | | 0.33 | |  |
|  | **Boraginaceae** |  |  |  |  |  |  |  |  | |  | |  | |  | |  |
| 169 | *Arnebia benthamii* (Wall. ex G. Don) Johnston |  | 2800-4300 | H | Rf, Sd |  | Medicinal (Antiseptic, boils, cuts, wounds, hair tonic, fungal hair infection) | 0.60 | 0.60 | | 0.06 | | 0.0040 | | 0.67 | |  |
| 170 | *Arnebia euchroma* (Royle) I. M. Johnst. | Ratanjot, Khamed, Khamet | 3300-4300 | H | Rt |  | Medicinal (Antiseptic, boils, cuts, wounds, hair tonic, fungal hair infection) | 0.60 | 0.30 | | 0.30 | | 0.0050 | | 0.33 | |  |
| 171 | *Bombax ceiba* L. | Simbal | 700-1700 | T | Rt, Bk, Lf |  | Medicinal (Acne, pimples, anaemia, antifertility, aphrodisiac, boils, bone fracture, chicken pox, cholera, cough, diarrohea, fever, gum problem, inflammation, skin diseases, leprosy, tonic, urine complaint) | 1.80 | 0.30 | | 0.30 | | 0.0050 | | 0.33 | |  |
| 172 | *Cynoglossum zeylanicum* Thunb. ex Lehm. |  | 700-2000 | H | Lf, Rt |  | Medicinal (Asthma, cough, vomiting) | 0.30 | 0.30 | | 0.30 | | 0.0050 | | 0.33 | |  |
| 173 | *Ehretia acuminata* R.Br. | Bakli/Bakaar/Banchaula | 700-1100 | T | Bk, Fr, Wd | Least Concern | Medicinal (Healing, sores on tongue); Edible; Agricultural Tools | 0.40 | 0.60 | | 0.12 | | 0.0040 | | 0.58 | |  |
| 174 | *Ehretia laevis* Roxb. |  | 700-1000 | T | Lf, Bk, Fr |  | Medicinal (Mussel pain); Edible | 0.20 | 0.30 | | 0.30 | | 0.0050 | | 0.33 | |  |
| 175 | *Eritrichium canum* (Benth.) Kitam. |  | 3500–4600 | H | Wp |  | Medicinal (Facilitates children birth) | 0.10 | 0.30 | | 0.30 | | 0.0050 | | 0.33 | |  |
| 176 | *Lindelofia longiflora* (Benth.) Baill. | Showarag, Showara | 3200–4600 | H | Lf |  | Medicinal (Diarrohea, inflammation) | 0.00 | 0.60 | | 0.09 | | 0.0030 | | 0.58 | |  |
| 177 | *Microula tibetica* Benth. |  | 5600 | H | Wp |  | Medicinal (Cough, pulmonary disorder) | 0.20 | 0.30 | | 0.30 | | 0.0050 | | 0.33 | |  |
| 178 | *Onosma hispida* Wall. ex G. Don | Ratanjot | 3000-3800 | H | Rt |  | Medicinal (Hair fall) | 0.10 | 0.30 | | 0.03 | | 0.0010 | | 0.42 | |  |
|  | **Brassicaceae** |  |  |  |  |  |  |  |  | |  | |  | |  | |  |
| 179 | *Capsella bursa-pastoris*  (L.) Medik |  | 700-2000 | H | Wp |  | Medicinal (Blood pressure, dropsy) | 0.20 | 0.30 | | 0.30 | | 0.0050 | | 0.33 | |  |
| 180 | *Cardamine impatiens* L. |  | 700-2000 | H | St |  | Medicinal (Tonic); Edible | 0.20 | 0.30 | | 0.04 | | 0.0013 | | 0.42 | |  |
| 181 | *Coronopus didymus* L. | Murchadi | 1500-3200 | H | Lf , Sd |  | Medicinal (Cold, cough, headache, bronchitis, pneumonia, fungal infections, blood thinner ) | 0.70 | 0.30 | | 0.30 | | 0.0050 | | 0.33 | |  |
| 182 | *Lepicfium verginicum* L. |  | 700-1300 | H | Wp |  | Medicinal (Diuretic and stimulant, asthma, cough,bleeding piles) | 0.50 | 0.30 | | 0.30 | | 0.0050 | | 0.33 | |  |
|  | **Buddlejaceae** |  |  |  |  |  |  |  |  | |  | |  | |  | |  |
| 183 | *Buddleja asiatica* Lour. |  | 700-1000 | Sh | Lf | Least Concern | Medicinal (Inflammation); Edible | 0.20 | 0.30 | | 0.06 | | 0.0020 | | 0.42 | |  |
| 184 | *Buddleja crispa* Benth. | Sfed saryu | 1400-1900 | Sh | Lf, Wd |  | Fodder; Fuel | 0.20 | 0.30 | | 0.06 | | 0.0020 | | 0.42 | |  |
|  | **Buxaceae** |  |  |  |  |  |  |  |  | |  | |  | |  | |  |
| 185 | *Buxus wallichiana* Baillon |  | 1800–2700 | T | Lf , Bk, Wd |  | Medicinal (Combs, purgative, diaphoretic, rheumatic, syphilis) | 0.50 | 0.30 | | 0.09 | | 0.0030 | | 0.42 | |  |
| 186 | *Sarcococca saligna (*D. Don) Müll. Arg. | Rethali | 1500-2800 | S | St |  | Used for making brooms. | 0.10 | 0.30 | | 0.04 | | 0.0013 | | 0.42 | |  |
|  | **Cactaceae** |  |  |  |  |  |  |  |  | |  | |  | |  | |  |
| 187 | *Opuntia monacantha* Haw. |  | 700-1400 | Sh | St, Fr | Least Concern | Medicinal (Hypotensive, anti-inflammatory, antihyperglycaemic, whooping coughs) | 0.40 | 0.30 | | 0.30 | | 0.0050 | | 0.33 | |  |
|  | **Caesalpiniaceae** |  |  |  |  |  |  |  |  | |  | |  | |  | |  |
| 188 | *Bauhinia racemosa* Lam. |  | 700-1000 | T | Lf, Bk, Sd, Fr |  | Medicinal (Diarrohea, dysentery, cholera, snake bite); Edible; Fuel; Fodder | 0.70 | 0.30 | | 0.02 | | 0.0016 | | 0.58 | |  |
| 189 | *Bauhinia retusa* Roxb. |  | 700-1000 | T | Gum, Bud |  | Medicinal (cholera, snake bite); Fuel; Fodder | 0.40 | 0.30 | | 0.12 | | 0.0060 | | 0.50 | |  |
| 190 | *Bauhinia vahlii* Wight &Arn. | Torre | 200-1300 | S | Lf , Fr, Sd, Rt |  | Medicinal (Antifertility, dysentery, fatness, stomachache, tonic); Edible; Fuel; Fodder, Religious | 0.70 | 0.60 | | 0.03 | | 0.0053 | | 0.92 | |  |
| 191 | *Bauhinia variegata* L. | Kachnar | Upto 1900 | T | Lf | Least Concern | Medicinal (Diarrohea, dysentery, fatness, flatulence, piles, scrofula, skin disease, Leprosy, snakebite, tumors, ulcers, worms); Edible; Fuel; Fodder; Religious | 1.60 | 0.60 | | 0.04 | | 0.0056 | | 0.83 | |  |
| 192 | *Caesalpinia bonduc* (L.) Roxb. |  | 700-1200 | Sh | Rt, Bk, Sd | Least Concern | Medicinal (anthelmintic, diuretic, anti-pyretic, febrifuge) | 0.40 | 0.30 | | 0.30 | | 0.0050 | | 0.33 | |  |
| 193 | *Caesalpinia occidentalis* L. |  | 700-1200 | Sh | Rt, Lf, Fl, Sd |  | Medicinal (Diuretic, fevers, asthma, bronchitis, menstrual problems, tuberculosis, anemia, liver complaints, tonic for general weakness and illness, gonorrhea, urinary tract disorders, edema, stomach colic, anti-inflammatory, expel intestinal worms and parasites, skin disorders, wounds, skin fungus, parasitic skin diseases, abscesses) | 2.00 | 0.30 | | 0.03 | | 0.0005 | | 0.33 | |  |
| 194 | *Caesalpinia tora* L. |  | 700-1000 | H | Sd, Lf |  | Medicinal (Antispasmodic, carminative, anti-cholesterol, emollient, ophthalmic, purgative, indigestion, stomachache complaints, eczema and skin conditions) | 1.00 | 0.30 | | 0.30 | | 0.0050 | | 0.33 | |  |
| 195 | *Caesalpinia mimosoides* L. |  | 700-1100 | H | Rt, Lf |  | Medicinal (Constipation, cold, fever, skin disorders, intestinal disorders) | 0.50 | 0.30 | | 0.30 | | 0.0050 | | 0.33 | |  |
| 196 | *Cassia fistula* L. | Amaltas | Upto 1500 | T | Lf , Fr, Sd | Least Concern | Medicinal (Constipation, fever, common cold,leucodenna, swellings, pain, liver disorders, tuberculosis, sore eyes, diabetes, purification ofblood, haematuria, typhoid, asthma, leprosy) | 1.50 | 0.60 | | 0.06 | | 0.0020 | | 0.58 | |  |
|  | **Campanulaceae** |  |  |  |  |  |  |  |  | |  | |  | |  | |  |
| 197 | *Codonopsis affinis* Hk.f. & Th. |  | 2500–3500 | H | Rt |  | Medicinal (Rheumatism, swollen joints, bruises) | 0.30 | 0.30 | | 0.30 | | 0.0050 | | 0.33 | |  |
| 198 | *Codonopsis ovata* Benth. |  | 3000–4200 | H | Rt, Sd, Fl |  | Medicinal (Swollen joints, bruises) | 0.20 | 0.30 | | 0.30 | | 0.0050 | | 0.33 | |  |
|  | **Cannabinaceae** |  |  |  |  |  |  |  |  | |  | |  | |  | |  |
| 199 | *Cannabis sativa* L | Bhang | Upto 3000 | H | Lf |  | Medicinal (Anthelmentic, appetizer, bowel complaints, cold, cough, convulsions, cramps, epilepsy, cuts, dyspepsia, ear complaints, eye diseases, laxative, narcotic, nervine stimulant, piles, skin diseases, joint pain, piles, fever, tetanus, paralysis) | 2.20 | 1.20 | | 0.32 | | 0.0427 | | 1.17 | |  |
|  | **Capparaceae** |  |  |  |  |  |  |  |  | |  | |  | |  | |  |
| 200 | *Capparis zeykmica* L. |  | 700-1000 | Sh | Wp |  | Medicinal (Antihelmintic, blisters, boils, cholera, colic, pneumonia, piles, rheumatism, snake- bite, swell testicle, ulcer); Edible | 1.20 | 0.30 | | 0.30 | | 0.0050 | | 0.33 | |  |
|  | **Caprifoliaceae** |  |  |  |  |  |  |  |  | |  | |  | |  | |  |
| 201 | *Dipsacus inermis Wall* |  | 2200-3250 | H | Lf |  | Medicinal (Acne) | 0.10 | 0.30 | | 0.30 | | 0.0050 | | 0.33 | |  |
| 202 | *Lonicera angustifolia* Wall. ex DC. |  | 2700–3600 | S | Fr |  | Medicinal (Gastric troubles) | 0.10 | 0.30 | | 0.30 | | 0.0050 | | 0.33 | |  |
| 203 | *Morina coulteriana* Royle |  | 3000–3600 | H | Rt |  | Medicinal (Abscesses) | 0.10 | 0.30 | | 0.30 | | 0.0050 | | 0.33 | |  |
| 204 | *Morina longifolia* Wall. Ex DC. |  | 3200–3800 | H | Wp |  | Medicinal (Boils, wounds, burns) | 0.30 | 0.60 | | 0.60 | | 0.0200 | | 0.58 | |  |
| 205 | *Valeriana hardwickii* Wall. | Nihani, Mushakbala | 2500-3300 | H | Rt |  | Medicinal (Antidote to poisonous stings of insects, scorpion, epilepsy, hysteria, neurosis, skin disease, insecticides, mental disorder) | 0.80 | 0.60 | | 0.60 | | 0.0200 | | 0.58 | |  |
| 206 | *Valeriana jatamansi* Jones | Nihani | 2500-3300 | H | Wp, Rt |  | Medicinal (Hysteria, urine complaints, hair oil tonic, antidote to sting of insects, hysteria, neurosis, skin disease); Incense; Religious | 0.90 | 0.90 | | 0.03 | | 0.0064 | | 1.08 | |  |
| 207 | *Valeriana wallichii* DC. | Tagar | 1500-3300 | H | Wp |  | Medicinal (Fever) | 0.10 | 0.30 | | 0.30 | | 0.0050 | | 0.33 | |  |
| 208 | *Viburnum cotinifolium* D.Don | Jungli dakh | 1800–3600 | S | Lf, Fr, Bk |  | Medicinal (Menorrhea, Hepatic & digestive disorder) | 0.20 | 0.60 | | 0.60 | | 0.0200 | | 0.58 | |  |
| 209 | *Viburnum grandiflorum* Wallich ex. DC |  |  | S |  |  | Fodder; Edible | 0.10 | 0.30 | | 0.04 | | 0.0013 | | 0.42 | |  |
|  | **Caryophyllaceae** |  |  |  |  |  |  |  |  | |  | |  | |  | |  |
| 210 | *Cerastium cerastioides* (L.) Britt. |  | 1500–4700 | H | Wp |  | Medicinal (Backache, body ache, headache, renal pain, cough) | 0.30 | 0.30 | | 0.30 | | 0.0050 | | 0.33 | |  |
| 211 | *Drymaria cordata* (L) Willd.ex Roem. & Schult |  | 700-2000 | H | Wp |  | Medicinal (Asthma, bums, diarrohea, dysentery, fever, skin diseases) | 0.60 | 0.30 | | 0.20 | | 0.0033 | | 0.33 | |  |
| 212 | *Gypsophila cerastioides* D. Don |  | 700-2000 | H | Wp |  | Medicinal (Bodyache, headache, renal pain, cough) | 0.40 | 0.30 | | 0.20 | | 0.0033 | | 0.33 | |  |
| 213 | *Stellaria media* (L.) Villars | Khukawa | 1000-2700 | H | Wp |  | Medicinal (Skin infections, wounds, bone fracture); Edible | 0.40 | 0.60 | | 0.08 | | 0.0053 | | 0.67 | |  |
|  | **Celastraceae** |  |  |  |  |  |  |  |  | |  | |  | |  | |  |
| 214 | *Chenopodium ambrosioides* L. |  | 300-2600 | H | Ap , Lf , Rt |  | Medicinal (Intestinal parasites, infusion, carminative, diaphoretic, emmenagogue, cough, pulmonary obstruction, amenorrhoea, vermifuge, analgesic, narcotic, anaemia, nervineantirheumatic, antitumour, amoebicidal, antiasthmatic, stimulate, diuretic, lactogogue, anthelmintic); Insecticide | 2.10 | 0.30 | | 0.09 | | 0.0030 | | 0.42 | |  |
| 215 | *Dysphania botrys* (L.) Mosyakin & Clemants | Bathu, Sokana | 1500-2500 | H | Lf , Lf |  | Medicinal (Gastric disorder, diuretic, laxative, anthelmintic, diuretic, headache, laxative, liver complaints, stomachache); Edible | 1.00 | 0.60 | | 0.24 | | 0.0160 | | 0.67 | |  |
| 216 | *Euonymus pendulus* Wall. |  | 1400-2600 | T | Rt, Bk, Lf, Ap |  | Medicinal (Dysentery, headache, eye complaints, constipationeye diseases) | 0.40 | 0.30 | | 0.30 | | 0.0050 | | 0.33 | |  |
| 217 | *Euonymus tingens* Wall | Chopru / Jamana | 1700-2200 | T | Rt, Bk, Lf |  | Medicinal (Dysentery, eye diseases, headache) | 0.30 | 0.30 | | 0.30 | | 0.0050 | | 0.33 | |  |
|  | **Chenopodiaceae** |  |  |  |  |  |  |  |  | |  | |  | |  | |  |
| 218 | *Chenopodium album* L. | Bathua | 1250-3500 | H | Lf , Sd |  | Medicinal (Skin diseases, urine complaints, intestinal worms,); Edible | 0.40 | 0.90 | | 0.20 | | 0.0200 | | 0.92 | |  |
|  | **Combretaceae** |  |  |  |  |  |  |  |  | |  | |  | |  | |  |
| 219 | *Terminalia arjuna* (Roxb. ex DC.) Wt. & Arn. |  | 1200-1700 | T | Bk |  | Medicinal (Blood pressure and reduces blood cholesterol levels) | 0.20 | 0.30 | | 0.30 | | 0.0050 | | 0.33 | |  |
| 220 | *Terminalia bellirica* (Gaertn.) Roxb. | Bahera | 300-1300 | T | Fr, Bk |  | Medicinal (Coughs, hoarseness, Sore throat, cold, asthma, conjunctivitis, appetizer, flatulence, thirst, piles, worms, astringent, blood pressure, dysentery, headache, eye diseases); Edible | 1.70 | 0.60 | | 0.12 | | 0.0080 | | 0.67 | |  |
| 221 | *Terminalia chebula* Retz. | Harar | 150-1500 | T | Fr |  | Medicinal (Laxative, anorexia, appetite, tonic, astringent, anthelmintic, expectorant, carminative, skin disorders, anemia, piles, stimulant, diarrhoea, leprosy, fever, heart disease, cough, sore throat, high cough, asthma, ulcers, gout, heart burn, vomiting, diarrhea, dysentery, bleeding piles, bladder diseases, mild laxative, antispasmodic and stomachic); Edible | 3.10 | 0.60 | | 0.06 | | 0.0040 | | 0.67 | |  |
|  | **Commelinaceae** |  |  |  |  |  |  |  |  | |  | |  | |  | |  |
| 222 | *Commelina benghalensis* L. | Chura | 200-1000 | H | Lf | Least Concern | Medicinal (Diarrhea, eye problems, fever, scoipionbite, wounds); Edible | 0.60 | 0.60 | | 0.12 | | 0.0080 | | 0.67 | |  |
| 223 | *Commelina paludosa* Bl. |  | 800-2000 | H | WP |  | Medicinal (On insect stings, diuretic, depressant, hypotensive); Edible | 0.50 | 0.60 | | 0.09 | | 0.0060 | | 0.67 | |  |
| 224 | *Cyanotis cristata* (L.)D.Don |  | 1500-2000 | H | WP, Rt | Least Concern | Medicinal (On insect stings, diuretic, depressant, Veterinary diseases | 0.20 | 0.60 | | 0.50 | | 0.0167 | | 0.58 | |  |
| 225 | *Cyanotis vaga* (Lour.) J.A. and J.H. Schult. |  | 900-2000 | H | Ap |  | Edible | 0.10 | 0.30 | | 0.30 | | 0.0050 | | 0.33 | |  |
| 226 | *Jurinea dolomiaea* Boiss. | Dhoop/ Gaggal dhoop | 3000-4300 | H | Rt |  | Medicinal (Antiseptic, colic, fever, laxative, skin eruptions, gout, rheumatism); Incense | 0.80 | 0.60 | | 0.02 | | 0.0026 | | 0.83 | |  |
| 227 | *Saussurea costus* (Falc.) Lipsch. | Kuth | 3000-4000 | H | Rt | Critically Endangered | Medicinal (Joint pains) | 0.10 | 0.30 | | 0.30 | | 0.0050 | | 0.33 | |  |
|  | **Convolvulaceae** |  |  |  |  |  |  |  |  | |  | |  | |  | |  |
| 228 | *Convolvulus arvensis* L. |  | 700-2000 | H | Wp |  | Medicinal (Purgative, burns, bruises); Detergent | 0.20 | 0.30 | | 0.02 | | 0.0007 | | 0.42 | |  |
| 229 | *Evolvulus alsinoides* (L.) L. | Sankhpushpi | 800-1200 |  | WP |  | Medicinal (Asthma, fever, cough, cold, venereal diseases, azoospennia, adenitis, depression, anti-amnesic, antistress, braintonic fever, scorpion sting, stomachache, epilepsy, leukoderma, cuts, ulcers, brain tonic); Religious | 1.90 | 0.60 | | 0.28 | | 0.0187 | | 0.67 | |  |
| 230 | *Ipomoea nil* (L.) Roth | Ghaudan | 700-1500 | H | Wp |  | Medicinal (Anthelmintic, diuretic, laxative, constipation, antifungal, antispasmodic, antitumor, oedema, oliguria, hallucinogenic, parasiticide, anthelmintic, anticholinergic, antifungal, ascariasis, mental disorders); Fodder | 1.70 | 0.30 | | 0.04 | | 0.0013 | | 0.42 | |  |
| 231 | *Ipomoea purpurea* (L.) Roth |  | 700-2000 | H | Ap, Sd |  | Medicinal (Anthelmintic, laxative, diuretic, hallucinogenic, diuretic, laxative, oedema, oliguria, ascariasis, constipation mental disorders.); Fodder | 1.10 | 0.30 | | 0.06 | | 0.0020 | | 0.42 | |  |
| 232 | *Ipomoea quamoclit* L. | Nagarbel | 1100-1500 |  | Lf,Sd,Rt |  | Medicinal (Physical weakness, abnormal behaviour, sinking of voice, cuts and wounds, piles, snakebites, purgative) | 0.70 | 0.30 | | 0.30 | | 0.0050 | | 0.33 | |  |
|  | **Coriariaceae** |  |  |  |  |  |  |  |  | |  | |  | |  | |  |
| 233 | *Coriaria nepalensis* Wall. | Fanai | 800-2000 | T | St, Lf, Fr |  | Medicinal (Emetic, antifungal, antimicrobial, burns); Fodder | 0.50 | 0.60 | | 0.06 | | 0.0040 | | 0.67 | |  |
|  | **Crassulaceae** |  |  |  |  |  |  |  |  | |  | |  | |  | |  |
| 234 | *Bryophyllum pinnatum* (Lam.) Oken | Patharchatt | Upto 1200 | H | Lf |  | Medicinal (Skin infections, kidney stone, dysentery, diarrhea ) | 0.40 | 0.30 | | 0.30 | | 0.0050 | | 0.33 | |  |
| 235 | *Kalanchoe spathulata* DC. | Patharchat | 1400-2200 | H | Wp, Lf |  | Medicinal (Healing for scar, tumors, cholera, wounds, anti-inflamatory) | 0.50 | 0.60 | | 0.50 | | 0.0167 | | 0.58 | |  |
| 236 | *Rhodiola heterodonta* (Hk.f. & Th.) Boiss. | Hari buti | 3000–5000 | H | Wp |  | Medicinal (Sexual potency, stomach ache, intestinal discomfort, Increase physical endurance, work productivity and longevity, treat fatigue, depression, anemia, impotence, gastrointestinal ailments, nervous system disorders) | 1.10 | 0.60 | | 0.60 | | 0.0200 | | 0.58 | |  |
| 237 | *Rosularia rosulata* (Edgew.) H. Ohba |  | 1400-2200 | H | Wp |  | Medicinal (Milk Production, skin disease) | 0.20 | 0.60 | | 0.50 | | 0.0167 | | 0.58 | |  |
| 238 | *Sedum ewersii* Ledeb. | Mousgrass | 3500-4200 | H | Lf , St |  | Medicinal (Headache, pain in lungs, weakness) | 0.30 | 0.30 | | 0.20 | | 0.0033 | | 0.33 | |  |
|  | **Cuccurbitaceae** |  |  |  |  |  |  |  |  | |  | |  | |  | |  |
| 239 | *Cayaponia laciniosa* (L.) C.Jeffrey |  | 800-2000 | H | Fr |  | Medicinal (Burns, snake bite) | 0.20 | 0.30 | | 0.30 | | 0.0050 | | 0.33 | |  |
| 240 | *Coccinia grandis* (L.) Voigt |  | 900-1300 | H | Rt, Lf, Fr |  | Medicinal (Cold, cough, diabetes, headache, filarial, swell, loss of taste, slow pulse, sores, syphilis, throat effect, vomiting) | 1.20 | 0.30 | | 0.30 | | 0.0050 | | 0.33 | |  |
| 241 | *Melothria heterophylla* (Lour.) Cong. | Bankakri | 1200-2000 | H | Rt, Lf , Fr |  | Medicinal (Antifertility, cuts, diabetes, fever, stomachache, dysuria, stimulant, purgative, gonorrhea, spennatorrhoea); Edible; Fodder | 1.20 | 0.30 | | 0.02 | | 0.0012 | | 0.50 | |  |
| 242 | *Solena amplexicaulis* (Lam.) Gandhi |  | 800-2300 | H | Lf, Rt, Sd, Fr |  | Medicinal (Pain killer, snakebite poisoning) | 0.20 | 0.30 | | 0.30 | | 0.0050 | | 0.33 | |  |
| 243 | *Trichosanthes tricuspidata* Lour. |  | 1500-2200 | C | Lf, Rt, Sd, Fr |  | Medicinal (Bums, diarrohea, dysentery, fever, pneumonia, rheumatism, snake bite, vomiting, Constipation, migraine, carbuncle, otitis, rhinitis, inflammations, asthma, epilepsy, skin diseases) | 1.70 | 0.60 | | 0.60 | | 0.0200 | | 0.58 | |  |
|  | **Cupressaceae** |  |  |  |  |  |  |  |  | |  | |  | |  | |  |
| 244 | *Juniperus communis* Linn. | Bethar, Hauber | 3000-4000 | S | WP, St, Fr | Least Concern | Medicinal (Tonic, diuretic, urinary tract, chronic arthritis, gout, rheumatism, stomach disorders, kidney disorder, liver, bladder, heart disease, nervous disorder, pills, dropsy mucous discharge, antibiotic for animal, repel flies) | 1.60 | 0.30 | | 0.05 | | 0.0032 | | 0.58 | |  |
| 245 | *Juniperus indica* L. | Bitaru | 3000-3800 | S | WP, AP | Least Concern | Medicinal (Cough, cold, tonsillitis, headache, malarial, neck pain, blood pressure) | 0.70 | 0.30 | | 0.30 | | 0.0050 | | 0.33 | |  |
| 246 | *Juniperus macropoda* Boiss. | Bether Patta | 3000-4200 | S | Rt, Bk, Wp |  | Medicinal (Aphrodisiac, styptic, asthma, stomatitis, hemicrania, cronic bronchitis, disease of of liver and spleen, gonorrhoea, gleet, leucorrhoea); Spice; Pencil making | 1.20 | 0.30 | | 0.03 | | 0.0028 | | 0.67 | |  |
| 247 | *Juniperus polycarpos* C.Koch |  | 3000–3600 | T | Lf |  | Medicinal (Wound healing, repel flies, nervous disorder) | 0.30 | 0.30 | | 0.04 | | 0.0013 | | 0.42 | |  |
| 248 | *Juniperus recurva* Buch.- Ham. | Bether Patta | 3500-4500 | S | Rf | Least Concern | Medicinal (Kidney trouble); Fuelwood; Pencil making; Incense; flavous and cordials | 0.50 | 0.30 | | 0.01 | | 0.0011 | | 0.75 | |  |
| 249 | *Thuja orientalis* L |  |  | S | Lf | Near Threatened | Medicinal (antifungal) | 0.10 | 0.30 | | 0.30 | | 0.0050 | | 0.33 | |  |
|  | **Cuscutaceae** |  |  |  |  |  |  |  |  | |  | |  | |  | |  |
| 250 | *Cuscuta reflexa* Roxb. | Akash-Bel | 1000-1400 | H | St, Wp |  | Medicinal (Body ache, burns, cuts, nervine weakness, swellings, body part, veterinary, kills lices, jaundice, bilous disorders) | 1.00 | 0.60 | | 0.60 | | 0.0200 | | 0.58 | |  |
|  | **Cyperaceae** |  |  |  |  |  |  |  |  | |  | |  | |  | |  |
| 251 | *Carex breviculmis* R. Br. |  | 700-1100 | H | Ap |  | Fodder | 0.10 | 0.30 | | 0.30 | | 0.0050 | | 0.33 | |  |
| 252 | *Carex cruciata* Wahlenb. | Dastana ghas | 700-2000 | H | Wp |  | Fodder | 0.10 | 0.30 | | 0.30 | | 0.0050 | | 0.33 | |  |
| 253 | *Carex nubigena* Nees |  | 3000-3700 | H | Ap , Rt |  | Insect bite | 0.10 | 0.30 | | 0.30 | | 0.0050 | | 0.33 | |  |
| 254 | *Carex obscura* Nees |  | 3000–4500 | H | Ap |  | Medicinal (Antiviral, Insect bite) | 0.20 | 0.60 | | 0.60 | | 0.0200 | | 0.58 | |  |
| 255 | *Cyperus compressus* L. |  | 700-2000 | H | Wp | Least Concern | Fodder | 0.10 | 0.30 | | 0.30 | | 0.0050 | | 0.33 | |  |
| 256 | *Cyperus scariosus* L. |  | 700-1700 | H | Wp |  | Fodder | 0.10 | 0.30 | | 0.30 | | 0.0050 | | 0.33 | |  |
| 257 | *Fimbristylis dichotoma* (Vahl) Kunth. |  | 700-2000 | H | Wp | Least Concern | Fodder | 0.10 | 0.30 | | 0.30 | | 0.0050 | | 0.33 | |  |
|  | **Datiscaceae** |  |  |  |  |  |  |  |  | |  | |  | |  | |  |
| 258 | *Datisca cannabina* L. |  | 1000–2500 | H | Lf , Rt |  | Medicinal (Diuretic, febrifuge, purgative, rheumatism, sedative) | 0.50 | 0.30 | | 0.30 | | 0.0050 | | 0.33 | |  |
|  | **Dioscoreaceae** |  |  |  |  |  |  |  |  | |  | |  | |  | |  |
| 259 | *Dioscorea bulbifera* L. | Tardi | 800-2000 | H | Tu |  | Medicinal (Abdominalpain, boils, bone fracture, dysentery, piles, jaundice, roundworms, constipation) | 0.80 | 0.60 | | 0.60 | | 0.0200 | | 0.58 | |  |
| 260 | *Dioscorea deltoidea* Wall. ex Griseb. | Singli Mingli/ Kins | 1100-2600 | Climber | Tb |  | Medicinal (Dysentery, piles, fever, gout, digestive problems, oral contraceptive pills, Rheumatism.); Edible | 0.80 | 1.50 | | 0.42 | | 0.0700 | | 1.42 | |  |
|  | Elaeagnaceae |  |  |  |  |  |  |  |  | |  | |  | |  | |  |
| 261 | *Elaeagnus conferta* Roxb. |  | 1500-2800 | S | Fr, AP, Lf, Sd | Least Concern | Medicinal (Sores, ulcer, cough, bronchitis, febrifuge, expectorant, cuts, ulcer, wounds, cough, dandruff of hairs, skin disease); Edible | 1.30 | 0.60 | | 0.30 | | 0.0200 | | 0.67 | |  |
| 262 | *Elaeagnus parvifolia* Wall. ex Royle | Ghyeen, Geai | 1300-3000 | T | Fr, Wp |  | Medicinal (Astringent, diarrohea, pulmonary infections, sores and ulcers); Edible | 0.50 | 0.90 | | 0.20 | | 0.0200 | | 0.92 | |  |
| 263 | *Elaeagnus umbellata* Thunb | Ghaiyin |  | S |  | Least Concern | Fruit edible. | 0.10 | 0.30 | | 0.30 | | 0.0050 | | 0.33 | |  |
| 264 | *Hippophae rhamnoides* L. |  | 2600–3500 | S | Sd, Frs | Least Concern | Medicinal (Aphrodisiac, lung disease) | 0.20 | 0.30 | | 0.30 | | 0.0050 | | 0.33 | |  |
| 265 | *Hippophae salicifolia* D. Don |  | 2600–3500 | T | Bk, Frs |  | Medicinal (Cuts, ulcers, wounds, cough, dandruff, skin disease, rich source of Vitamin- C; fodder; Fuel | 0.90 | 0.60 | | 0.04 | | 0.0048 | | 0.83 | |  |
|  | **Ephedraceae** |  |  |  |  |  |  |  |  | |  | |  | |  | |  |
| 266 | *Ephedra gerardiana* Wall. ex Stapf | Somlata | 3600-5000 | S | Lf , Rt, St |  | Medicinal (Asthma) | 0.10 | 0.30 | | 0.30 | | 0.0050 | | 0.33 | |  |
| 267 | *Ephedra saxatilis* Stapf. |  | 2300–5200 | S | Lf, Rt, St | Least Concern | Medicinal (Asthma) | 0.10 | 0.30 | | 0.30 | | 0.0050 | | 0.33 | |  |
|  | **Ericaceae** |  |  |  |  |  |  |  |  | |  | |  | |  | |  |
| 268 | *Cassiope fastigiata* (Wall.) D.Don | Salu | 3300-4000 | S | Wp |  | Medicinal (Itching) | 0.10 | 0.30 | | 0.30 | | 0.0050 | | 0.33 | |  |
| 269 | *Gaultheria trichophylla* Royle |  | 2000-3500 | H | Lf, Fr |  | Medicinal (Cough and cold) | 0.10 | 0.30 | | 0.30 | | 0.0050 | | 0.33 | |  |
| 270 | *Lyonia ovalifolia* (Wall.) Drude | Baral, Airean, Ehran | 1300-3300 | T | Lf , Sd, Ap,Wp | Least Concern | Medicinal (Boils, pimples, skin diseases, wormifuge, coetaneous, insecticidal, inflammation of the eyes and face) | 0.70 | 0.60 | | 0.70 | | 0.0233 | | 0.58 | |  |
| 271 | *Rhododendron anthopogon* D. Don | Talis patra | 3300-5100 | S | Ap, Lf |  | Medicinal (Aromatic, bronchitis, cold, cough, gonorrhea, stomach ailment, tea preparation, reduce birth pains, delivery, stimulant, bronchitis) | 1.10 | 0.90 | | 0.80 | | 0.0400 | | 0.83 | |  |
| 272 | *Rhododendron arboreum* Sm. | Brash/Burah | 1500-2700 | T | Fl, Lf | Least Concern | Medicinal (Dysentery, fever, headache, rheumatism, wounds, nose bleeding); Edible; Religious; Fuel | 0.90 | 1.20 | | 0.80 | | 0.0533 | | 1.08 | |  |
| 273 | *Rhododendron campanulatum* D. Don | Kashmiri Patta | 2800-4400 | S | Lf , Fl |  | Medicinal (Small pox, itching, boils, cold, cough, headache, rheumatism, sciatica, skin disease, syphilis, tonic) | 1.10 | 1.20 | | 0.70 | | 0.0467 | | 1.08 | |  |
| 274 | *Rhododendron lepidotum* Wall. | Kashmiri Patta | 2700-3800 | S | Rt, Lf , Fl |  | Medicinal (Boils, cold, cough, headache, rheumatism, sciatica, skin disease, syphilis, tonic, fever, blood purifier, fever, pimples, snuff in tonsil, bronchitis) | 1.50 | 0.60 | | 0.60 | | 0.0200 | | 0.58 | |  |
|  | **Euphorbiaceae** |  |  |  |  |  |  |  |  | |  | |  | |  | |  |
| 275 | *Baliospermum solanifolium* (Burm.) Suresh | Danti | 1100-1500 | S | Lf, Sd, Rt | Least Concern | Medicinal (anthelmintic, alexiteric, diuretic, asthma, bronchitis) | 0.50 | 0.30 | | 0.30 | | 0.0050 | | 0.33 | |  |
| 276 | *Euphorbia fusiformis* Don |  | Up to 600 | H | Rt |  | Medicinal (Fever, gout, rheumatism) | 0.30 | 0.30 | | 0.30 | | 0.0050 | | 0.33 | |  |
| 277 | *Euphorbia helioscopia* L. | Dhudhi | 700-1800 | H | Wp |  | Fodder | 0.10 | 0.30 | | 0.20 | | 0.0033 | | 0.33 | |  |
| 278 | *Euphorbia hirta* L. | Dhudia | upto 2000 | H | Exudates, Wp |  | Medicinal (Antidote in snake bite, asthma, boils of mouth, kidney disease, pain in joints, veterinary, bone fracture, abscess, bronchitis infection, burns, colic, cough, cuts, diarrhoea, dysentery, eczema, excess lactation, eye complains, pain, ring worm, scabies, scorpion bite, toothache, vomiting, coryza, hay fever, bowel complaints, worm infestations, kidney stones, sores); Edible | 3.10 | 0.90 | | 0.42 | | 0.0420 | | 0.92 | |  |
|  | **Euphorbiaceae** |  |  |  |  |  |  |  |  | |  | |  | |  | |  |
| 279 | *Euphorbia pilosa* L. |  | 2000-3050 | H | WP |  | Medicinal (Asthma, bronchitis, chest congestion, throat spasms) | 0.40 | 0.30 | | 0.30 | | 0.0050 | | 0.33 | |  |
| 280 | *Euphorbia prolifera* Buch.-Ham. ex D. Don |  | 1000-2000 | H | Wp |  | Fodder | 0.10 | 0.30 | | 0.30 | | 0.0050 | | 0.33 | |  |
| 281 | *Euphorbia royleana* Boiss. | Choi | 1000–1500 | S | AP |  | Medicinal (Eye complaints, skin diseases, bone fracture, Bleeding, burns, cuts, ear complications, skin diseases, wounds) | 0.90 | 0.60 | | 0.50 | | 0.0167 | | 0.58 | |  |
| 282 | *Euphorbia stracheyi* Boiss. |  | 2600-3000 | H | AP, Lt |  | Medicinal (Cold, cough, fever, headache) | 0.40 | 0.30 | | 0.30 | | 0.0050 | | 0.33 | |  |
| 283 | *Euphorbia thomsoniana* Boiss. |  | 3000–4000 | H | Wp |  | Medicinal (Purgative, skin diseases, eruptions) | 0.30 | 0.30 | | 0.30 | | 0.0050 | | 0.33 | |  |
| 284 | *Euphorbia tibetica* Boiss. |  | 3200–4500 | H | Wp |  | Medicinal (Nausea) | 0.10 | 0.30 | | 0.30 | | 0.0050 | | 0.33 | |  |
| 285 | *Falconeria insignis* Royle | Dudhla | 800-1900 | T | La |  | Medicinal (Germicidal) | 0.10 | 0.60 | | 0.60 | | 0.0200 | | 0.58 | |  |
| 286 | *Jatropha curcas* L. | Safed arand | 800-1500 | S | Lf, Sd | Endangered | Medicinal (Constipation, purgative, burns, cancers, chest pain, eczema, herpes, inflammation, pneumonia, rheumatism, syphilis, eczema, scabies, ringworm, gonorrhea) | 1.50 | 0.60 | | 0.60 | | 0.0200 | | 0.58 | |  |
| 287 | *Mallotus philippensis* (Lam.) Muell.Arg. | Kambla | upto 1800 | T | Sd, Fr | Least Concern | Medicinal (Blisters, boils, skin diseases, snake bite, anthelmintic, antioxidants, aphrodisiac, antioxidant, aphrodisiac, scabies, pimples, ringworms, blood purifier, diarrhoea); Fuel; Dye | 1.60 | 0.60 | | 0.14 | | 0.0140 | | 0.75 | |  |
| 288 | *Phyllanthus emblica* L. | Aambla | 700-2000 | T | Fr, Bk |  | Medicinal (Constipation, antioxidant, asthma, bronchitis, dysentery, scurvy, diuretic, coolant, diabetes, cold, burns, hypoglycemic, skin problem, hypotensive, hair tonic); Edible, Religious | 1.70 | 0.60 | | 0.06 | | 0.0060 | | 0.75 | |  |
| 289 | *Phyllanthus fraternus* G.L.Webster |  | 700-1900 | H | Wp |  | Medicinal (Abort cant, allergy, gastric problem) | 0.20 | 0.30 | | 0.20 | | 0.0033 | | 0.33 | |  |
| 290 | *Phyllanthus parvifolius* Buch.-Ham. ex D.Don |  | 800-2000 | H | Lf |  | Fodder | 0.10 | 0.30 | | 0.30 | | 0.0050 | | 0.33 | |  |
| 291 | *Ricinus communis* L. | Arand | Upto 2500 | H | Lf , Sd |  | Medicinal (Anti-inflammatory, anodyne, antidote, emetic, emollient, expectorant, insecticide, laxative, purgative, tonic, vennifirge, abscess, anasarca, arthritis, asthma, boils, burns, cancer, carbuncles, cholera, cold, colic, convulsions, corns, deafness, delirium, dermatitis, dropsy, epilepsy, fever, flu, gout, headache, moles, rheumatism, sores, stomachache, toothaches, tuberculosis, tumors, wounds, aching joints, pain, constipation in cattle, blood purifier, cough, chronic bronchitis, headache, hepatic disease, pneumonia , rheumatism, snuff) | 5.20 | 1.20 | | 0.80 | | 0.0533 | | 1.08 | |  |
|  | **Fabaceae** |  |  |  |  |  |  |  |  | |  | |  | |  | |  |
| 292 | *Abrus precatorius* L. | Rati | 700-1300 | Sh | Wp |  | Medicinal (Body pain, inflammation of gum, constipation) | 0.30 | 0.30 | | 0.30 | | 0.0050 | | 0.33 | |  |
| 293 | *Acacia catechu* (L.f.) Willd. | Khair | 900-1200 | T | Fl, Wd |  | Medicinal (Skin diseases, cough, obesity, toothache, old, mouth ulcer and piles); Fuel; agricultural implements; Fodder | 1.00 | 0.60 | | 0.04 | | 0.0053 | | 0.83 | |  |
| 294 | *Acacia gageana* Craib. | Bagharne | 700-1600 | Sh | Lf, Fl, Sd |  | Medicinal (Dog bites, flatulence, veterinary diseases); Fodder; Fuel | 0.50 | 0.30 | | 0.30 | | 0.0150 | | 0.50 | |  |
| 295 | *Acacia nilotica* (L.) Delile | Kikker | 100-1500 | T | Bk, Lf | Least Concern | Medicinal (Bleeding uterus) | 0.10 | 0.30 | | 0.30 | | 0.0050 | | 0.33 | |  |
| 296 | *Albizia chinensis* (Osbeck) Merr. | Srinh | 700-1300 | T | Wd, Lf |  | Fuel; Fodder | 0.20 | 0.30 | | 0.03 | | 0.0010 | | 0.42 | |  |
| 297 | *Albizia julibrissin* Durazz |  | 700-1500 | T | Wd, Lf |  | Fuel; Fodder | 0.20 | 0.30 | | 0.04 | | 0.0013 | | 0.42 | |  |
| 298 | *Albizia lebbeck* Benth. | Siris | 800-1500 |  | Fl, Sd, Bk, Rt, Lf |  | Medicinal (Boils, diarrohea, dysentery, gonorrhea, eye sores, night blindness, piles, swelling, stress and depression); Misc. (Detergent) | 1.00 | 0.60 | | 0.20 | | 0.0133 | | 0.67 | |  |
| 299 | *Astragalus candoleanus* Royle ex Benth. |  | 3000–4500 | H | Rt |  | Medicinal (Blood purifier, cough, skin disease) | 0.30 | 0.30 | | 0.30 | | 0.0050 | | 0.33 | |  |
| 300 | *Astragalus chlorostachys* Royle ex Benth. |  | 2000-3500 | H | Ap |  | Medicinal (Tonic, febrifuge, tuberculosis) | 0.30 | 0.30 | | 0.20 | | 0.0033 | | 0.33 | |  |
| 301 | *Astragalus himalayanus* Klotz. |  | 3000-4000 | H | Lf, AP,Sd |  | Medicinal (Leprosy) | 0.10 | 0.30 | | 0.30 | | 0.0050 | | 0.33 | |  |
| 302 | *Atylosia mollis* Benth |  | 700-1100 | H | Wp |  | Medicinal (Dropsy, pain in leg, pain swell in pregnancy) | 0.30 | 0.30 | | 0.02 | | 0.0003 | | 0.33 | |  |
| 303 | *Butea monosperma* (Lam.) Taub. | Palash | Upto 1500 | T | Sd |  | Medicinal (Pimples cooling, gum, wounds, eye disease, vermifuge, dysentery, diarrhoea, aphrodisiac, ulser, sour throat, astringent, tonic, snake-bite, insecticid); Edible; Religious; | 1.60 | 0.90 | | 0.18 | | 0.0270 | | 1.00 | |  |
| 304 | *Cicer microphyllum* Benth. |  | 3300-3800 | H | Fr,Lf,Sd,AP |  | Medicinal (Sore & mouth disease treatment in cattle, tongue infection, jaundice) | 0.40 | 0.30 | | 0.30 | | 0.0050 | | 0.33 | |  |
| 305 | *Codariocalyx motorius* (Houtt.) H.Ohashi *Merrill.* |  | 800-1600 | H | Rt, Lf, Fr |  | Medicinal (Rheumatism, wounds) | 0.20 | 0.30 | | 0.30 | | 0.0050 | | 0.33 | |  |
| 306 | *Crotalaria albida* Heyne ex Roth. |  | 800-1800 | H | Rt, Sd | Least Concern | Medicinal (Blood purifier, constipation, purgative) | 0.30 | 0.60 | | 0.60 | | 0.0200 | | 0.58 | |  |
| 307 | *Dalbergia sissoo* Roxb. | Shisam, Say ointi | 700-1300 | T | Lf, Wd |  | Medicinal (Skin ailments, astringent, dysentery, eruptions, stimulant, gonorrhea, menon-hagia, headache, leprosy); Fuel; Fodder; Agricultural tools | 1.20 | 0.30 | | 0.03 | | 0.0021 | | 0.58 | |  |
| 308 | *Desmodium concinnum* DC. |  | 700-2000 | Sh | Wp |  | Fodder; Fuel | 0.20 | 0.30 | | 0.02 | | 0.0007 | | 0.42 | |  |
| 309 | *Desmodium elegans* DC. | Pree/Safed lathi | 1200-3000 | S | Wp | Least Concern | Medicinal (Ulcers, cholera); Fodder | 0.30 | 0.60 | | 0.20 | | 0.0133 | | 0.67 | |  |
| 310 | *Desmodium gangeticum* (L.) DC. | Chagla | 800-2200 | S | Wp |  | Medicinal (Snake venom, asthma, bronchitis, cough, dysentery, eye infection, fever, tonic, vomiting) | 0.90 | 0.30 | | 0.20 | | 0.0033 | | 0.33 | |  |
| 311 | *Desmodium microphyllum* (Thunb.) D.C | Kathi | 1100-2300 | H | Rt | Least Concern | Medicinal (Cholera, urinary ailments); Fodder | 0.30 | 0.30 | | 0.06 | | 0.0020 | | 0.42 | |  |
| 312 | *Desmodium sequax* Wall. |  | 700-1200 | Sh | Lf, Rt |  | Medicinal (Antiseptic, diaphoretic ) | 0.30 | 0.30 | | 0.20 | | 0.0033 | | 0.33 | |  |
| 313 | *Desmodium triquetrum* DC. |  | 700-2000 | H | Wp |  | Medicinal (Abdominal pain, cold, cough, fever, snake bite) | 0.50 | 0.30 | | 0.30 | | 0.0050 | | 0.33 | |  |
| 314 | *Flemingia semialata* Roxb. |  | 800-1700 | S | Rt, Lf |  | Medicinal (Tuberculosis) | 0.10 | 0.30 | | 0.30 | | 0.0050 | | 0.33 | |  |
| 315 | *Flemingia strobilifera* R.Br. |  | 800-2000 | H | Rt, Lf |  | Medicinal (Rheumatic problems) | 0.10 | 0.30 | | 0.30 | | 0.0050 | | 0.33 | |  |
| 316 | *Indigofera atropurpurea* Buch.-Ham. ex Hornem. | Kathi | 700-2000 | Sh | Lf, Wd |  | Fuel; Fodder | 0.20 | 0.30 | | 0.06 | | 0.0020 | | 0.42 | |  |
| 317 | *Indigofera heterantha* Wall. Ex Brand | Kali Kathi | 2000-2800 | S | Lf,St,Br |  | Medicinal (Veterinary, urine problem, diarrhoea, dysentery, cough, wound); Edible | 0.70 | 0.90 | | 0.30 | | 0.0300 | | 0.92 | |  |
| 318 | *Indigofera linifolia* (L.f.) Retz. |  | 700-1100 | H | Wp | Least Concern | Medicinal (Veterinary, wounds, sores); Edible | 0.30 | 0.30 | | 0.06 | | 0.0020 | | 0.42 | |  |
| 319 | *Indigofera pulchella* Roxb. |  | 800–1500 | S | Lf , Rt |  | Medicinal (Cough, epilepsy, mennorhagea, swelling face in labor) | 0.40 | 0.30 | | 0.30 | | 0.0050 | | 0.33 | |  |
| 320 | *Lathyrus aphaca* L. |  | 800-1200 | H | Sd | Least Concern | Medicinal (Leucorrhoea) | 0.10 | 0.60 | | 0.50 | | 0.0167 | | 0.58 | |  |
| 321 | *Lespedeza gerardiana* Graham ex Maxim. |  | 1200-2000 | H | Lf |  | Fodder | 0.10 | 0.30 | | 0.30 | | 0.0050 | | 0.33 | |  |
| 322 | *Lotus corniculatus* L. |  | 2000-3700 | H | Ap |  | Medicinal (Anti-inflammatory) | 0.10 | 0.30 | | 0.30 | | 0.0050 | | 0.33 | |  |
| 323 | *Medicago falcata* L. | Kathoama | 2700-4000 | H | Ap |  | Medicinal (Wounds, injuries) | 0.20 | 0.30 | | 0.30 | | 0.0050 | | 0.33 | |  |
| 324 | *Millettia extensa* Benth. ex Baker |  | 700-1100 | Sh | Wp |  | Medicinal (To kill lice and ticks, spleen dislocation, toothache, kill bedbugs, fever); Fodder | 0.60 | 0.30 | | 0.04 | | 0.0013 | | 0.42 | |  |
| 325 | *Mimosa rubicaulis* Lam. |  | 700-1200 | Sh | Wp |  | Medicinal (Throat infection, measles, gum troub le, hysteria, smallpox); Fodder | 0.60 | 0.30 | | 0.04 | | 0.0013 | | 0.42 | |  |
| 326 | *Mucuna pruriens* (L.)DC. |  | 700-1100 | Sh | Sd |  | Medicinal (Aphrodisiac, snakebite, antidepressant, diuretic, purgative, constipation) | 0.20 | 0.30 | | 0.30 | | 0.0050 | | 0.33 | |  |
| 327 | *Ougeinia oojeinensis* (Roxb.) Hochr. |  | 700-1200 | T | St, Lf |  | Medicinal (Asthma, cholera, dysentery); Edible; Fuel | 0.50 | 0.30 | | 0.01 | | 0.0003 | | 0.50 | |  |
| 328 | *Oxytropis mollis R*oyle ex Benth. |  | 2500–3000 | H | Wp |  | Medicinal (Wounds) | 0.10 | 0.30 | | 0.30 | | 0.0050 | | 0.33 | |  |
| 329 | *Parochetus communis* Buch.-Ham. ex D.Don |  | 1800-2800 | H | Fl | Least Concern | Medicinal (Stomache, intestinal pain due to worm) | 0.20 | 0.30 | | 0.30 | | 0.0050 | | 0.33 | |  |
| 330 | *Pueraria tuberosa* (Willd.) DC. |  | 300–1500 | S | Rt |  | Medicinal (Abdominal pain, antiemetic, asthma, body ache, chest pain, cholera, diarrhoea, fever, lactation, mennorhoea, rheumatism, skin disease, swelling, syphilis, tonic, ulcers, veterinary lactation) | 1.70 | 0.30 | | 0.30 | | 0.0050 | | 0.33 | |  |
| 331 | *Robinia pseudoacacia* L. | Ravinia | 700-200 | T | St, Br, Wd | Least Concern | Medicinal (Toothache, antispasmodic, diuretic, tonic, digestive disorders); Fuel; Fodder; Agriculture tools | 0.80 | 0.30 | | 0.01 | | 0.0005 | | 0.58 | |  |
| 332 | *Sesbania bispinosa* (Jacq.) WF Wight |  | 700-1000 | H | Rt, Sd | Least Concern | Medicinal (Alexiteric, anthelmintic, diuretic, wounds, snakebite, skin diseases) | 0.60 | 0.30 | | 0.30 | | 0.0050 | | 0.33 | |  |
| 333 | *Tadehagi triquetrum* (L.) H.Ohashi |  | 1600-2500 | H | WP | Least Concern | Medicinal (Fever and headache) | 0.10 | 0.30 | | 0.30 | | 0.0050 | | 0.33 | |  |
| 334 | *Tephrosia purpurea* (L.) Pers. |  | 1100-1600 | H | WP |  | Medicinal (Skin disease) | 0.10 | 0.30 | | 0.30 | | 0.0050 | | 0.33 | |  |
| 335 | *Trifolium pratense* L. |  | 2000-3500 | H | AP | Data deficit | Medicinal (Astringent, bronchitis, cough) | 0.30 | 0.30 | | 0.30 | | 0.0050 | | 0.33 | |  |
| 336 | *Trifolium repens* L. |  | 1100-3000 | H | AP |  | Medicinal (Cough, bronchitis, venereal disease, menopause, health drink) | 0.50 | 0.90 | | 0.90 | | 0.0450 | | 0.83 | |  |
| 337 | *Vicia rigidula* Royle |  | 700-1100 | H | Wp |  | Fodder | 0.10 | 0.30 | | 0.30 | | 0.0050 | | 0.33 | |  |
| 338 | *Vigna vexillata* (L.) A.Rich. |  | 2000-2800 | H | Rt, Sd |  | Medicinal (Dysentery, stomache disorder) | 0.20 | 0.60 | | 0.60 | | 0.0200 | | 0.58 | |  |
|  | **Fagaceae** |  |  |  |  |  |  |  |  | |  | |  | |  | |  |
| 339 | *Quercus glauca* Thunb. Bani |  | 1000-2000 | T | Wd, Lf |  | Fodder; Fuel; Timber | 0.30 | 0.30 | | 0.01 | | 0.0006 | | 0.50 | |  |
| 340 | *Quercus leucotrichophora* Camus | Ban | 140-2400 | T | Wd, Sd, Cone |  | Medicinal (Urinary infection, toothache, piles, astringent, diarrhea, stomachache, asthma, dysentery, stomach pain, Astringent and diuretic, gonorrhoea, indigestion, diarrhoea, haemorrhages, chronic diarrhoea) Fodder; Fuel; Timber | 1.70 | 0.60 | | 0.50 | | 0.0167 | | 0.58 | |  |
| 341 | *Quercus semecarpifolia* Sm. | Kharyu | 2500-3100 | T | Lf |  | Medicinal (Wounds) | 0.10 | 0.30 | | 0.30 | | 0.0050 | | 0.33 | |  |
|  | **Flacourtiaceae** |  |  |  |  |  |  |  |  | |  | |  | |  | |  |
| 342 | *Flacourtia indica* (Burm. f.) Merr. |  | 700-1000 | T | Lf, Bk, Fr, Rt | Least Concern | Medicinal (Veterinary foot and mouth disease, bite of medicinal dog, facilitates child birth); Edible | 0.40 | 0.30 | | 0.04 | | 0.0013 | | 0.42 | |  |
| 343 | *Xylosma longifolium* Clos |  | *7*00-1000 | T | Bk, Lf |  | Medicinal (Stomachache); Edible; Fuel | 0.30 | 0.30 | | 0.01 | | 0.0003 | | 0.50 | |  |
|  | **Fumariaceae** |  |  |  |  |  |  |  |  | |  | |  | |  | |  |
| 344 | *Corydalis cornuta* Royle |  | 2400-3500 | H | Rt |  | Medicinal (malarial fever) | 0.10 | 0.30 | | 0.30 | | 0.0050 | | 0.33 | |  |
| 345 | *Corydalis flabellata* Edgew. |  | 3000–4400 | H | Wp |  | Medicinal (Fever) | 0.10 | 0.30 | | 0.30 | | 0.0050 | | 0.33 | |  |
| 346 | *Corydalis govaniana* Wall. | Bhutkeshi | 3300-4000 | H | WP, Rt |  | Medicinal (Antipyretic, diuretic, eye disease, gastric disease, liver, muscle pain, skin disease, syphilis, tonic, leprosy, rheumatism) | 1.10 | 0.60 | | 0.08 | | 0.0027 | | 0.58 | |  |
| 347 | *Corydalis meifolia* Wall. | Tongzil | 3500-4000 | H | WP |  | Medicinal (Headache, liver and rheumatoid problems, leprosy, stomach pain) | 0.40 | 0.30 | | 0.30 | | 0.0050 | | 0.33 | |  |
| 348 | *Fumaria indica* (Hausskn.) Pugsley |  | 700-2000 | H | Wp |  | Medicinal (Diarrohea, fever, liver complaints, skin disease) | 0.40 | 0.60 | | 0.60 | | 0.0200 | | 0.58 | |  |
|  | **Gentianaceae** |  |  |  |  |  |  |  |  | |  | |  | |  | |  |
| 349 | *Gentiana argentea* (D.Don) Griseb. | Pungen karpo | 3000-3800 | H | Lf,Fl |  | Medicinal (Sore throat) | 0.10 | 0.30 | | 0.30 | | 0.0050 | | 0.33 | |  |
| 350 | *Gentiana carinata* (D.Don) Grises |  | 1500–3500 | H | Wp |  | Medicinal (Fever, headache, cold, cough) | 0.40 | 0.60 | | 0.50 | | 0.0167 | | 0.58 | |  |
| 351 | *Gentiana decemfida* Buch.-Ham. ex D.Don |  | 1800-2500 | H | WP |  | Medicinal (Fever, headache) | 0.20 | 0.30 | | 0.30 | | 0.0050 | | 0.33 | |  |
| 352 | *Gentiana kurroo* Royle | Kauri Patties | 1800-4200 | H | Rt | Critically Endangered | Medicinal (Appetite, gastric secretion, stomachic, fever, urinary complaints) | 0.50 | 0.30 | | 0.30 | | 0.0050 | | 0.33 | |  |
| 353 | *Gentianella moorcroftiana* (Wall. ex Griseb.) Airy Shaw | | 2900–5200 | H | Wp |  | Medicinal (Blood purifier, cold, cough, fever, headache, febrifuge, blood purifier, cough, rheumatism, gastric) | 1.00 | 0.60 | | 0.60 | | 0.0200 | | 0.58 | |  |
| 354 | *Gentianella tenella* (Rottb.) Borner |  | 3000–5000 | H | Wp |  | Medicinal (Fever) | 0.10 | 0.30 | | 0.30 | | 0.0050 | | 0.33 | |  |
| 355 | *Gentianopsis detonsa* (Rottb.) |  | 3200–4200 | H | Fl | Least Concern | Medicinal (Cough, fever, headache, nausea) | 0.40 | 0.30 | | 0.30 | | 0.0050 | | 0.33 | |  |
| 356 | *Halenia elliptica* D. Don | Chirata | 2000-4500 | H | Wp |  | Medicinal (Liver, kidney problem) | 0.20 | 0.30 | | 0.30 | | 0.0050 | | 0.33 | |  |
| 357 | *Jaeschkea oligosperma* (Griseb.) Knobl. |  | 2700–4300 | H | Wp |  | Medicinal (Blood purifier, fever, febrifuge) | 0.30 | 0.60 | | 0.60 | | 0.0200 | | 0.58 | |  |
| 358 | *Swertia petiolata* Don |  | 3800–5600 | H | Wp |  | Medicinal (Body ache, headache, gall disorder) | 0.30 | 0.30 | | 0.30 | | 0.0050 | | 0.33 | |  |
| 359 | *Swertia angustifolia* Buch.- Ham. ex D.Don |  | 1600-2400 | H | WP |  | Medicinal (Malaria, fever) | 0.20 | 0.30 | | 0.30 | | 0.0050 | | 0.33 | |  |
| 360 | *Swertia chirata* (Roxb.) Buch.-Ham. ex Wall. | Chariyata | 1500-2500 | H | Wp |  | Medicinal (Antiemetic to pregnant women, asthma, bilious, blood purifier, bronchitis, fever, inflammation, leprosy, scabies, skin diseases, stomach ache, thirst, tonicagainst skin infections) | 1.30 | 0.30 | | 0.30 | | 0.0050 | | 0.33 | |  |
| 361 | *Swertia ciliata* (G.Don) Burtt |  | 2700-4000 | H | Lf |  | Medicinal (fever) | 0.10 | 0.30 | | 0.30 | | 0.0050 | | 0.33 | |  |
| 362 | *Swertia cordata* (Wall. ex G. Don) C.B. Cl | Charaite | 2700-3500 | H | WP |  | Medicinal (Stomache trouble) | 0.10 | 0.30 | | 0.30 | | 0.0050 | | 0.33 | |  |
| 363 | *Swertia paniculata* Wall. | Charaite | 2000-2600 | H | WP |  | Medicinal (fever) | 0.10 | 0.30 | | 0.30 | | 0.0050 | | 0.33 | |  |
| 364 | *Swertia thomsonii* Cl. ex Hk.f & Th. |  | 3000–3800 | H | Wp |  | Medicinal (Fever, headache) | 0.20 | 0.30 | | 0.30 | | 0.0050 | | 0.33 | |  |
|  | **Geraniaceae** |  |  |  |  |  |  |  |  | |  | |  | |  | |  |
| 365 | *Geranium himalayense* Klot. |  | 2500–4200 | H | Rt |  | Medicinal (Bruises, stomach ache) | 0.20 | 0.30 | | 0.30 | | 0.0050 | | 0.33 | |  |
| 366 | *Geranium nepalense* Sw. | Laljari/ Raktjari | 1500-4000 | H | Wp |  | Medicinal (Cuts, jaundice, toothache, ulcer, wounds, stomach complaints, astringent, disease of the kidneys, rheumatic problem); Dye | 1.00 | 0.60 | | 0.20 | | 0.0133 | | 0.67 | |  |
| 367 | *Geranium pratense* L. |  | 2200-3050 | H | WP |  | Medicinal (Fever) | 0.10 | 0.30 | | 0.20 | | 0.0033 | | 0.33 | |  |
| 368 | *Geranium wallichianum* D. Donex Sweet | Ratanjot | 2100–4200 | H | Rt |  | Medicinal (Cure fever, cough, jaundice, body pain, astringent, ear & eye disease, toothache); Dye | 0.80 | 0.30 | | 0.08 | | 0.0027 | | 0.42 | |  |
|  | **Gesneriaceae** |  |  |  |  |  |  |  |  | |  | |  | |  | |  |
| 369 | *Didymocarpus pedicellata* R.Br. |  | 800–1700 | H | Lf |  | Medicinal (Kidney & bladder stones) | 0.10 | 0.30 | | 0.30 | | 0.0050 | | 0.33 | |  |
|  | **Hypericaceae** |  |  |  |  |  |  |  |  | |  | |  | |  | |  |
| 370 | *Hypericum japonicum* Thunb. ex Murr. |  | 1600-2600 | H | WP |  | Medicinal (Quick healing) | 0.10 | 0.30 | | 0.30 | | 0.0050 | | 0.33 | |  |
| 371 | *Hypericum oblongifolium* Choisy | Kharau, Peol | 2000-3000 | S | Rt, Fr, Fl |  | Medicinal (anti-depressant, anti-cancer, anti-tumor and anti-viral, AIDS as a hepatoprotectant, wounds,sores ulcers swellings and rheumatism, boils, wounds) | 0.80 | 0.90 | | 0.06 | | 0.0120 | | 1.08 | |  |
| 372 | *Hypericum patulum* C.P. Thunberg ex A. Murray | Khaarera/ Basant | 1500-2400 | S | Sd |  | Medicinal (antidepressant, antiviral, antimicrobial, anti-inflammatory, and a healing agent) | 0.50 | 0.30 | | 0.30 | | 0.0050 | | 0.33 | |  |
| 373 | *Hypericum perforatum* L. | Khaarera/ Basant | 1100-2200 | H | Wp |  | Medicinal (Antidepressant,cancer, tumors, antiviral, lung ailments, cuts, imunity, malarial, antiviral) | 0.90 | 0.90 | | 0.80 | | 0.0400 | | 0.83 | |  |
| 374 | *Hypericum uralum Buch.* | Tumbhul | 1100-2000 | S | Rt, Lf |  | Medicinal (Food poisoning, abortifacient) Pimples, ringworm, urine complaints, sores | 0.50 | 0.90 | | 0.70 | | 0.0350 | | 0.83 | |  |
|  | **Hypoxidaceae** |  |  |  |  |  |  |  |  | |  | |  | |  | |  |
| 375 | *Curculigo orchioides* Gaertn. |  | 800-2000 | H | Rh |  | Medicinal (Wounds) | 0.10 | 0.30 | | 0.30 | | 0.0050 | | 0.33 | |  |
| 376 | *Hypoxis aurea Lour.* |  | 1600-2000 | H | Wp |  | Medicinal (Swelling) | 0.10 | 0.30 | | 0.30 | | 0.0050 | | 0.33 | |  |
|  | **Iridaceae** |  |  |  |  |  |  |  |  | |  | |  | |  | |  |
| 377 | *Iris germanica* L. | Safed Bach | upto 2000 | H | Rt | Near Threatened | Medicinal (Anti-bacterial, anti-ulcer and anti-inflammatory) | 0.30 | 0.30 | | 0.30 | | 0.0050 | | 0.33 | |  |
| 378 | *Iris kashmiriana* Baker |  | 2800–4200 | H | Bb, Lf |  | Medicinal (Rheumatism, pimples, ringworm, urine complaints sores) | 0.40 | 0.60 | | 0.50 | | 0.0167 | | 0.58 | |  |
| 379 | *Iris nepalensis* Don |  | 1800–4000 | H | Bb |  | Medicinal (Rheumatic pain) | 0.10 | 0.30 | | 0.30 | | 0.0050 | | 0.33 | |  |
|  | **Juglandaceae** |  |  |  |  |  |  |  |  | |  | |  | |  | |  |
| 380 | *Juglans regia* L. | Akhrot/ Khod | 1100-2500 | T | Bk, Lf , Fr | Least Concern | Medicinal (Clean teeth, Antihelminthic, astringent, frost bite, rheumatism, sores of toes, toothache, malarial, tonic, astringent, febrifuge); Edible; Timber; Dye; Misc.(Insecticide) | 1.50 | 1.50 | | 0.07 | | 0.0368 | | 1.75 | |  |
|  | **Lamiaceae** |  |  |  |  |  |  |  |  | |  | |  | |  | |  |
| 381 | *Ajuga brachystemon* Maxim. | Neel kanthi | 800-2500 | H | WP |  | Medicinal (Mouth ulcers, diabeties, killing lice) | 0.30 | 0.30 | | 0.30 | | 0.0050 | | 0.33 | |  |
| 382 | *Ajuga bracteosa* Wall ex Benth | Neelkanthi | 1100-2800 | H | Lf , Wp |  | Medicinal (Ascariasis, mouth ulcer, internal sores, frost bite, rheumatism, sores of toes, toothache) | 0.70 | 0.60 | | 0.60 | | 0.0200 | | 0.58 | |  |
| 383 | *Ajuga integrifolia* Buch.-Ham. | Neelkanthi | 800-2600 | H | Rt |  | Medicinal (Snake bite, fever and acts blood purifier) | 0.20 | 0.60 | | 0.60 | | 0.0200 | | 0.58 | |  |
| 384 | *Ajuga parviflora* Benth. | Neelkanthi, Garprek, Nireha | 600–1500 | H | Lf , Wp |  | Medicinal (Febrifuge, itching, ascariasis, febrifuge, blood purifier, diabetes, skin diseases, mouth ulcers) | 0.80 | 0.90 | | 0.90 | | 0.0450 | | 0.83 | |  |
| 385 | *Anisomeles indica* (L.) Kuntze |  | 980-1500 | H | Lf |  | Medicinal (Urinary problems) | 0.10 | 0.30 | | 0.30 | | 0.0050 | | 0.33 | |  |
| 386 | *Clinopodium umbrosum* Koch. |  | 800-2700 | H | WP |  | Medicinal (Blood purifier) | 0.10 | 0.30 | | 0.30 | | 0.0050 | | 0.33 | |  |
| 387 | *Clinopodium vulgare* L. | Shyul | 2700-3300 | H | Sd |  | Spices; Edible | 0.20 | 0.30 | | 0.06 | | 0.0020 | | 0.42 | |  |
| 388 | *Colebrookea oppositifolia* Sm. | Bindi Phool | 250-1700 | S | Lf , Rt |  | Medicinal (Cough, burns, cuts, eye complaints, hysteria, toothache, sores, wounds) | 0.80 | 0.60 | | 0.60 | | 0.0200 | | 0.58 | |  |
| 389 | *Coleus aromaticus* Benth. | Pathan bail |  | H | Lf |  | Medicinal (Antimicrobial, antioxidant and flavoring potential, culinary purpose) | 0.40 | 0.30 | | 0.04 | | 0.0013 | | 0.42 | |  |
| 390 | *Elsholtzia flava* (Benth.) Benth. | Pothi, Jaunkra | 2000-2800 | S | Lf, Fr, Sd |  | Medicinal (Relieve sciatica, stomachache and painful menustration, blood purifier) | 0.30 | 0.60 | | 0.60 | | 0.0200 | | 0.58 | |  |
| 391 | *Elsholtzia fruticosa* D. Don |  | 2000-2800 | S | Fl |  | Medicinal (Skin disease, diarrhoea, stomachache) | 0.30 | 0.30 | | 0.30 | | 0.0050 | | 0.33 | |  |
| 392 | *Elsholtzia strobilifera* Benth. |  | 1600-2700 | H | Lf, Inf |  | Medicinal (Burns) | 0.10 | 0.30 | | 0.30 | | 0.0050 | | 0.33 | |  |
| 393 | *Eremostachys superba* Royle ex Benth. |  | Up to 1000 | H | Lf |  | Medicinal (Enhanced lactation) | 0.10 | 0.30 | | 0.30 | | 0.0050 | | 0.33 | |  |
| 394 | *Hyssopus officinalis* L. | Juffa | 2600-4000 | H | Fling shoots |  | Medicinal (Stimulant, carminative, nervous disorders, urinary trouble) | 0.40 | 0.30 | | 0.30 | | 0.0050 | | 0.33 | |  |
| 395 | *Isodon coetsa* (Buch - Ham. ex D.Don) Kudo | Chichri | 2000-2500 | H | Lf |  | Medicinal (Gastric complaints) | 0.10 | 0.30 | | 0.30 | | 0.0050 | | 0.33 | |  |
| 396 | *Isodon rugosus* (Wall. ex Benth.) Codd |  | 1500-2600 | S | Lf |  | Medicinal (Cuts and wounds) | 0.10 | 0.30 | | 0.30 | | 0.0050 | | 0.33 | |  |
| 397 | *Isodon lophanthoides* var. graciliflorus (Benth.) H. Hara |  | 2000-2600 | H | Lf |  | Medicinal (Dysentery, stomach disorders) | 0.20 | 0.30 | | 0.30 | | 0.0050 | | 0.33 | |  |
| 398 | *Leucas capitata* Desf. |  | 700-2000 | H | Wp |  | Fodder | 0.10 | 0.30 | | 0.30 | | 0.0050 | | 0.33 | |  |
| 399 | *Leucas lanata* Benth. | Madhaini | 700-1500 | H | St, Wp |  | Medicinal (Cough, cold, stomach worms); Edible; Dye | 0.50 | 0.30 | | 0.01 | | 0.0006 | | 0.50 | |  |
| 400 | *Mentha longifolia* (L.) Huds | Jungli Pudina | 1100-3500 | H | Wp | Least Concern | Medicinal (Antiseptic, stimulant, wounds, carminative) carminative, rheumatic pains, digestive, wound, kill maggots); Flavouring | 0.90 | 1.20 | | 0.16 | | 0.0320 | | 1.25 | |  |
| 401 | *Mentha piperita* L. | Pudina | 1100-1600 | H | Wp |  | Medicinal (Headache, nausea, diarrohea, carminative, vomiting, stomachache, dysentery, flatulence, analgesic and anti-inflammatory, reduce excessive bile secretion, antiseptic, carminative, digestive) | 1.30 | 1.20 | | 0.90 | | 0.0600 | | 1.08 | |  |
| 402 | *Micromeria biflora* (Buch.-Ham. Ex D Don) Benth | Chai buti | 900-4000 | H | Wp |  | Medicinal (Tonic, abortifacient, stimulant, aphordisiac, rheumatism, diabetes, remove worms from the wounds, cure ulcers, fungal infections) | 0.90 | 0.30 | | 0.04 | | 0.0013 | | 0.42 | |  |
| 403 | *Nepeta ciliaris* Benth. |  | 2300-3600 | H | Wp |  | Medicinal (Eye disorders, fever) | 0.20 | 0.30 | | 0.30 | | 0.0050 | | 0.33 | |  |
| 404 | *Nepeta eriostachya* Benth. |  | 2300-4000 | H | Wp |  | Medicinal (Eye complications, diuretic) | 0.20 | 0.60 | | 0.60 | | 0.0200 | | 0.58 | |  |
| 405 | *Nepeta glutinosa* Benth. |  | 3300-4400 | H | Wp |  | Medicinal (Pneumonia, fever) | 0.20 | 0.30 | | 0.30 | | 0.0050 | | 0.33 | |  |
| 406 | *Nepeta govaniana* (Wall. Ex Benth.) Benth. |  | 2600-3800 | H | Wp |  | Medicinal (Colds, influenza, diarrhea, colic, insomnia, mentrual cramps) | 0.60 | 0.30 | | 0.30 | | 0.0050 | | 0.33 | |  |
| 407 | *Nepeta hindostana (B.Heyne ex Roth) Haines* |  | 700-1100 | H | Lf, Fl. Wp |  | Medicinal (Anti-inflammatory, brain tonic, muscularpain, stimulant) | 0.40 | 0.30 | | 0.30 | | 0.0050 | | 0.33 | |  |
| 408 | *Nepeta royleana* R.R Stewart |  | 2400-3600 | H | Wp |  | Medicinal (Cerebral tonic) | 0.10 | 0.30 | | 0.30 | | 0.0050 | | 0.33 | |  |
| 409 | *Nepeta discolor* Royle ex Benth. |  | 2700-3800 | H | Wp |  | Medicinal (Eyes injury & infection, cold, cough) | 0.20 | 0.30 | | 0.30 | | 0.0050 | | 0.33 | |  |
| 410 | *Nepeta royleana* Stew. |  | 2000-2800 | H | Wp |  | Medicinal (Dysentery and other stomach disorders) | 0.20 | 0.30 | | 0.30 | | 0.0050 | | 0.33 | |  |
| 411 | *Ocimum basiiicum* L. Bhabri |  | 700-2000 | H | Lf, Rt, Wp |  | Medicinal (Anthelmintic, cholera, epilepsy, antipyretic, earache, canninative, diaphoretic, expectorant, fever, headache, sores, wounds, snakebite, ringworm); Edible | 1.50 | 0.30 | | 0.04 | | 0.0013 | | 0.42 | |  |
| 412 | *Origanum vulgare* L. | Ban Tulasi | 1700-3600 | H | Wp |  | Medicinal (Cough and cold, antiseptic, bronchitis, colic, diarrhoea, asthma, rheumatic pain, skin diseases, toothache, influenza and gastric problems, baths, healing wounds, hair growth, fever, hysteria, influenza, menstrual complaints, stimulant, tonic); Edible; Religious | 2.10 | 1.20 | | 0.06 | | 0.0149 | | 1.33 | |  |
| 413 | *Phlomoides bracteosa* (Royle ex Benth.) Kamelin & Makhm | | 2000-3300 | H | Fl |  | Medicinal (Inflammation of the eyes) | 0.10 | 0.30 | | 0.20 | | 0.0033 | | 0.33 | |  |
| 414 | *Phlomoides mollis* Spr. |  | 800-1500 | H | Sd |  | Medicinal (Rheumatism, tonic) | 0.20 | 0.60 | | 0.50 | | 0.0167 | | 0.58 | |  |
| 415 | *Plectranthus coesta* Buch.-Ham. ex D.Don | Cherachi | 1000-2300 | H | Lf |  | Medicinal (Gastric complaints) | 0.10 | 0.60 | | 0.60 | | 0.0200 | | 0.58 | |  |
| 416 | *Pogostemon benghalensis* (Burm.f.) | Kuntze | 150-1400 | H | Lf , Rt, Wp |  | Medicinal (Cuts, snake bite, haemorrhages, wounds, skin diseases, fever, Digestive disorders, wounds, cough); Edible | 1.00 | 0.60 | | 0.08 | | 0.0053 | | 0.67 | |  |
| 417 | *Prunella vulgaris* L. | Syangave | 1500-3500 | H | Wp | Least concern | Medicinal (Breathing problem, cerebral complaints, cold, gastric complaints, headache, liver complaints) | 0.30 | 0.30 | | 0.30 | | 0.0050 | | 0.33 | |  |
| 418 | *Rabdosia rugosa* (Wall. ex Benth.) H.Hara |  | 700-2000 | S | Lf, Wp |  | Medicinal (Swellings, vermicide, insecticide, stomach pain, anti inflammatory) | 0.50 | 0.30 | | 0.30 | | 0.0050 | | 0.33 | |  |
| 419 | *Rosmarinus officinalis* L. | Romarsi | 800-1500 | S | Lf |  | Medicinal (Headache, boils, skin disease) | 0.30 | 0.30 | | 0.30 | | 0.0050 | | 0.33 | |  |
| 420 | *Roylea cinerea* (D.Don) Baill. | Kadu, Karnait | 1200-3700 | S | Lf , Rt |  | Medicinal (Blood purifier, fever, pimples, tonsils) fever, Blood purifier, pimples, snuff in tonsils | 0.70 | 1.20 | | 0.80 | | 0.0533 | | 1.08 | |  |
| 421 | *Salvia bisor* Wall. |  | 2200–3200 | H | Sd |  | Medicinal (Astringent, antihelminthic) | 0.20 | 0.30 | | 0.30 | | 0.0050 | | 0.33 | |  |
| 422 | *Salvia lanata* Roxb. | Thooth | 1000–1600 | H | Rt,Sd, Lf |  | Medicinal (Astringent, colic, cold, cough, adulterant, colic, diarrhoea, cold, cough) | 0.90 | 0.90 | | 0.80 | | 0.0400 | | 0.83 | |  |
| 423 | *Salvia moorcroftiana* Wall. ex Benth. | Thuth | 1500-2700 | H | Rt,Sd |  | Medicinal (Emetic, hemorrhoids, colic, dysentery, Astringent, emetic, poultice) | 0.70 | 0.90 | | 0.70 | | 0.0350 | | 0.83 | |  |
| 424 | *Salvia nubicola* Wall. ex Sweet | Thuth | 2500-2700 | H | Lf, Rt |  | Medicinal (Wounds, cold, cough) | 0.30 | 0.60 | | 0.60 | | 0.0200 | | 0.58 | |  |
| 425 | *Scutellaria angulosa* Colebr. |  | 700-1000 | H | Lf |  | Medicinal (Dysentery, vomiting) | 0.20 | 0.30 | | 0.30 | | 0.0050 | | 0.33 | |  |
| 426 | *Scutellaria scandens* Buch.-Ham. ex D.Don |  | 800-2100 | H | Lf, Rt |  | Medicinal (Diarrhea and dysentery) | 0.10 | 0.30 | | 0.30 | | 0.0050 | | 0.33 | |  |
| 427 | *Stachys melissaefolia* Benth. |  | 2000-3200 | H | Fl |  | Bee forage | 0.10 | 0.30 | | 0.30 | | 0.0050 | | 0.33 | |  |
| 428 | *Thymus linearis* Benth. | Banajwain | 1800-4000 | H | Wp |  | Medicinal (Antifungal, antibacterial, pain during childbirth, whooping cough, epilepsy, skin eruption, excessive bile secretion, alopecia, phlegm, spasmodic pain, stomachic, cold, toothache, hookworms, liver compliant, heating effect, pain reliever during childbirth to mother, spices) | 1.80 | 0.60 | | 0.60 | | 0.0200 | | 0.58 | |  |
| 429 | *Thymus serpyllum* L. | Banajwain | 1800-3600 | S | Wp |  | Medicinal (Antifungal) | 0.10 | 0.30 | | 0.02 | | 0.0007 | | 0.42 | |  |
|  | **Lauraceae** |  |  |  |  |  |  |  |  | |  | |  | |  | |  |
| 430 | *Cinnamomum tamala* Nees & Eberm | Tej patra | 400-2000 | T | Lf , Bk |  | Medicinal (Heart and throat complaints, cough and cold, stomach-ach) | 0.30 | 0.90 | | 0.50 | | 0.0250 | | 0.83 | |  |
| 431 | *Leea aspera Edgew.* |  | 700-1100 | H | Rt |  | Medicinal (Skin diseases, ring worm) | 0.20 | 0.30 | | 0.30 | | 0.0050 | | 0.33 | |  |
| 432 | *Litsea glutinosa* (Lour.) Robins. |  | 300–1500 | T | Bk, St, Lf, Fr | Least Concern | Medicinal (Rheumatism, arthritis, muscular pain, heat stroke, diarrohea, ulcers, sores, astringent) Boils, bone facture, cold, cough | 1.10 | 0.60 | | 0.60 | | 0.0200 | | 0.58 | |  |
| 433 | *Neolitsea pallens* (D.Don) Momiy. & H. Hara | Jhlunth | 1800-2800 | T | Lf , Fr |  | Medicinal (Hair tonic); Fodder | 0.20 | 0.60 | | 0.08 | | 0.0053 | | 0.67 | |  |
|  | **Liliaceae** |  |  |  |  |  |  |  |  | |  | |  | |  | |  |
| 434 | *Colchecum luteum* Baker |  | 1000–2700 | H | Rt |  | Medicinal (Carminative, laxative, aphrodisiac, allergy, appetizer, gout, rheumatism, spleen inflammation) | 0.80 | 0.30 | | 0.30 | | 0.0050 | | 0.33 | |  |
| 435 | *Eremurus himalaicus* Baker |  | 2100–3800 | H | Bb |  | Medicinal (Wounds, stomach disorder) | 0.20 | 0.30 | | 0.30 | | 0.0050 | | 0.33 | |  |
| 436 | *Fritillaria oxypetalum* Royle | Lahsunia | 2200-2800 | H | Bb |  |  | 0.00 | 0.30 | | 0.30 | | 0.0050 | | 0.33 | |  |
| 437 | *Fritillaria roylei* Hook. | Ban Lehsun | 3000-4000 | H | Rt |  | Medicinal (Asthma, bronchitis, burns, stomach disease, tonic) | 0.50 | 0.30 | | 0.30 | | 0.0050 | | 0.33 | |  |
| 438 | *Gagea lutea* (L.) Ker Gawl | Butti |  |  | Tb | Least Concern | Spices | 0.10 | 0.30 | | 0.30 | | 0.0050 | | 0.33 | |  |
| 439 | *Gloriosa superba* L. | Kalihari | 600–1500 | H | Rt, Lf | Least Concern | Medicinal (Anthemirtic, laxative, alexiteric, abortifacient, ulcers, leprosy, piles, inflammations, abdominal pains, itching, thirst, antihelminthic, snake bite, fever, gout, scabies) | 1.60 | 0.60 | | 0.40 | | 0.0133 | | 0.58 | |  |
| 440 | *Lilium polyphyllum* Don |  | 2100–3300 | H | Bb, Rt | Critically Endangered | Medicinal (Tonic, aphrodisiac, refrigerant) | 0.00 | 0.30 | | 0.20 | | 0.0033 | | 0.33 | |  |
| 441 | *Paris polyphylla* Sm. | Dudhia bach/ Satva | 1500-3000 | H | Bb, Rt |  | Medicinal (Diarrhoea, fever, diarrhoea, febrifuge, analgesic, antiphlogistic, antispasmodic, antitussive, depurative, narcotic, snake bite, ulcer, antibacterial) | 1.30 | 0.60 | | 0.50 | | 0.0167 | | 0.58 | |  |
| 442 | *Polygonatum multiflorum* (L.) All. |  | 2000–3000 | H | Tb |  | Medicinal (Appetite, nerve tonic, aphrodisiac) | 0.30 | 0.30 | | 0.30 | | 0.0050 | | 0.33 | |  |
| 443 | *Polygonatum cirrhifolium* (Wall.) Royle | Salam-Mishri | 1500-3250 | H | Tu, Rt,Lf |  | Medicinal (Leucorrhoea, aphrodisiac, appetite, blood purifier, fever, cuts, wounds, nervine tonic, menorrhagia, problems in the kidney) | 1.00 | 0.90 | | 0.70 | | 0.0350 | | 0.83 | |  |
| 444 | *Polygonatum multiflorum* L. |  | 3200-3800 | H | Tu, AP |  | Medicinal (Tonic, urino-genital disorders) | 0.20 | 0.60 | | 0.60 | | 0.0200 | | 0.58 | |  |
| 445 | *Polygonatum verticillatum* (L.) All. | Salam misari | 2000-3300 | H | Rh, Tu |  | Medicinal (Aphrodisiac, Appetite, appetite, nervine tonic, tonic, wounds, spermatorrhaea, piles, kidney trouble); Edible | 1.00 | 1.20 | | 0.80 | | 0.0533 | | 1.08 | |  |
|  | **Linaceae** |  |  |  |  |  |  |  |  | |  | |  | |  | |  |
| 446 | *Reinwardtia indica* Dum. | Piyan, Matkhena | 1200-1600 | H | AP |  | Medicinal (Mouth sores, Tongue cleaning); Fodder | 0.30 | 0.90 | | 0.80 | | 0.0400 | | 0.83 | |  |
| 447 | *Scurrula pulverulenta* (Wall.) G.Don | Parand | 200-1400 | S | Lf |  | Medicinal (Digestive disorders) | 0.10 | 0.30 | | 0.40 | | 0.0067 | | 0.33 | |  |
| 448 | *Viscum album L.* | Rhini | 800-2500 | S | WP |  | Medicinal (Boils, abortifacient, antifertility, bodyache, wounds and burns) | 0.50 | 0.60 | | 0.60 | | 0.0200 | | 0.58 | |  |
|  | **Lythraceae** |  |  |  |  |  |  |  |  | |  | |  | |  | |  |
| 449 | *Ammannia baccifera* L. | Dadarbutti | 800-1300 | H | WP | Least concern | Medicinal (Skin rejuvenator) | 0.10 | 0.30 | | 0.30 | | 0.0050 | | 0.33 | |  |
| 450 | *Duabanga sonneratioides* Buch.-Ham. |  | 700-1000 | T | Wd, Bk |  | Fuel | 0.10 | 0.30 | | 0.30 | | 0.0050 | | 0.33 | |  |
| 451 | *Lawsonia inermis* L. | Mehandi | 700-1300 | Sh | Lf, Rt, Fl, Sd |  | Medicinal (Pain, ulcer, edema, hair fall, graying of hair, burning sensation, headache, hepatitis, skin diseases, dysmenon^-^heal, anemia); Fuel | 1.20 | 0.30 | | 0.30 | | 0.0050 | | 0.33 | |  |
| 452 | *Woodfordia fruticosa* (L.) Kurz | Dhatki/Dhai | Upto 1600 | S | Fls | Least concern | Medicinal (Bone fracture, bums, cholera, cough, dropsy, dysentery, fever, hemorrhage, injuries, menon-hea, muscle pain, nausea, night blindness, fever, rheumatism, skin disease, small pox, sores, spleen complaints, sprain, ulcer wounds, veterinary sores, alexiteric, uterine sedative,anthelmintic, thirst, dysentery, leprosy, erysipelas, blood diseases, leucorrhoea, menorrhagia and toothache); Edible | 3.30 | 0.90 | | 0.35 | | 0.0350 | | 0.92 | |  |
|  | **Malvaceae** |  |  |  |  |  |  |  |  | |  | |  | |  | |  |
| 453 | *Abelmoschus manihot* (L.) Medikus |  | 1100-1500 | H | Bk, Fl, Lf |  | Medicinal (Intestinal problems) | 0.10 | 0.30 | | 0.30 | | 0.0050 | | 0.33 | |  |
| 454 | *Abutilon indicum* (L.) Sweet | Atibala | 800-1100 | H | Fr, Sd, Bk,Lf,Rt |  | Medicinal (Headaches, gonorrhea, bladder infection, pile) | 0.40 | 0.30 | | 0.30 | | 0.0050 | | 0.33 | |  |
| 455 | *Althaea officinali*s L. | Khatmi | 900-1300 | H | WP |  | Medicinal (Sprains, aching muscles, insect bites, skin inflammations, splinters) | 0.50 | 0.30 | | 0.30 | | 0.0050 | | 0.33 | |  |
| 456 | *Gossypium hirsutum* L. | Kapas |  | H | Lf | Vulnerable | Medicinal (Fertility rate of cattle); Fodder | 0.30 | 0.30 | | 0.30 | | 0.0100 | | 0.42 | |  |
| 457 | *Hibiscus syriacus* L. | Gudhal | 800-1200 | S | Rt,Bk |  | Medicinal (Skin diseases, dizziness, stomachic) | 0.20 | 0.30 | | 0.20 | | 0.0033 | | 0.33 | |  |
| 458 | *Lavatera kashmiriana* Camb. |  | 1800–3600 | H | Rt |  | Medicinal (Urinary irritation, throat problems, mild laxative) | 0.30 | 0.30 | | 0.20 | | 0.0033 | | 0.33 | |  |
| 459 | *Malva neglecta* Wallr. | Sochal |  | H | Lf |  | Medicinal (Malaria, bladder, kidney disorder, laxative, antiobesity); Edible | 0.60 | 0.30 | | 0.04 | | 0.0013 | | 0.42 | |  |
| 460 | *Malva parviflora* L. | Nasochal |  | H | Ap |  | Medicinal (Abortion) | 0.10 | 0.30 | | 0.02 | | 0.0003 | | 0.33 | |  |
| 461 | *Malva verticillata* L. | Chiroti | 1800-2800 | H | WP |  | Medicinal (Cough, emollient pectoral complaints, piles, ulcer, urine complaints, fever) | 0.60 | 0.60 | | 0.50 | | 0.0167 | | 0.58 | |  |
| 462 | *Malvastrum coromandelianum* (L.) Garcke |  | 700-1000 | H | Lf |  | Medicinal (Jaundice, sprain, sores, wounds); Misc. (Broom) | 0.50 | 0.30 | | 0.30 | | 0.0050 | | 0.33 | |  |
| 463 | *Sida cordata* (Burm. f.) Borss. Waalk. | Pharendbuti | 1100-1400 | S | WP |  | Medicinal (Asthma, boils, urine complaints, piles, bone fracture, cuts, wounds, debility, tonic, gonorrhea, dysentery, gastric problem) | 1.20 | 0.90 | | 0.80 | | 0.0400 | | 0.83 | |  |
| 464 | *Urena lobata* L. |  | 700-1700 | Sh | Rt, Lf |  | Medicinal (Abortifacient, cooling, cuts, pain in back, snake) | 0.50 | 0.30 | | 0.30 | | 0.0050 | | 0.33 | |  |
|  | **Martyniaceae** |  |  |  |  |  |  |  |  | |  | |  | |  | |  |
| 465 | *Martynia annua* L. | Bichoo ghas | 900-1200 | H | WP |  | Medicinal (Backache) | 0.10 | 0.30 | | 0.30 | | 0.0050 | | 0.33 | |  |
|  | **Melanthiaceae** |  |  |  |  |  |  |  |  | |  | |  | |  | |  |
| 466 | *Trillidium govanianum* (Wall. ex D.Don) Kunth | Nag Chhatri/Satwa | 2500-3800 | H | Tb |  | Medicinal (Arthritis, dysentery) | 0.20 | 0.60 | | 0.60 | | 0.0200 | | 0.58 | |  |
|  | **Melastomataceae** |  |  |  |  |  |  |  |  | |  | |  | |  | |  |
| 467 | *Osbeckia stellata* Buch.-Ham. Ex Don | Kubsh | 1400-2000 | H | Rt, Lf |  | Medicinal (Cough, digestion, dysentery, nose bleeding, snake bite, wounds, stomachache, toothache) urinary disorders | 0.80 | 0.30 | | 0.30 | | 0.0050 | | 0.33 | |  |
|  | **Meliaceae** |  |  |  |  |  |  |  |  | |  | |  | |  | |  |
| 468 | *Cedrela serrata* Royle | Dari | 1000-2500 | T | Lf , Bk | Least Concern | Medicinal (Antioxidant, antiglycating, antimicrobial) | 0.30 | 0.60 | | 0.60 | | 0.0200 | | 0.58 | |  |
| 469 | *Melia azedarach* L. | Darek | 800-1700 |  | Lf,Fr,Sd | Least Concern | Medicinal (Antipyretic, blood purifier, scabies, measles, skin diseases, wounds, boils, cancer, cholera, diabetes, dysentery, jaundice, heart complaint, leprosy, malaria, piles) | 1.60 | 0.30 | | 0.30 | | 0.0050 | | 0.33 | |  |
| 470 | *Toona ciliata* M.Roem. | Bari phool | Upto 1600 | T | Bk, Fl, Fr | Least Concern | Medicinal (Antiseptic, dysentery, bronchitis, fever, gastric troubles, anthelmiatic, antiseptic, blood purifier, hairfall, headache, , rheumatism, skin disorder, vermifuge,); Fodder; Insectiside; Dye | 1.80 | 0.90 | | 0.02 | | 0.0060 | | 1.17 | |  |
| 471 | *Toona serrata* (Royle) M. Roem. | Daral, Darlein | 1100-2600 | T | St, Br |  | Medicinal (Antiseptic, gastric troubles, antiseptic, dysentery, bronchitis, gastric trouble, appetite) | 0.70 | 0.60 | | 0.50 | | 0.0167 | | 0.58 | |  |
|  | **Menispermaceae** |  |  |  |  |  |  |  |  | |  | |  | |  | |  |
| 472 | *Cissampelos pareira* L. | Jaljamini | 200-2200 | H | Lf |  | Medicinal (Leucorrhea, urinary disorders) | 0.20 | 0.60 | | 0.60 | | 0.0200 | | 0.58 | |  |
| 473 | *Cocculus hirsutus* (L.) W.Theob | Chireta | 1000-1500 | H | Lf& Rt |  | Medicinal (Fever) | 0.10 | 0.30 | | 0.30 | | 0.0050 | | 0.33 | |  |
| 474 | *Stephania glabra* (Roxb.) Miers. | Bis-khapar | 800-1600 | H | Tu |  | Medicinal (Stomach pain) | 0.10 | 0.60 | | 0.50 | | 0.0167 | | 0.58 | |  |
| 475 | *Tinospora cordifolia* (Willd.) Miers | Giloe/ Guduchi | 200-1200 | H | Wp |  | Medicinal (Antidiabetic, antispasmodic, antipyretic, antiallergic, antihyperlypidaemia immunomodulatory, rheumatoid arthritis, diabetes, gout, fever, cancer, upset stomach, peptic ulcer, allergic rhinitis, high cholesterol, hepatitis, syphilis and to strong the immune system , constipation) | 1.70 | 0.60 | | 0.50 | | 0.0167 | | 0.58 | |  |
| 476 | *Tinospora sinensis* (Lour.) Merr. | Bis-khapar | 800-1600 | H | Tu |  | Medicinal (Stomach pain) | 0.10 | 0.60 | | 0.50 | | 0.0167 | | 0.58 | |  |
|  | **Moraceae** |  |  |  |  |  |  |  |  | |  | |  | |  | |  |
| 477 | *Ficus rumphii* Bl. |  | 700-1600 | T | Fr |  | Edible | 0.10 | 0.30 | | 0.30 | | 0.0050 | | 0.33 | |  |
| 478 | *Ficus auriculata* Lour. | Dhura |  | T | Lf | Least concern | Medicinal (Antioxidant activities and prevent cardiovascular, neurodegenerative diseases and cancer, antifungal, anthelmintic and antimicrobial activity); Edible; Fodder | 0.70 | 0.30 | | 0.02 | | 0.0012 | | 0.50 | |  |
| 479 | *Ficus benghalensis* L. | Bad | 700-1100 | T | La, Lf, Fr |  | Medicinal (Blisters, boils, cholera, cough, cuts, diabetes, dysentery, eye complaints, fever, scabies, snake bite, sores in mouth, skin disease, toothache); Edible | 1.50 | 0.30 | | 0.30 | | 0.0050 | | 0.33 | |  |
| 480 | *Ficus hederacea* Roxb. |  | 1300-2000 | Sh | Wd, Lf |  | Fodder; Fuel | 0.20 | 0.30 | | 0.04 | | 0.0013 | | 0.42 | |  |
| 481 | *Ficus nemoralis* Wall. ex Miq. |  | 1500-2000 | T | Fr, Lf, Wd |  | Edible; Fodder, Fuel | 0.30 | 0.30 | | 0.00 | | 0.0002 | | 0.50 | |  |
| 482 | *Ficus palmata* Forsk. | Dhuda, Fegra | 600-2300 | T | Lf , Fr |  | Medicinal (Digestive disorders, cough, boils, constipation, diabetes, dysentery, stomachache); Edible; Fodder | 0.90 | 1.20 | | 0.08 | | 0.0160 | | 1.25 | |  |
| 483 | *Ficus pumila* L. |  | 800-2000 | T | Fr, La |  | Medicinal (Digestive, stomachic) | 0.20 | 0.30 | | 0.30 | | 0.0050 | | 0.33 | |  |
| 484 | *Ficus racemosa* L. | Umreya | 1000-1500 | T | Fr, La | Least concern | Medicinal (Blisters, boils, leprosy, muscle pain, piles, dislocation joints, toothache & moles dysentery, indigestion, laxative and ulcers in cattles constipation); Religious; Fuel; Fodder | 1.30 | 0.60 | | 0.02 | | 0.0027 | | 0.83 | |  |
| 485 | *Ficus religiosa* L. | Peepal | 850 −1400 | T | Lf , Bk |  | Medicinal (Abortificant, cholera, fever, gonorrhea, scabies, asthma, skin disease, smallpox snakebite, sore in mouth, urine problem); Edible; Religious; Fuel; Fodder | 1.40 | 0.90 | | 0.01 | | 0.0036 | | 1.17 | |  |
| 486 | *Ficus roxburghii* Wall. | Traymbalu | 700-1900 | T | Lf, Rt, Wd |  | Edible; Fodder, Fuel | 0.30 | 0.30 | | 0.02 | | 0.0010 | | 0.50 | |  |
| 487 | *Morus alba* L. | Shetoot | 700-1000 | T | Lf, Fr | Least concern | Medicinal (Anthelmintic, Dyspepsia, refrigerant, purgative, vennifuge, sore throat); Edible; Fodder, Fuel; Agricultural Tools; Misc. (Leaves used for rearing silkwonns) | 1.10 | 0.30 | | 0.01 | | 0.0009 | | 0.75 | |  |
| 488 | *Morus serrata* Roxb |  |  | T | Wp |  | Edible; Fodder; agricultural tools; fuel | 0.40 | 0.30 | | 1.00 | | 0.0667 | | 0.58 | |  |
|  | **Morchellaceae** |  |  |  |  |  |  |  |  | |  | |  | |  | |  |
| 489 | *Morchella esculenta* (L.) Pers. | Guchhie | 1800-3600 | Mushroom | Fring Body |  | Medicinal (Cold and cough); Edible | 0.20 | 0.90 | | 0.03 | | 0.0041 | | 1.00 | |  |
|  | **Myricaceae** |  |  |  |  |  |  |  |  | |  | |  | |  | |  |
| 490 | *Myrica esculenta* Buch.-Ham. ex D. Don | Kaphal | 1200-2400 | T | Bk, Fr, Wd |  | Medicinal (Asthma, diarrohoea, cholera, fevers, chronic bronchitis, indigestion, malaria, rheumatism, dysentery, diuresis); Edible; Refreshing drinks; Fuel | 1.30 | 0.60 | | 0.02 | | 0.0032 | | 0.83 | |  |
|  | **Myrsinaceae** |  |  |  |  |  |  |  |  | |  | |  | |  | |  |
| 491 | *Maesa indica* (Roxb.) A. DC. |  | 700-1000 | Sh | Fr |  | Medicinal (Syphilis, women disease); Edible | 0.30 | 0.30 | | 0.02 | | 0.0007 | | 0.42 | |  |
| 492 | *Myrsine africana* Linn. | Kakhum, Shamshad | 300-2700 | S | Fr |  | Medicinal (Anthelmintic, tape worms, laxative andanti aging, blood purifier); Edible; Resin | 0.60 | 0.60 | | 0.04 | | 0.0040 | | 0.75 | |  |
|  | **Myrtaceae** |  |  |  |  |  |  |  |  | |  | |  | |  | |  |
| 493 | *Callistemon citrinus* (Curtis) Skeels |  | upto 1500 | T | Lf |  | Medicinal (Diarrhea, dysentery, rheumatism, cough, bronchitis, antimicrobial, relaxant, cardioprotective, tea substitute) | 0.90 | 0.30 | | 0.06 | | 0.0020 | | 0.42 | |  |
| 494 | *Syzygium cumini* (L.) Skeels | Jamun | 1200-1600 | T | Bk,Fr,Lf |  | Medicinal (Astringent, blister in mouth, diabetes, cancer, piles, pimples, fermentation for rice beer); Edible; Fuel; Religious | 1.00 | 0.60 | | 0.60 | | 0.0200 | | 0.58 | |  |
|  | **Nyctaginaceae** |  |  |  |  |  |  |  |  | |  | |  | |  | |  |
| 495 | *Boerhavia diffusa* L. |  | 700-900 | H | Lf, Rt |  | Medicinal (Abortifacient, anemia, asthma, blood purifier, body heat, bronchitis, child birth, cold, cough, dropsy, dysentery, eczema, epilepsy, eye complaints, liver complaints, tonic, eye complaints, kidney complaints, menstrual complaints, p ain in abdomen, piles, rheumatism, urine complaints, wounds); Edible | 2.50 | 0.30 | | 0.09 | | 0.0030 | | 0.42 | |  |
| 496 | *Mirabilis jalapa* L. | Gulal, Shivkali | 900-2000 | H | Fl |  | Medicinal (Aphrodisiac, purgative, tonic, boils, blisters, child birth, earache, piles); Edible; Religious | 1.00 | 0.30 | | 0.01 | | 0.0004 | | 0.50 | |  |
|  | **Oleaceae** |  |  |  |  |  |  |  |  | |  | |  | |  | |  |
| 497 | *Fraxinus micrantha* L. | Angu | 1500-2500 | T | St,Lf,Br |  | Medicinal (Dysentery) | 0.10 | 0.30 | | 0.30 | | 0.0050 | | 0.33 | |  |
| 498 | *Fraxinus xanthoxyloides* Wall. ex G. Don DC. | Thum | 2000–3000 | T | St, Bk, Above part | Least Concern | Medicinal (Abdominal disorder in animals) | 0.10 | 0.30 | | 0.30 | | 0.0050 | | 0.33 | |  |
| 499 | *Jasminum dispermum* Wall. | Banmalti | 1500-2000 | S | Lf |  | Medicinal (Cuts, wounds, menustral disorders); Fodder | 0.40 | 0.60 | | 0.08 | | 0.0053 | | 0.67 | |  |
| 500 | *Jasminum humile* L. | Peeli chameli | 1500-3000 | S | Br,Rt,Fl |  | Medicinal (Sinus, skin, blood, heart diseases, ringworm, fever) | 0.60 | 1.20 | | 0.80 | | 0.0533 | | 1.08 | |  |
| 501 | *Jasminum mesnyi* Hance |  |  | S | Lf |  | Medicinal (Anti-oxidant, anthelmintic) | 0.20 | 0.30 | | 0.30 | | 0.0050 | | 0.33 | |  |
| 502 | *Jasminum officinale* L. | Juhi | 1800-2500 | S | Fl, Lf, Rt |  | Medicinal (Ringworm, gastric problem); Fodder; Religious | 0.40 | 1.20 | | 0.08 | | 0.0160 | | 1.25 | |  |
| 503 | *Olea ferruginea* Royle | Kahu,Jaitoon | 1100-1500 | T | Rt, LF, Fr |  | Medicinal (Diarrhoea, aphrodisiac, urinary problems) | 0.30 | 0.60 | | 0.60 | | 0.0200 | | 0.58 | |  |
| 504 | *Syringa emodii* Wall. ex Royle |  | 3600-4000 | S | Sd, Fl, Lf |  | Medicinal (Stomach disorder) | 0.10 | 0.30 | | 0.30 | | 0.0050 | | 0.33 | |  |
|  | **Onagraceae** |  |  |  |  |  |  |  |  | |  | |  | |  | |  |
| 505 | *Circaea alpina* L. |  | 2200-3100 | H | WP |  | Medicinal (Healing) | 0.10 | 0.30 | | 0.30 | | 0.0050 | | 0.33 | |  |
| 506 | *Epilobium angustifolium* L. |  | 1800-3200 | H | WP |  | Medicinal (Abdominal pain, hepatic, intestinal, cuts, wounds) | 0.50 | 0.60 | | 0.50 | | 0.0167 | | 0.58 | |  |
| 507 | *Oenothera rosea* L'Hér. ex Aiton |  | 700-2000 | H | Lf |  | Medicinal (Hepatic pain, kidney problems) | 0.20 | 0.30 | | 0.40 | | 0.0067 | | 0.33 | |  |
|  | **Orchidaceae** |  |  |  |  |  |  |  |  | |  | |  | |  | |  |
| 508 | *Calanthe plantaginea* Lindl. |  | 1800-2600 | H | Lf,Bb |  | Medicinal (Healing) | 0.10 | 0.30 | | 0.30 | | 0.0050 | | 0.33 | |  |
| 509 | *Calanthe tricarinata* Lindl. |  | 2000-3300 | H | Lf, Bb |  | Medicinal (Sores, eczema, aphrodisiac) | 0.30 | 0.30 | | 0.30 | | 0.0050 | | 0.33 | |  |
| 510 | *Crepidium acuminatum* (D.Don) Szlach. |  | 1600-2200 |  | Lf, Rt |  | Medicinal (cure bronchitis) | 0.10 | 0.30 | | 0.30 | | 0.0050 | | 0.33 | |  |
| 511 | *Dactylorhiza hatagirea* (D.Don) Soó | Salam Panja/ Hath Panja | 2800-4000 | H | Tb |  | Medicinal (Astringent, bone fracture, expectorant, tonic, wounds, weakness, antibiotic, cough, cold, cuts, sexual disability, rheumatism, blood purifier, expectorant) | 1.40 | 1.50 | | 0.90 | | 0.0750 | | 1.33 | |  |
| 512 | *Epipactis helleborine* (L.) Crantz |  | 3200-3600 | H | Lf, Rh |  | Medicinal (Blood purification, aphrodisiac, fever) | 0.30 | 0.60 | | 0.60 | | 0.0200 | | 0.58 | |  |
| 513 | *Goodyera repens* (L.) R. Br. |  | 2400-2800 | H | AP |  | Medicinal (Appetite; infusion, toothache) | 0.30 | 0.30 | | 0.30 | | 0.0050 | | 0.33 | |  |
| 514 | *Habenaria edgeworthii* Hk.f. ex Collett |  | 2200-3300 | H | Tb |  | Medicinal (Blood purifier, rejuvenator) | 0.20 | 0.30 | | 0.30 | | 0.0050 | | 0.33 | |  |
| 515 | *Habenaria intermedia* D. Don |  | 2000–3000 | H | Tb |  | Medicinal (Tonic) | 0.10 | 0.60 | | 0.08 | | 0.0027 | | 0.58 | |  |
| 516 | *Habenaria marginata* Colebr. |  | 800-1500 | H | Tb |  | Medicinal (Flatulence) urinary problems | 0.10 | 0.90 | | 0.80 | | 0.0400 | | 0.83 | |  |
| 517 | *Habernaria pectinata* (Sm.) D.Don |  | 1500-2000 | H | Lf,Tu |  | Medicinal (Snake bites and arthritis) | 0.10 | 0.30 | | 0.30 | | 0.0050 | | 0.33 | |  |
| 518 | *Herminium lanceum* (Thunb. ex Sw.) Vuijk |  | 1800-3200 | H | AP |  | Medicinal (Urinary diseases) | 0.10 | 1.20 | | 0.90 | | 0.0600 | | 1.08 | |  |
| 519 | *Malaxis muscifera* (Lindl.) Ktze. |  | 2800–4400 | H | Tb | Vulnerable | Medicinal (Tonic for kidney) | 0.10 | 0.60 | | 0.50 | | 0.0167 | | 0.58 | |  |
| 520 | *Platanthera edgeworthii* (Hk.f. ex Collett) |  | 2000-2800 | H | Tu |  | Medicinal (Blood purifier tonic) | 0.10 | 0.30 | | 0.30 | | 0.0050 | | 0.33 | |  |
| 521 | *Rhynchostylis retusa* (L.) Blume | Bhangru/Sukamand | 100-1500 | H | Lf |  | Medicinal (Ear pain, bone fractures) | 0.20 | 0.30 | | 0.30 | | 0.0050 | | 0.33 | |  |
| 522 | *Satyrium nepalense* D.Don |  | 1400-2500 | H | Rh |  | Medicinal (Malaria, dysentery, tonic) | 0.20 | 0.90 | | 0.70 | | 0.0350 | | 0.83 | |  |
| 523 | *Spiranthes sinensis* (Pers.) Ames. |  | 1100-2800 |  | Tu |  | Medicinal (Diarrhea, arresting haemorrhages) | 0.20 | 0.30 | | 0.30 | | 0.0050 | | 0.33 | |  |
|  | **Oxalidaceae** |  |  |  |  |  |  |  |  | |  | |  | |  | |  |
| 524 | *Oxalis corniculata* L. | Ambi/Khati Amli | Upto 3000 | H | WP |  | Medicinal (Blood purifier, treat dyspepsia, appetite, cooling, diarrhoea, epilepsy, eye complaints, fever, digestive, dysentery, scurvy, skin disease, stomachache, swelling, wart); Edible | 1.60 | 1.20 | | 0.25 | | 0.0333 | | 1.17 | |  |
| 525 | *Oxalis corymbosa* DC. |  | 700-1000 | H | Wp |  | Medicinal (Dyspepsia, jaundice) | 0.20 | 0.30 | | 0.30 | | 0.0050 | | 0.33 | |  |
| 526 | *Oxalis latifolia* Kunth | Malori | 1500-2500 | H | WP |  | Medicinal (Cuts, dysentery, fever, insect bite, scurvy, skin disease, stomachache, warts, muscular swellings, boils, pimples); Edible) | 1.20 | 0.60 | | 0.60 | | 0.0200 | | 0.58 | |  |
|  | **Papaveraceae** |  |  |  |  |  |  |  |  | |  | |  | |  | |  |
| 527 | *Argemone mexicana* L. |  | 800-1500 | H | Sd,Rt |  | Medicinal (Emetic, wormicide, leucorrhoea, scorpion, insect bites) | 0.50 | 0.30 | | 0.30 | | 0.0050 | | 0.33 | |  |
| 528 | *Meconopsis aculeata* Royle |  | 3200–3800 | H | Wp |  | Medicinal (Backache, colic, renal pain, tonic) | 0.40 | 0.60 | | 0.50 | | 0.0167 | | 0.58 | |  |
|  | **Parnassiaceae** |  |  |  |  |  |  |  |  | |  | |  | |  | |  |
| 529 | *Parnassia nubicola* Wall. ex Royle |  | 2700-3400 |  | Tu |  | Medicinal (Snake bite) | 0.10 | 0.30 | | 0.30 | | 0.0050 | | 0.33 | |  |
| 530 | *Parnassia pusilla* Hk.f. |  | 2200–4400 | H | Rt |  | Medicinal (Washing burns & other wounds) | 0.20 | 0.30 | | 0.20 | | 0.0033 | | 0.33 | |  |
|  | **Pedaliaceae** |  |  |  |  |  |  |  |  | |  | |  | |  | |  |
| 531 | *Sesamum indicum* L. |  | 900-1800 | H | Rt,Lf,Sd |  | Medicinal (Diuretic, emollient, galactogogue, lenitive and tonic, hair loss, greying, convalescence, chronic dry constipation, dental caries, osteoporosis, stiff joints, dry cough) | 1.20 | 0.30 | | 0.30 | | 0.0050 | | 0.33 | |  |
|  | **Phyllanthaceae** |  |  |  |  |  |  |  |  | |  | |  | |  | |  |
| 532 | *Emblica officinalis* Gaertn. | Amla | upto 1350 | T | Fr |  | Medicinal (Source of vitamin-C, diuretic, laxative, cardiac, astringent and liver tonic, anaemia, diarrhoea and dysentery, dyspepsia, haemorrhage, inflammation of eyes, jaundice, leucorrhoea, menorrhagia and dischare of blood from uterus, blackening of hair) | 1.50 | 0.30 | | 0.20 | | 0.0033 | | 0.33 | |  |
|  | **Phytolaccaceae** |  |  |  |  |  |  |  |  | |  | |  | |  | |  |
| 533 | *Phytolacca acinosa* Roxb. | Kafal, Jharka | 2200-3200 | H | Wp, Lf, Rt |  | Medicinal (Body pain, urinary disordered, stomach cramps, dysentery, wounds, cattle pneumonia); Edible; Local beverages | 0.80 | 1.20 | | 0.04 | | 0.0102 | | 1.33 | |  |
|  | **Pinaceae** |  |  |  |  |  |  |  |  | |  | |  | |  | |  |
| 534 | *Abies pindrow* (Royle ex D.Don) Royle | Talis Patra | 2150-3700 | T | Wd, Lf , Res , Bk |  | Medicinal (Rheumatism, ulcers, asthma, constipation, paurgative, bronchitis, fevers, flatulence, pulmonary and urinary disorders, rheumatism, piles, kidney stones, insomnia, diabetes); Construction; Resin | 1.60 | 0.60 | | 0.09 | | 0.0090 | | 0.75 | |  |
| 535 | *Abies spectabilis* (D.Don) | Talis Patra | 2400-4400 | T | Lf , Res , Bk | Near Threatened | Medicinal (Carminative); Resin | 0.20 | 0.30 | | 0.04 | | 0.0013 | | 0.42 | |  |
| 536 | *Cedrus deodara* (Roxb.) G.Don | Kelu cone/Dyaar | 1500-3000 | T | Cone, Wd, Res , St | Least Concern | Medicinal (Antihelminthic, rheumatism, ulcers, control maggots, skin disease, diarrhea, dysentery,); Fuel; Timber; Resin | 1.10 | 1.50 | | 0.01 | | 0.0043 | | 1.67 | |  |
| 537 | *Pinus gerardiana* Wall ex D.Don. | Chilgaza/Neoza | 1800-3350 | T | Sd, Fr, Rt Bk, Res | Near Threatened | Medicinal (Carminative, stimulant, expectorant); Resin | 0.40 | 0.30 | | 0.04 | | 0.0013 | | 0.42 | |  |
| 538 | *Pinus roxburghii* Sarg. | Chil Cones | 1100-2100 | T | Cone, Needles, Res | Least Concern | Medicinal (Boils, bone fracture, cracks in sole of feet, leprosy, skin diseases, snake bite, sprain, swelling, urine complaints, abscess, dislocation of joints, rheumatic pain, ulcer, unconsciousness); Edible; Fuel; Timber; Agricultural tools; Resin | 1.90 | 0.60 | | 0.01 | | 0.0019 | | 1.00 | |  |
| 539 | *Pinus wallichiana* A.B.Jacks. | Kail cones | 1800-3600 | T | Cone, Res , Bk, Lf | Least Concern | Medicinal (Abscess, dislocation of joints, ulcers, unconsciousness); Fuel; Timber; Resin | 0.70 | 0.60 | | 0.03 | | 0.0043 | | 0.83 | |  |
|  | **Pittosporaceae** |  |  |  |  |  |  |  |  | |  | |  | |  | |  |
| 540 | *Pittosporum eriocarpum* Royle |  | 600–1400 | T | Bk, Rt |  | Medicinal (Bronchitis, expectorant, febrifuge, rheumatism) | 0.40 | 0.60 | | 0.60 | | 0.0200 | | 0.58 | |  |
|  | **Plantaginaceae** |  |  |  |  |  |  |  |  | |  | |  | |  | |  |
| 541 | *Plantago depressa* Willd. | Thram | 2800-3300 | H | WP |  | Medicinal (Cure dysentery, wounds, piles, infant health) | 0.40 | 0.30 | | 0.30 | | 0.0050 | | 0.33 | |  |
| 542 | *Plantago himalaica* Pilg. |  | 1500–3000 | H | Wp |  | Medicinal (Diarrhoea, dysentery, boils) | 0.30 | 0.60 | | 0.60 | | 0.0200 | | 0.58 | |  |
| 543 | *Plantago lanceolata* L. | Isabgol | 1200-1800 | H | Lf |  | Medicinal (Blood purifier, mouth ulcers, constipation) | 0.30 | 0.60 | | 0.60 | | 0.0200 | | 0.58 | |  |
| 544 | *Plantago major* L. | Caratta, Tharma | 1200-2600 | H | Wp | Least Concern | Medicinal (Boils, swelling and pain, constipation, dysentery) | 0.40 | 0.30 | | 0.30 | | 0.0050 | | 0.33 | |  |
| 545 | *Plantago ovata* Forsk | Jangli isbagol | 1100-2000 | H | Sd, Lf |  | Medicinal (Diarrhoea, constipation, diuretic, antiinflamation, dysentery) | 0.50 | 0.60 | | 0.50 | | 0.0167 | | 0.58 | |  |
| 546 | *Wulfeniopsis amherstiana* (Benth.) D.Y. Hong |  | 1500-2700 | H | Rt |  | Medicinal (Stomach disorders, inflammations, wound healing) | 0.30 | 0.30 | | 0.30 | | 0.0050 | | 0.33 | |  |
|  | **Plumbaginaceae** |  |  |  |  |  |  |  |  | |  | |  | |  | |  |
| 547 | *Plumbago zeylanica* L. |  | 700-1200 | H | St, Rt, Fl, La |  | Medicinal (Abortificant, headache, rheumatism) | 0.30 | 0.30 | | 0.30 | | 0.0050 | | 0.33 | |  |
|  | **Poaceae** |  |  |  |  |  |  |  |  | |  | |  | |  | |  |
| 548 | *Apluda mutica* L. | Mushkneti | 1400-2500 | H | WP |  | Medicinal (Mouth sores, constpation); Fodder | 0.30 | 0.60 | | 0.08 | | 0.0053 | | 0.67 | |  |
| 549 | *Arthraxon compositus* (L.) P. Beauv |  | 700-2000 | H | Wp |  | Fodder | 0.10 | 0.30 | | 0.30 | | 0.0050 | | 0.33 | |  |
| 550 | *Arundinaria falcata* Nees | Nargal | 1600-2000 | Sh | Shoots |  | Misc. (Making baskets) | 0.10 | 0.30 | | 0.20 | | 0.0033 | | 0.33 | |  |
| 551 | *Arundinella nepalensis* Trin. | Garein | 800-2000 | H | WP |  | Medicinal (Ointment) | 0.10 | 0.60 | | 0.50 | | 0.0167 | | 0.58 | |  |
| 552 | *Avena fatua* L. | Kasam, Gwajung | 3500-4000 | H | AP |  | Medicinal (Wounds) | 0.10 | 0.30 | | 0.30 | | 0.0050 | | 0.33 | |  |
| 553 | *Bothriochloa intermedia* (R.Br.) A.Camus |  | 700-2000 | H | Wp |  | Fodder | 0.10 | 0.30 | | 0.30 | | 0.0050 | | 0.33 | |  |
| 554 | *Bothriochloa pertusa* (L.) A.Camus |  | 1300-2000 | H | Wp |  | Medicinal (Wounds) | 0.10 | 0.30 | | 0.30 | | 0.0050 | | 0.33 | |  |
| 555 | *Chrysopogon serrulatus* Trin. |  | 700-2000 | H | Lf |  | Fodder | 0.10 | 0.30 | | 0.30 | | 0.0050 | | 0.33 | |  |
| 556 | *Cymbopogon martini* (Roxb.) Wats. | Dantu ghass | 800-1600 | H | Lf |  | Medicinal (Cold and cough) | 0.10 | 0.30 | | 0.30 | | 0.0050 | | 0.33 | |  |
| 557 | *Cynodon dactylon* (L.) Pers. | Than grass | Upto 3000 | H | Wp |  | Medicinal (Nasal bleeding) | 0.10 | 0.60 | | 0.50 | | 0.0167 | | 0.58 | |  |
| 558 | *Dendrocalamus strictus* Nees |  | 700-1600 | T | Rt, Lf, Bk |  | Medicinal (Antifertility, cough, fever, tonic, veterinary); Edible; ; Religious | 0.80 | 0.30 | | 0.04 | | 0.0020 | | 0.50 | |  |
| 559 | *Desmostachya bipinnata* (L.)Stapf. | Kusha | 1200-1700 | H | WP |  | Medicinal (Tumours, kidney, bladder ailments) | 0.30 | 0.30 | | 0.20 | | 0.0033 | | 0.33 | |  |
| 560 | *Digitaria cruciata* (Nees ex Steud.) A.Camus |  | 1400-1800 | H | Lf |  | Fodder | 0.10 | 0.30 | | 0.30 | | 0.0050 | | 0.33 | |  |
| 561 | *Eleusine indica* Gaertn. |  | 800-1800 | H | WP |  | Medicinal (Fever) | 0.10 | 0.30 | | 0.30 | | 0.0050 | | 0.33 | |  |
| 562 | *Eragrotis unioloides* (Betz.) Nees. |  | 700-2000 | H | Wp |  | Fodder | 0.10 | 0.30 | | 0.30 | | 0.0050 | | 0.33 | |  |
| 563 | *Heteropogon contortus* L. | Kumbri | 800-1700 | H | WP |  | Medicinal (Cuts and bruises for quick healing) | 0.20 | 0.30 | | 0.04 | | 0.0013 | | 0.42 | |  |
| 564 | *Imperata cylindrica* (L.) Raeusch. |  | 800-1900 | H | AP, Rt |  | Medicinal (Antidote, fever, intestinal parasites, liver complaints, piles, tonic) | 0.50 | 0.30 | | 0.30 | | 0.0050 | | 0.33 | |  |
| 565 | *Oplismenus compositus* (L.) P.Beauv. |  | 700-2000 | H | Wp |  | Fodder | 0.10 | 0.30 | | 0.30 | | 0.0050 | | 0.33 | |  |
| 566 | *Oplismenus latifolius* Haenke ex Steud. |  | 1500-2000 | H | Wp |  | Fodder | 0.10 | 0.30 | | 0.30 | | 0.0050 | | 0.33 | |  |
| 567 | *Panicum psilopodum* Trin. |  | 700-1100 | H | Wp |  | Fodder | 0.10 | 0.30 | | 0.30 | | 0.0050 | | 0.33 | |  |
| 568 | *Saccharum spontaneum* L. | Surad | 800-2000 | H | Lf |  | Medicinal (Asthma, cholera, diarrhoea); Fodder | 0.40 | 0.60 | | 0.08 | | 0.0053 | | 0.67 | |  |
| 569 | *Setaria glauca* (L.) P.Beauv |  | 500-2000 | H | Sd |  | Edible | 0.10 | 0.30 | | 0.30 | | 0.0050 | | 0.33 | |  |
| 570 | *Themeda anathera* Hack. - GBIF |  | 700-2000 | H | Wp |  | Fodder | 0.10 | 0.30 | | 0.30 | | 0.0050 | | 0.33 | |  |
|  | **Podophyllaceae** |  |  |  |  |  |  |  |  | |  | |  | |  | |  |
| 571 | *Podophyllum hexandrum* Royle | Bankakri | 2800-4500 | H | Wp, Rf, Fr, Sd |  | Medicinal (Cough, purgative, chronic, constipation, tuberculosis, hepatic, stimulant, vermifuge, kill worms, gynecological disorder, skin disease, tumors, cancer, childbirth, cuts, wounds, diarrhea, gastric ulcer, hepatic disease, bloating in cattle, appetizer, appetizer) | 2.20 | 0.90 | | 0.80 | | 0.0400 | | 0.83 | |  |
|  | **Polygonaceae** |  |  |  |  |  |  |  |  | |  | |  | |  | |  |
| 572 | *Bistorta amplexicaulis* (D. Don) Greene | Mindle/Kutrya/Amli/Masloon | 2000-3500 | H | Wp |  | Medicinal (Wound in the eyes). | 0.10 | 0.30 | | 0.30 | | 0.0050 | | 0.33 | |  |
| 573 | *Fagopyrum dibotrys* (D. Don) Hara | Ban Paphra | 1300-2000 | H | Lf |  | Medicinal (Insect bite); Edible | 0.20 | 0.30 | | 0.30 | | 0.0050 | | 0.33 | |  |
| 574 | *Fagopyrum esculentum (L)* Moench | Helangala | 1200-4100 | H | Sd, Lf , Rt |  | Medicinal (Lung disorders, rheumatism, typhoid, urine complaints); Edible | 0.50 | 0.90 | | 0.20 | | 0.0200 | | 0.92 | |  |
| 575 | *Oxyria digyna* (L.) Hill | Chukru | 2400-5000 | H | Lf , Wp |  | Medicinal (Stomach disorders); Edible | 0.20 | 0.30 | | 0.02 | | 0.0007 | | 0.42 | |  |
| 576 | *Persicaria amplexicaulis* (D.Don) Ronse Decr |  | 1500-2600 |  | Lf, Rt |  | Medicinal (Insect sting) | 0.10 | 0.60 | | 0.50 | | 0.0167 | | 0.58 | |  |
| 577 | *Persicaria hydropiper* (L.) Delarbre | Ganeri | 900-2400 | H | Lf | Least Concern | Edible | 0.10 | 0.30 | | 0.40 | | 0.0067 | | 0.33 | |  |
| 578 | *Persicaria nepalensis* (Meisn.) Miyabe | Gosumbi/Trod | 2000-3500 | H | Lf , Fl |  | Medicinal (Piles, Swelling) | 0.20 | 0.60 | | 0.50 | | 0.0167 | | 0.58 | |  |
| 579 | *Persicaria plebium* R.Br. |  | 800-2200 | H | WP |  | Medicinal (Cough, gastric problems) | 0.20 | 0.30 | | 0.30 | | 0.0050 | | 0.33 | |  |
| 580 | *Persicaria vivipara* (L.) Ronse Decr. | Raktmundi | 3300-3500 | H | Lf |  | Medicinal (Constipation, cough) | 0.20 | 0.30 | | 0.30 | | 0.0050 | | 0.33 | |  |
| 581 | *Polygonum affine* Don |  | 1600–4800 | H | Rt |  |  | 0.00 | 0.30 | | 0.30 | | 0.0050 | | 0.33 | |  |
| 582 | *Polygonum amplexicaule* D. Don | Ban madua | 1500-3500 | H | Rt, Lf |  | Medicinal (Cough, dysentery, tonic, haemostasis,, excess dosecause abortion, wounds, heart buring sensation); Fodder | 0.80 | 0.30 | | 0.04 | | 0.0013 | | 0.42 | |  |
| 583 | *Polygonum aviculare* L. | Nadi | 2200-3800 | H | Areial part |  | Medicinal (Pneumonia); Edible | 0.20 | 0.30 | | 0.04 | | 0.0013 | | 0.42 | |  |
| 584 | *Polygonum capitatum* Buch.-Ham. ex D. Don |  | 700-2000 | H | Wp |  | Medicinal (Antidote in snake bite, boils, insectstings) | 0.30 | 0.30 | | 0.30 | | 0.0050 | | 0.33 | |  |
| 585 | *Polygonum hydropiper* L. |  | 1200-2200 | H | Lf |  | Medicinal (Tongue infection in cattle) | 0.10 | 0.30 | | 0.30 | | 0.0050 | | 0.33 | |  |
| 586 | *Polygonum nepalense* Meissn. | Trod | 1000-2000 | H | Lf |  | Medicinal (Swelling); Edible | 0.20 | 0.30 | | 0.04 | | 0.0013 | | 0.42 | |  |
| 587 | *Polygonum plebium* R.Br. |  | 1200-2600 | H | AP, Rt |  | Medicinal (Baldness, lung disease, diarrhoea, dysentery) | 0.40 | 0.30 | | 0.04 | | 0.0007 | | 0.33 | |  |
| 588 | *Polygonum polystachyum* Wall. ex Meissn. |  | 3000-3500 | H | AP |  | Medicinal (Rhamnacin, quercetin, isoquercitrin) | 0.30 | 0.30 | | 0.30 | | 0.0050 | | 0.33 | |  |
| 589 | *Polygonum recumbens* Royle ex Bab. |  | 1800–2700 | H | Wp |  | Medicinal (Abscess, boil, skin disease, blood purifier, carbuncle, healing) | 0.60 | 0.60 | | 0.50 | | 0.0167 | | 0.58 | |  |
| 590 | *Polygonum rumicifolium* Royle ex Bab. |  | 3200–4400 | S | Lf , Fl, Rt |  | Medicinal (Abscess, antidote to aconite poison, diarrhoea, giddiness, headache, thirst) | 0.60 | 0.30 | | 0.30 | | 0.0050 | | 0.33 | |  |
| 591 | *Polygonum stewartianum* Diels. |  | 3500–4300 | H | Wp |  | Medicinal (Tonic) | 0.10 | 0.30 | | 0.30 | | 0.0050 | | 0.33 | |  |
| 592 | *Polygonum viviparum* L. | Anjwar | 3000-5000 | H | Wp |  | Medicinal (Dysentery, blood pressure, internal injuries and wounds, pain) | 0.40 | 0.30 | | 0.30 | | 0.0050 | | 0.33 | |  |
| 593 | *Rheum australe* D. Don | Revandchini/Chukri, Dolu ,chuche | 2500-4200 | H | Rt |  | Medicinal (Swelling, fracture, tooth cleaning, abdominal pain, appetite, asthma, bronchitis, cuts, dysentery, laxative, eye disorder, sprain, swelling, ulcer, wounds) | 1.50 | 1.20 | | 0.80 | | 0.0533 | | 1.08 | |  |
| 594 | *Rheum emodi* Wall. ex Meissn. | Tukshu, Lichu, Artho, Chucha | 2000-4000 | H | Rt |  | Medicinal (Cancers, jaundice, headache, migraine, paralysis, sciatica, asthma, diarrhoea, liver disorders) | 0.90 | 0.30 | | 0.30 | | 0.0050 | | 0.33 | |  |
| 595 | *Rheum moocroftianum* Royle | Riyond chini | 3500–4800 | H | Rt |  | Medicinal (Cuts, wounds, appetite, Purgative) | 0.40 | 0.60 | | 0.40 | | 0.0133 | | 0.58 | |  |
| 596 | *Rheum spiciforme* Royle | Chuchi | 3000-4000 | H | Rt |  | Medicinal (Wounds, boils, cuts, abdominal diseases) | 0.40 | 0.30 | | 0.20 | | 0.0033 | | 0.33 | |  |
| 597 | *Rheum webbianum* Royle | Reward chini | 2400-4200 | H | Lf , Rt |  | Medicinal (Abdominal disease, appetite, boils, astringent, purgative, wounds, Abdominal disorder, boils, astringent, purgative, wounds) | 1.10 | 0.30 | | 0.20 | | 0.0033 | | 0.33 | |  |
| 598 | *Rumex acetosa* L. | Chukil | 3000-3600 | H | Lf, AP,Fr |  | Medicinal (Appetizer, laxative, stomach disease) | 0.30 | 0.30 | | 0.30 | | 0.0050 | | 0.33 | |  |
| 599 | *Rumex hastatus* D. Don | Khatti butti/Almoru | 700-2500 | H | Lf , Wp |  | Medicinal (Foot disease of the animal, nasal bleeding. Cuts, wounds, anti nettle sting); Edible | 0.50 | 0.60 | | 0.08 | | 0.0053 | | 0.67 | |  |
| 600 | *Rumex nepalensis* Spreng. | Albar, Malora | 800-4000 | H | Lf , Rt, Twig |  | Medicinal (Boils, colic, cooling, diuretic, purgative, scurvy, swelling of muscles, antiallergic, nepalensis, dymenorrhoea, swelling of muscle, stomachache); Fodder | 1.30 | 0.90 | | 0.30 | | 0.0300 | | 0.92 | |  |
|  | **Portulacaceae** |  |  |  |  |  |  |  |  | |  | |  | |  | |  |
| 601 | *Portulaca oleracea* L. | Dhupdu |  | H | Lf |  | Medicinal (skin rashes and cuts) | 0.10 | 0.30 | | 0.30 | | 0.0050 | | 0.33 | |  |
|  | **Primulaceae** |  |  |  |  |  |  |  |  | |  | |  | |  | |  |
| 602 | *Androsace rotundifolia* Hardw. | Zigsolo marpo | 2700-3400 | H | AP |  | Medicinal (Stomachache) | 0.10 | 0.30 | | 0.30 | | 0.0050 | | 0.33 | |  |
| 603 | *Embelia tsjeriam-*cottam (Roem. & Schult.) A. DC. | | Up to 1400 | S | Wp |  | Medicinal (Blood purification, bronchitis, cholera, fever, itch, pneumonia, pregnancy problems, sores, throat complaints, ulcers) | 1.00 | 0.30 | | 0.30 | | 0.0050 | | 0.33 | |  |
| 604 | *Primula denticulata Sm.* |  | 3000-3800 | H | AP, Fl, Rt |  | Medicinal (Diabetes, headache, appetizer, liver problem, giddiness, pulmonary disease, urinary ailments, kill lice) | 0.80 | 0.30 | | 0.30 | | 0.0050 | | 0.33 | |  |
| 605 | *Primula floribunda* Wall. | Phool | 1000-2000 | H | Rt, Lf |  | Washing | 0.10 | 0.30 | | 0.30 | | 0.0050 | | 0.33 | |  |
|  | **Pteridaceae** |  |  |  |  |  |  |  |  | |  | |  | |  | |  |
| 606 | *Adiantum capillus–*veneris L. | Parshosan |  | Fern |  | Least Concern | Medicinal (Antibacterial, antiimplantation, antihyperglycemic, hypoglycemic, antiyeast, antiviral activities) | 0.60 | 0.90 | | 0.80 | | 0.0400 | | 0.83 | |  |
| 607 | *Adiantum lunulatum* Burm. f. | Dungtuli/ Hansraj | 1000-3000 | Fern | Fronds (Lf ) |  | Medicinal (Fevers, elephantiasis) | 0.10 | 0.30 | | 0.30 | | 0.0050 | | 0.33 | |  |
| 608 | *Adiantum pedatum* L. |  |  | Fern |  |  | Medicinal (Fevers, elephantiasis) | 0.00 | 0.30 | | 0.30 | | 0.0050 | | 0.33 | |  |
| 609 | *Adiantum venustum* D. Don |  | 2000-2700 | Fern | Fronds (Lf ) |  | Medicinal (Inflammatory diseases of chest, opthalmia, biliousness and hydro- phobia, scorpion bites, expectorant) | 0.50 | 0.90 | | 0.70 | | 0.0350 | | 0.83 | |  |
| 610 | *Pteridium aquilinum* (L.) Kuhn | Nanoor | 1600-2500 |  | Ap | Least Concern | Roof thatching; Fodder | 0.20 | 0.60 | | 0.01 | | 0.0008 | | 0.75 | |  |
|  | **Punicaceae** |  |  |  |  |  |  |  |  | |  | |  | |  | |  |
| 611 | *Punica granatum* L. | Anar dana | upto 2000 | T | Lf , Fr | Least Concern | Medicinal (Stomach disorders, bleeding of child birth and miscarriage vomiting, pimples, antihelmentic, stomachache, eye complaints, diarrohea, dysentery, bronchitis, cholera, astringent); Edible | 1.20 | 1.20 | | 0.30 | | 0.0400 | | 1.17 | |  |
|  | **Ranunculaceae** |  |  |  |  |  |  |  |  | |  | |  | |  | |  |
| 612 | *Aconitum chasmanthum* Stapf. ex Holms. |  | 3000–4200 | H | Rt | Critically Endangered | Medicinal (Rheumatism) | 0.10 | 0.30 | | 0.30 | | 0.0050 | | 0.33 | |  |
| 613 | *Aconitum dienorrhizum* - Stapf. | Vatsnaba/ Mohra | 2500-3300 | H | Rt |  | Medicinal (Poison, sedative) | 0.20 | 0.30 | | 0.30 | | 0.0050 | | 0.33 | |  |
| 614 | *Aconitum falconeri* Stapf. var. latilobum |  | Above 3000 | H | Rt |  | Medicinal (Diarrhoea, fever, rheumatism) | 0.20 | 0.30 | | 0.30 | | 0.0050 | | 0.33 | |  |
| 615 | *Aconitum ferox* Wall. |  | 2100–3800 | H | Rt |  | Medicinal (Stimulant, cardiac tonic, febrifuge) | 0.30 | 0.30 | | 0.30 | | 0.0050 | | 0.33 | |  |
| 616 | *Aconitum heterophyllum* Wall.ex Royle | Atis/ Patis/ Karvi Patis | 3000-5000 | H | Rt | Endangered | Medicinal (Anthelmintic, cold, cough, diarrhoea, dyspepsia, gall bladder, abdominal pain, gastric, piles, vomits, worms, stomachache, dihydroatisine, hetidine, fever) | 1.50 | 1.20 | | 0.90 | | 0.0600 | | 1.08 | |  |
| 617 | *Aconitum laeve* Royle |  | 2000–3500 | H | Rt |  | Medicinal (Antihelminthic, cough, diarrhoea, digestive complaints, dysentery, fever, gastric, stomach ache, vomiting) | 0.90 | 0.30 | | 0.20 | | 0.0033 | | 0.33 | |  |
| 618 | *Aconitum spicatum* (Bruhl) Stapf. |  | 3400–4500 | H | Rt |  | Medicinal (Antipyretic, analgesic) | 0.20 | 0.30 | | 0.30 | | 0.0050 | | 0.33 | |  |
| 619 | *Aconitum violaceum* Jacquem exstapf. (H) | Mithi Patish, MithaTelia | 3500-4000 | H | Rt | Vulnerable | Medicinal (Gastrointestinal complaints, renal pain, rheumatism, stomach ache, cough) | 0.50 | 0.60 | | 0.50 | | 0.0167 | | 0.58 | |  |
| 620 | *Actaea spicata* Wall. ex Royle |  | 2200-3600 | H | Rt |  | Medicinal (Menustral flow) | 0.10 | 0.30 | | 0.30 | | 0.0050 | | 0.33 | |  |
| 621 | *Adonis chrysocyathus* Hk.f. & Th. |  | 2700–4600 | H | Wp |  | Poison | 0.10 | 0.30 | | 0.30 | | 0.0050 | | 0.33 | |  |
| 622 | *Anemone obtusiloba* D. Don |  | 2800-3800 | H | Rt, Sd |  | Medicinal (Menorrhoea, rheumatism, purgative, cooling) | 0.40 | 0.60 | | 0.50 | | 0.0167 | | 0.58 | |  |
| 623 | *Anemone polyanthes* D.Don |  | 1300-2000 | H | Rt | 7 | Medicinal (Purgative) | 0.10 | 0.30 | | 0.30 | | 0.0050 | | 0.33 | |  |
| 624 | *Anemone rivularis* Buch.-Ham. | Chutrak | 2600-3500 | H | Wp |  | Medicinal (Ear complaints, appetizer, gastric, headache, wounds, sores, vetinary medicine) | 0.70 | 0.60 | | 0.50 | | 0.0167 | | 0.58 | |  |
| 625 | *Anemone rupicola* Cambess | Kakrya |  | H | Lf |  | Medicinal (Ears with pus) | 0.10 | 0.30 | | 0.30 | | 0.0050 | | 0.33 | |  |
| 626 | *Anemone vitifolia* Buch.-Ham. ex DC. |  | 1800-3200 | H | Rt, Lf |  | Medicinal (Scalpto treat lice) | 0.10 | 0.30 | | 0.30 | | 0.0050 | | 0.33 | |  |
| 627 | *Aquilegia fragrans* Benth. | Lande, kumuk | 2700-4000 | H | Fl |  | Medicinal (Headache, boils, snake bite, body pains) | 0.40 | 0.30 | | 0.30 | | 0.0050 | | 0.33 | |  |
| 628 | *Aquilegia pubiflora* Wall. ex Royle |  | 2500-3400 | H | AP |  | Medicinal (Ring worm, eczema) | 0.00 | 0.30 | | 0.30 | | 0.0050 | | 0.33 | |  |
| 629 | *Caltha palustris* L. | Butti | 2400-4200 | H | Lf , Wp | Least Concern | Medicinal (Worm infected, sores, wound, gonorrhea, cattle wound, hand clean) | 0.60 | 0.60 | | 0.50 | | 0.0167 | | 0.58 | |  |
| 630 | *Clematis barbellata* Edgew. | Baldkuja | 2800-3500 | S | Rt, Lf |  | Medicinal (Skin diseases, sores, tumors, Itching, skin disorder, stomache) | 0.60 | 0.90 | | 0.70 | | 0.0350 | | 0.83 | |  |
| 631 | *Clematis buchananiana* DC. | Veraphul | 1200-1800 | Climber | Rt, Lf |  | Medicinal (Skin diseases, migraine, sores, tumors) | 0.40 | 0.60 | | 0.50 | | 0.0167 | | 0.58 | |  |
| 632 | *Clematis dubia* (Endl.) P.S.Green |  | 1400-2000 | S | Lf, Rt |  | Medicinal (Toothache) | 0.10 | 0.30 | | 0.20 | | 0.0033 | | 0.33 | |  |
| 633 | *Clematis graveolens* Lindl. |  | 900–3000 | S | Wp |  | Medicinal (Skin aliments, antiseptic) | 0.20 | 0.30 | | 0.30 | | 0.0050 | | 0.33 | |  |
| 634 | *Clematis montana* Buch.-Ham. ex DC. |  | 1800-2600 | S | Rt |  | Medicinal (Cuts and wounds) | 0.10 | 0.30 | | 0.30 | | 0.0050 | | 0.33 | |  |
| 635 | *Delphinium cashmerianum* Royle |  | 2700–4800 | H | Wp |  | Medicinal (Abdominal pain, dropsy, dyspepsia, headache, renal pain, stomach ache, swelling, ascites, cough, cold, swell, cuts, wounds) | 1.30 | 0.60 | | 0.50 | | 0.0167 | | 0.58 | |  |
| 636 | *Delphinium denudatum* Wall. | Laskar, Nirbishi | 1500-2800 | H | Rt |  | Medicinal (Abdominal pain, anthelmintic, respiratory complaint, toothache, ulcer, vetlice, ticks) | 0.70 | 0.90 | | 0.80 | | 0.0400 | | 0.83 | |  |
| 637 | *Delphinium vestitum* Wall. ex Royle |  | 2700–4700 | H | WP, Fl, Lf |  | Medicinal (Ascites, abdominal pain, cough, cold, dropsy, dyspepsia, headache, renal pain, swell, stomachic, cuts, wounds, snake bite) | 1.30 | 0.90 | | 0.80 | | 0.0400 | | 0.83 | |  |
| 638 | *Delphinium viscosum* Hk. & Th. |  | 3000–5200 | H | Rt, Wp |  | Medicinal (Oedema, rheumatism, antidote to snake bite, cuts, wounds, diarrhea) | 0.60 | 0.60 | | 0.50 | | 0.0167 | | 0.58 | |  |
| 639 | *Ranunculus abortivus* L. |  | 1600-2800 | H | WP |  | Medicinal (Urinary tract infection) | 0.10 | 0.30 | | 0.30 | | 0.0050 | | 0.33 | |  |
| 640 | *Ranunculus diffusus* DC. |  | 1500-2000 | H | Wp |  | Medicinal (Boils) | 0.00 | 0.60 | | 0.50 | | 0.0167 | | 0.58 | |  |
| 641 | *Ranunculus hirtellus* Royle | Goodi | 2800-5500 | H | Rt, Ap |  | Medicinal (Swelling, counter irritant, anthelmintic, vermicidal, cooling, emoll, wounds) | 0.70 | 0.60 | | 0.50 | | 0.0167 | | 0.58 | |  |
| 642 | *Ranunculus laetus*Wall. ex Royle | Phool | 1200-2700 | H | Ap , Rts |  | Medicinal (Skin infections, wounds, antimicrobial activities); Fodder | 0.40 | 0.60 | | 0.08 | | 0.0053 | | 0.67 | |  |
| 643 | *Thalictrum cultratum* Wall. | Mamiri | 1800-2000 | H | Ap |  | Fodder | 0.10 | 0.30 | | 0.30 | | 0.0050 | | 0.33 | |  |
| 644 | *Thalictrum foliolosum* DC. | Barmot | 1300-3400 | H | Rts |  | Medicinal (Abdominal pain, blood purifier, eye diseases, leucoderma, rheumatism, stomach pain, gastric trouble, boils, earache, eczema, piles, rheumatism, gout, tonic, toothache); Fodder | 1.60 | 0.90 | | 0.20 | | 0.0200 | | 0.92 | |  |
| 645 | *Thalictrum minus* L. var. majus (Jacq.) Hk. f. et Th. | | 2000–4000 | H | Wp |  | Medicinal (Eye disorders, fever) | 0.10 | 0.30 | | 0.20 | | 0.0033 | | 0.33 | |  |
| 646 | *Thalictrum pauciflorum* Royle | Gadbini | 3000-3800 | H | Lf , Sd, Rt |  | Medicinal (Digestive disorders of cattle) | 0.10 | 0.30 | | 0.20 | | 0.0033 | | 0.33 | |  |
| 647 | *Thalictrum reniforme* Wall |  | 2800-3400 | H | Rt |  | Medicinal (Cataract) | 0.10 | 0.30 | | 0.30 | | 0.0050 | | 0.33 | |  |
| 648 | *Thalictrum secundum Edgew.* | Katmeena | 2600-3050 |  | Rt |  | Medicinal (Swelling) | 0.10 | 0.30 | | 0.30 | | 0.0050 | | 0.33 | |  |
|  | **Rhamnaceae** |  |  |  |  |  |  |  |  | |  | |  | |  | |  |
| 649 | *Rhamnus purpureus* Edgew. | Chaunsha | 1500–3000 | S | St, Fr, Lf, Wd |  | Medicinal (Purgative, digestive disorders); Agricultural tools; Fodder | 0.40 | 1.20 | | 0.05 | | 0.0100 | | 1.25 | |  |
| 650 | *Rhamnus triqueter* (Wall.) Brandis | Gonta | 1500–2100 | T | Fr, Bk |  | Medicinal (Dysentery, diarrohea) Blood purifier, boils, scabies, skin disease, veterinary tonic | 0.60 | 1.20 | | 0.70 | | 0.0467 | | 1.08 | |  |
| 651 | *Rhamnus virgatus* Roxb. |  | 1200-2600 | H | St,Fr,Br |  | Medicinal (Emetic, purgative, eczema, ringworm, affection of spleen) | 0.50 | 0.60 | | 0.50 | | 0.0167 | | 0.58 | |  |
| 652 | *Zizyphus mauritiana* Lam. | Ber | 1100-1400 | T | St, Br, Fr, Lf |  | Medicinal (Antiemetic, astringent, blood purifier, digestive problem, hair fall, oedema, ulcer, whooping cough, stomachache, purgative, sedative, cholera, dysentery, diarrohea); Edible; Fodder | 1.60 | 0.60 | | 0.05 | | 0.0050 | | 0.75 | |  |
| 653 | *Zizyphus oxyphylla* Edgrew. | Baer | 700-1100 | Sh |  |  | Medicinal (Cough) | 0.10 | 0.30 | | 0.30 | | 0.0050 | | 0.33 | |  |
| 654 | *Zizyphus rugosa* Lamk. | Baer | 700-1000 | Sh | Bk, Fl, St, Fr |  | Medicinal (Astringent, diarrohea, hypotensive) | 0.30 | 0.30 | | 0.30 | | 0.0050 | | 0.33 | |  |
|  | **Rosaceae** |  |  |  |  |  |  |  |  | |  | |  | |  | |  |
| 655 | *Agrimonia pilosa* Ledlb. | Kuri | 1500-2800 | H | Ap, Rt |  | Medicinal (Cough, cold, urinary problems) | 0.30 | 0.60 | | 0.50 | | 0.0167 | | 0.58 | |  |
| 656 | *Cerasus cerasoides* (Buch.-Ham. ex D.Don) S.Y.Sokolov | Pajja/ Padam/ Padmakasht | 1200-2400 | T | Fr, Sd |  | Medicinal (antioxidant, antimicrobial, anthelmintic, antiepileptic, antitumor and antimutagenic, neuropharmacoligical and radioprotective) | 0.70 | 0.30 | | 0.30 | | 0.0050 | | 0.33 | |  |
| 657 | *Cotoneaster bacillaris* Wall. ex Lindl. | Rhiunsh | 1700–3200 | S | Lf |  | Medicinal (Scabies, rheumatism, arthritis, cuts, wounds) | 0.50 | 0.60 | | 0.50 | | 0.0167 | | 0.58 | |  |
| 658 | *Cotoneaster marginatus* Sch. |  | 2000–2500 | S | Above part |  | Medicinal (Cuts, wounds) | 0.20 | 0.30 | | 0.30 | | 0.0050 | | 0.33 | |  |
| 659 | *Cotoneaster microphyllus* Wall. ex Lindl. | Chinchri | 2000-3500 |  | Rt, Lf |  | Medicinal (Ulcers) | 0.10 | 0.30 | | 0.20 | | 0.0033 | | 0.33 | |  |
| 660 | *Cotoneaster nummularia* Fisch. & Meyer |  | 600–3600 | S | Fr, Bk, Lf |  | Medicinal (Cuts, wounds) | 0.20 | 0.30 | | 0.20 | | 0.0033 | | 0.33 | |  |
| 661 | *Cotoneaster rotundifolius* Wall. ex Lindl. | Leo/Loon |  | S | Lf |  | Edible; Walking sticks and agriculture tools; Fodder | 0.40 | 0.30 | | 0.06 | | 0.0020 | | 0.42 | |  |
| 662 | *Duchesnea indica* Focke |  | 1600-2500 | H | Lf,Fl, Fr, Rt |  | Medicinal (Fever) | 0.10 | 0.30 | | 0.30 | | 0.0050 | | 0.33 | |  |
| 663 | *Fragaria nubicola* Lindley ex Lacaita | Kida-bhumla/Mewa/Buti | 2000-4000 | H | Ap , Fr |  | Medicinal (Burns, cuts, rheumatism, wounds, fever, earache); Edible | 0.80 | 1.20 | | 0.70 | | 0.0467 | | 1.08 | |  |
| 664 | *Fragaria vesca L.* |  | 700-2000 | H | Fr |  | Edible | 0.10 | 0.60 | | 0.40 | | 0.0133 | | 0.58 | |  |
| 665 | *Geum elatum* Wall. ex G. Don |  | 3400–4200 | H | Wp |  | Medicinal (Astringent, dysentery, diarrhoea) | 0.30 | 0.90 | | 0.80 | | 0.0400 | | 0.83 | |  |
| 666 | *Malus baccata* (L) Borkh | Barol | 1500-3600 | T | Fr | Least Concern | Edible | 0.10 | 0.30 | | 0.30 | | 0.0050 | | 0.33 | |  |
| 667 | *Potentilla argyrophylla* Wall .ex Lehm. | Ratanjot | 2400-4200 | H | Lf |  | Medicinal (Diarrhea, arthritis, kidney stones, angina pectoris, toothache, gingivitis, wound as analgesic) | 0.70 | 0.30 | | 0.50 | | 0.0083 | | 0.33 | |  |
| 668 | *Potentilla atrosaguinea* Lodd. |  | 2800-3600 | H | Lf |  | Medicinal (Wounds as analgesic) | 0.10 | 0.60 | | 0.50 | | 0.0167 | | 0.58 | |  |
| 669 | *Potentilla fruticosa Lim.* |  | 3300-4000 | S | AP |  | Medicinal (Astringent) | 0.10 | 0.30 | | 0.30 | | 0.0050 | | 0.33 | |  |
| 670 | *Potentilla fulgens* Wall. ex Hk.f. |  | 1600–4800 | H | Rt |  | Medicinal (Gum, tooth complaints, tonic) | 0.30 | 0.60 | | 0.50 | | 0.0167 | | 0.58 | |  |
| 671 | *Potentilla indica* (Andrews) Th. Wolf | Bada Mewa | 700-2800 | H | Fr |  | Edible | 0.10 | 0.30 | | 0.30 | | 0.0050 | | 0.33 | |  |
| 672 | *Potentilla nepalensis* Hook. | Dori Ghas | 2000-2700 | H | Rt |  | Medicinal (Cuts, burns, wounds) | 0.30 | 0.30 | | 0.30 | | 0.0050 | | 0.33 | |  |
| 673 | *Prinsepia utilis* Royle | Bakhel | 1100-3000 | S | Rt, Sd |  | Medicinal (Neutralize the effect of poison intake, wounds. Burns, cuts, rheumatic) | 0.40 | 1.20 | | 0.80 | | 0.0533 | | 1.08 | |  |
| 674 | *Prunus armeniaca* Linn | Wild apricot/Khumani | 1600-3500 | T | Fr, Sd | Endangered | Medicinal (Constipationin cattles, appetizer, body massage, hair tonic, rheumatism) Edible; Local wine | 0.10 | 0.90 | | 0.14 | | 0.0210 | | 1.00 | |  |
| 675 | *Prunus cerasoides* D.Don | Pajja | 1200-2500 | T | St Bk | Least Concern | Medicinal (Joint pains) Edible; Fodder; Dye | 0.30 | 0.30 | | 0.01 | | 0.0003 | | 0.50 | |  |
| 676 | *Prunus cornuta* (Wall. ex Royle) Steud. | Jamu | 1800-3600 | T | Fr, Sd |  | Medicinal (Cure diabetes); Edible | 0.20 | 0.30 | | 0.04 | | 0.0013 | | 0.42 | |  |
| 677 | *Prunus mira* Koehne | Behmi |  | T | Fr, Sd | Endangered | Edible; Oil used for body massaging | 0.20 | 0.30 | | 0.04 | | 0.0013 | | 0.42 | |  |
| 678 | *Prunus persica* L. | Wild peech/Aaru | 1000-3000 | T | Sd, Lf | Least Concern | Medicinal (Antiemetic, diuretic, mouth ulcers, headache, toothbrush, stomachache, eczema, antiscorbutic, purgative, scabies, whooping cough); Edible; Fuel; Fodder | 1.40 | 0.60 | | 0.01 | | 0.0012 | | 0.75 | |  |
| 679 | *Pyracantha crenulata (*D.Don) M. Roemer | Choota Seb |  |  | Fr, St |  | Edible; Walking sticks | 0.20 | 0.30 | | 0.01 | | 0.0003 | | 0.50 | |  |
| 680 | *Pyrus pashia* Buch.-Ham. ex D. Don | Kainth/ Shegal | 700-2600 | T | Lf | Least Concern | Medicinal (Eye disorder, digestive disorders); Edible; Fodder, Fuel; Religious and cultural significance | 0.60 | 0.30 | | 0.01 | | 0.0004 | | 0.58 | |  |
| 681 | *Rosa brunonii* Lindl. | Kunja | 700-2000 | Sh | Rt |  | Medicinal (Pain, eye complaints); Fuel; Fodder | 0.40 | 0.30 | | 0.30 | | 0.0050 | | 0.33 | |  |
| 682 | *Rosa canina* L. | Kuin |  | S | Fl, Fr, Lf |  | Edible: Fodder, Fencing, Incense | 0.30 | 0.30 | | 0.01 | | 0.0008 | | 0.58 | |  |
| 683 | *Rosa macrophylla* Lindl. | Jungli gulab | 2100-3800 | S | Fl |  | Medicinal (Stomachache) | 0.10 | 0.60 | | 0.60 | | 0.0200 | | 0.58 | |  |
| 684 | *Rosa moschata* J. Herrmann | Kunja | 1000-2800 | S | Lf , Fl |  | Medicinal (Wounds, opthalmia, diarrhea) | 0.30 | 0.60 | | 0.50 | | 0.0167 | | 0.58 | |  |
| 685 | *Rosa sericea* Lindl. |  | 2000-3400 | S | Rt, Fr, Fl |  | Medicinal (Bleeding, stomach worms) | 0.20 | 0.30 | | 0.30 | | 0.0050 | | 0.33 | |  |
| 686 | *Rosa webbiana* Wall. ex Royle | Chawag | 2300–3800 | S | Lf |  | Medicinal (Hepatitis, jaundice, stomach ache) | 0.30 | 0.60 | | 0.50 | | 0.0167 | | 0.58 | |  |
| 687 | *Rubus biflorus* Buch.-Ham. ex Sm. | Akhaey/ Heer | 1500-2500 | S | Fr, Rt |  | Medicinal (Diarrohea); Edible stomachic | 0.20 | 0.60 | | 0.40 | | 0.0133 | | 0.58 | |  |
| 688 | *Rubus ellipticus* Sm. | Aakhe/Karer | 1100-2400 | S | Fr, Rt, Shoot |  | Medicinal (Dysentery, malaria, stomachache, worms); Edible; Fodder Fencing | 0.60 | 1.50 | | 0.03 | | 0.0107 | | 1.58 | |  |
| 689 | *Rubus foliolosus* D. Don |  | 1500-2000 | Sh | Fr, Rt |  | Medicinal (Dysentery); Edible | 0.20 | 0.30 | | 0.02 | | 0.0007 | | 0.42 | |  |
| 690 | *Rubus niveus* Thunb. | Khiradi/Aakhe/Karer | 2000-3000 | S | Rt, Fr |  | Medicinal (excessive bleeding during menstrual cycle, stomachache); Edible; Fodder; Fencing | 0.50 | 0.90 | | 0.01 | | 0.0014 | | 1.17 | |  |
| 691 | *Rubus paniculatus* Sm. | Kalanche, Kala akha | 1500-2600 | S | Lf, Fr |  | Medicinal (Diarrohea, stomach disorder); Edible | 0.30 | 0.60 | | 0.10 | | 0.0067 | | 0.67 | |  |
| 692 | *Sibbaldia bella* Sims. |  | 2700-3050 | S |  |  | Medicinal (Gastric trouble) | 0.10 | 0.30 | | 0.20 | | 0.0033 | | 0.33 | |  |
| 693 | *Sibbaldia cuneata* Hor. ex Kuntz |  | 2800-3050 | H | Fr, Rt |  | Medicinal (Bodyache) | 0.10 | 0.30 | | 0.30 | | 0.0050 | | 0.33 | |  |
| 694 | *Sorbaria tomentosa* (Lindl.) Rehder | Paddad |  | T | Lf , Wd |  | Medicinal (vermicide in animals); Fuel | 0.20 | 0.30 | | 0.04 | | 0.0013 | | 0.42 | |  |
| 695 | *Sorbus aucuparia* L. |  | 3000-3500 | S | Fr |  | Medicinal (Cough, cold) | 0.20 | 0.30 | | 0.30 | | 0.0050 | | 0.33 | |  |
| 696 | *Sorbus lanata* (D.Don) S. Schaur | Bhomphal | 2800-3800 | T | Fr, Lf |  | Fruits edible; Fodder | 0.20 | 0.30 | | 0.30 | | 0.0050 | | 0.33 | |  |
| 697 | *Sorbus ursina* (Wenzing.) Decne |  | 3200-3600 | S | Branches |  | Medicinal (Wound healing) | 0.10 | 0.30 | | 0.30 | | 0.0050 | | 0.33 | |  |
| 698 | *Spiraea canescens* D.Don. | Preud/Kati ,Sagal | 1800-2800 | S | St, Bk |  | Medicinal (Sores, wounds); Brooms and baskets (kirra) | 0.30 | 0.30 | | 0.04 | | 0.0013 | | 0.42 | |  |
|  | **Rubiaceae** |  |  |  |  |  |  |  |  | |  | |  | |  | |  |
| 699 | *Catunaregam spinosa* (Thunb.) Tirveng. | Madanphal | 900-1300 | H | Fl & Bk |  | Medicinal (Fever) | 0.10 | 0.30 | | 0.30 | | 0.0050 | | 0.33 | |  |
| 700 | *Galium aparine* L | Zangchi | 2000-3500 | H | Ap , Lf , Wp |  | Medicinal (Astringent, skin disease) | 0.20 | 0.60 | | 0.08 | | 0.0027 | | 0.58 | |  |
| 701 | *Galium asperifolium* Wall. |  | 2500-3700 | H | Wp |  | Medicinal (Bronchitis, tonsil, Skin disease, diuretic, urinary problem); Fodder | 0.60 | 0.30 | | 0.04 | | 0.0013 | | 0.42 | |  |
| 702 | *Galium rotundifolium* L. |  | 2700-3500 | H | Wp |  | Medicinal (Colic, dyspepsia, jaundice) | 0.30 | 0.90 | | 0.70 | | 0.0350 | | 0.83 | |  |
| 703 | *Hymenodictyon excelsum* (Roxb.) Wall. |  | 700-1100 | T | Rt, Bk, Lf |  | Medicinal (Abortificant, cholera, fever, gout, lactation, malaria); Edible | 0.40 | 0.30 | | 0.06 | | 0.0020 | | 0.42 | |  |
| 704 | *Leptodermis lanceolata* Wall. | Bilan | 800-3050 | S | Bk, Lf |  | Medicinal (Boils, Blisters in mouth, diarrhoea); Fodder | 0.40 | 0.60 | | 0.08 | | 0.0053 | | 0.67 | |  |
| 705 | *Oldenlandia corymbosa* L. |  | 700-1000 | H | Wp |  | Medicinal (Anthelmintic, diuretic, expectorant, liver tonic, jaundice, heat eruptions, constipation, flatulence, colic, cough, skin diseases, dyspepsia, leprosy, cough, bronchitis) | 1.50 | 0.30 | | 0.30 | | 0.0050 | | 0.33 | |  |
| 706 | *Pavetta indica* L. | Papri | 1000-1400 | S | Bk, Lf |  | Medicinal (Diarrhea and haemorrhages) | 0.20 | 0.30 | | 0.30 | | 0.0050 | | 0.33 | |  |
| 707 | *Randia tetrasperma* Lamk. | Kharnadu | 1000–2000 | S | Fr, Bk, Rt |  | Wash hair and clothes | 0.20 | 0.60 | | 0.50 | | 0.0167 | | 0.58 | |  |
| 708 | *Rubia cordifolia* L. | Jamithi | 1200-2600 | H | St, Rt, Lf |  | Medicinal (Antidote to scorpion bite, snake bite, astringent, chest complaints, leucoderma, inflammation, jaundice, liver complaints, paralysis, menorrhoea, urine complaints, ulcer, stomachache); Edible; Fodder, Misc. (branches used for making walking sticks) | 1.60 | 1.20 | | 0.02 | | 0.0065 | | 1.42 | |  |
|  | **Rutaceae** |  |  |  |  |  |  |  |  | |  | |  | |  | |  |
| 709 | *Aegle marmelos* (L.) Correa | Bilgiri/Bilpatri | 600-1100 | T | Lf , Fr, Bk |  | Medicinal (Digestive disorder, chronic dysentery, diarrhoea.); Religious | 0.40 | 0.60 | | 0.50 | | 0.0167 | | 0.58 | |  |
| 710 | *Boenninghausenia albiflora* (Hook.) Rchb. ex Meisn | Pisu mar butti | 600-3300 | H | Lf |  | Medicinal (Antiseptic, cuts, wounds, vomiting, dysentery, bed bug, antiseptic, cuts, wounds, vomiting, dysentery) | 1.10 | 0.90 | | 0.70 | | 0.0350 | | 0.83 | |  |
| 711 | *Dictamnus albus* L. |  | 1800–3300 | H | Rt |  | Medicinal (Skin diseases, arthritis, jaundice, scorbutic, scabies, eczema) | 0.60 | 0.30 | | 0.30 | | 0.0050 | | 0.33 | |  |
| 712 | *Glycosmis pentaphylla* (Retz.) DC | Gingging | 1100-1500 | S | Lf, Fr |  | Medicinal (Fevers, liver complaints and intestinal worms, ulcer) | 0.30 | 0.30 | | 0.30 | | 0.0050 | | 0.33 | |  |
| 713 | *Limonia acidissima L.* |  | 700-1000 | T | Rt, Bk, Lf , Rr |  | Medicinal (Diarrohea, cough, bronchitis, hiccough) | 0.40 | 0.30 | | 0.30 | | 0.0050 | | 0.33 | |  |
| 714 | *Murraya koenigii* (L.) Spreng. | Mitthi Nim | 150-1500 | S | Lf |  | Medicinal (Anthelmentic, diarrohea, dysentery, tonic, joint pain, swelling of foot and legs lactation, malaria fever); Edible | 0.80 | 0.60 | | 0.08 | | 0.0027 | | 0.58 | |  |
| 715 | *Murraya paniculata* (L.) Jack | Bajr-danti | 150-1500 | S | Lf , St |  | Medicinal (clean teeth); Fodder, | 0.20 | 0.60 | | 0.09 | | 0.0060 | | 0.67 | |  |
| 716 | *Skimmia anquetilia* Tayl. and Airy Shaw |  | 2400-3200 | S | Lf |  | Medicinal (Headache and freshness) | 0.10 | 0.30 | | 0.06 | | 0.0020 | | 0.42 | |  |
| 717 | *Skimmia laureola* (DC.) Siebold & Zucc. ex Walp. | Neir | 2400–3200 | S | Lf |  | Medicinal (Antiseptic, boils, gastric pains, rheumatism, scabies, smallpox, toothache) | 0.70 | 0.90 | | 0.70 | | 0.0350 | | 0.83 | |  |
| 718 | *Zanthoxylum armatum* DC. | Tirmir | 1100-1600 | S | Sd, Fr, Rt, Bk, St |  | Medicinal (Cough, cholera, fever, eczema, itching, leucodenna, clean teeth, toothache, mouth ulcers, antihelminthic, carminative,piles, rheumatism, smallpox, snakebite, stomach disorder,tonic, tooth complaints); Edible; Fuel; Walking sticks,; Condiments | 2.20 | 1.50 | | 0.04 | | 0.0196 | | 1.75 | |  |
|  | **Salicaceae** |  |  |  |  |  |  |  |  | |  | |  | |  | |  |
| 719 | *Populus ciliata* Wall. ex Royle | Pak butra | 2000-3500 | T | St, Br, Inf,Lf, Wd |  | Medicinal (Bone fracture, tonic, stimulant, blood purifier); Fuel; Fodder | 0.60 | 0.60 | | 0.01 | | 0.0012 | | 0.75 | |  |
|  | **Santalaceae** |  |  |  |  |  |  |  |  | |  | |  | |  | |  |
| 720 | *Osyris quadripartita* Salz. ex Decne. |  | 1500-2000 | Sh | Wp |  | Fodder | 0.10 | 0.30 | | 0.20 | | 0.0033 | | 0.33 | |  |
|  | **Sapindaceae** |  |  |  |  |  |  |  |  | |  | |  | |  | |  |
| 721 | *Acer caesium*Wall. ex Brandis | Acer/Kajlu/ Jawandali | 2100-3300 | T | Wd, Bk, Lf | Least Concern | Fodder | 0.10 | 0.30 | | 0.04 | | 0.0013 | | 0.42 | |  |
| 722 | *Aesculus indica* (Wall. ex Camb.) | Khanor/ Horsechestnut | 1600-2800 | T | Bk, Fr, Rt, Sd |  | Medicinal (Pregnancy food, excessive bleeding and pain during menses, anthelmintic, dislocated joints, diuretic, leucorrhoea, skin fissures, cracks, veterinary medicine, wounds); Fodder | 1.10 | 0.90 | | 0.30 | | 0.0300 | | 0.92 | |  |
| 723 | *Dodonaea viscosa* (L.) Jacq. | Mehndru | 1000-1800 | S | Lf , Sd |  | Medicinal (Cold, cuts, wounds, insect problem, menstrual flow, burns) | 0.60 | 0.60 | | 0.40 | | 0.0133 | | 0.58 | |  |
| 724 | *Sapindus mukorossi* Gaertn. | Ritha/Dodde | 1100-2500 | T | Fr, Sd |  | Medicinal (Expectorant, febrifuge, epilepsy, tonsilitis, antidandruff , snakebite, freckles, eczema); Fodder, Fuel; Misc. (Detergent) | 1.10 | 0.90 | | 0.01 | | 0.0022 | | 1.08 | |  |
|  | **Saurauiaceae** |  |  |  |  |  |  |  |  | |  | |  | |  | |  |
| 725 | *Bergenia ligulata* Engl. |  | 1600–3200 | H | Rt |  | Medicinal (Asthma, boils, cuts, wounds, burns, fever, liver complaints, ophthalmia, piles, thirst, kidney stones, urine complaints; diarrhoea of cattle) | 1.30 | 0.30 | | 0.30 | | 0.0050 | | 0.33 | |  |
| 726 | *Houttuynia cordata* Thunb. |  | 1500-2000 | H | Lf, Rt |  | Medicinal (Treatment of worms) | 0.10 | 0.30 | | 0.30 | | 0.0050 | | 0.33 | |  |
| 727 | *Saurauia napaulensis* DC. |  | 600–2000 | T | Bk | Least Concern | Poultice to extract splinters | 0.10 | 0.30 | | 0.30 | | 0.0050 | | 0.33 | |  |
|  | **Saxifragaceae** |  |  |  |  |  |  |  |  | |  | |  | |  | |  |
| 728 | *Astilbe rivularis* Buch.-Ham. ex Don |  | 1800-2800 | H | Lf |  | Medicinal (Blood purifier, toothache) | 0.20 | 0.30 | | 0.30 | | 0.0050 | | 0.33 | |  |
| 729 | *Bergenia ciliata* (Haw.) Sternb. | Pasahnbhed/ Patharchat | 2800-4000 | H | Rf, Rt |  | Medicinal (Kidney stones, menstrual pain ,fever, diarrhea, pulmonary infections, bruises and boils ) | 0.70 | 0.90 | | 0.70 | | 0.0350 | | 0.83 | |  |
| 730 | *Bergenia stracheyi* (Hook. fil. & Thomson) Engl. | Kapdolu | 2800-4001 | H | Rf, Rt |  | Medicinal (kidney stone, Antiascorbic, astringent, diuretic, fever, ophthalmia, tonic, cuts, wounds, piles) | 1.00 | 1.20 | | 0.80 | | 0.0533 | | 1.08 | |  |
| 731 | *Saxifraga parnassifolia* D. Don | Phah | 2800-3050 | H | Lf |  | Medicinal (Toothache and bloodpurifier) | 0.20 | 0.30 | | 0.30 | | 0.0050 | | 0.33 | |  |
|  | **Scrophulariaceae** |  |  |  |  |  |  |  |  | |  | |  | |  | |  |
| 732 | *Euphrasia himalayica* Wettst. |  | 3200-3800 | H | Lf |  | Medicinal (Eye disorder) | 0.10 | 0.30 | | 0.02 | | 0.0003 | | 0.33 | |  |
| 733 | *Lagotis cashmiriana* (Royle) Rupr. |  | 3300–4500 | H | Wp |  | Medicinal (Adulterant, fever, dyspepsia) | 0.30 | 0.30 | | 0.04 | | 0.0007 | | 0.33 | |  |
| 734 | *Lindenbergia indica* Vatke. |  | 800-1800 | H | Lf |  | Medicinal (Worms) | 0.10 | 0.90 | | 0.70 | | 0.0350 | | 0.83 | |  |
| 735 | *Pedicularis pectinata* Wall. ex Benth |  | 3200–3800 | H | Wp |  | Medicinal (Body ache, sedative) | 0.20 | 0.60 | | 0.60 | | 0.0200 | | 0.58 | |  |
| 736 | *Picrorhiza kurroa* Royle ex Benth. | Karu/Kutki | 3300-4800 | H | Rt, Rh, Lf, St |  | Medicinal (Fever, abdominal pain, anaemia, asthma, cholera, cold, diarrhoea, dysentery, fever, jaundice, stomach disorder, cardiotonic, cold, dyspepsia, diarrhoea, influenz, diuretic, hepato protective, indigenous system of medicine, jaundice, liver trouble, stomach, laxative, pure circulation blood, joint pains) | 2.50 | 1.20 | | 1.20 | | 0.0800 | | 1.08 | |  |
| 737 | *Scoparia dulcis* L. |  | 1500-1800 |  | WP |  | Medicinal (Cuts and wounds) | 0.10 | 0.30 | | 0.30 | | 0.0050 | | 0.33 | |  |
| 738 | *Scrophularia calycina* Benth. & Scroph. |  | 2600-3200 | H | AP,Lf |  | Medicinal (Stimulate appetite) | 0.10 | 0.30 | | 0.30 | | 0.0050 | | 0.33 | |  |
| 739 | *Scrophularia dentata* Royle ex Benth. |  | 3300-3700 | H | Lf |  | Medicinal (Appetizer) | 0.10 | 0.30 | | 0.30 | | 0.0050 | | 0.33 | |  |
| 740 | *Scrophularia himalensis* Royle ex Benth. |  | 3400-3800 | H | Lf |  | Insecticide | 0.10 | 0.60 | | 0.50 | | 0.0167 | | 0.58 | |  |
| 741 | *Scrophularia koelzii* Penn. | Pahari neem | 1500-3400 | H | Lf , Sd |  | Medicinal (Wounds, anantidote to snake venom) | 0.20 | 0.30 | | 0.30 | | 0.0050 | | 0.33 | |  |
| 742 | *Verbascum thapsus* L. | Kolomasta,Jangli tamaku | 1200-4000 | H | Lf, Ap, Fl |  | Medicinal (Asthma, cough, inflammation, leucoderrna, veterinary diseases, diarrhoea, dyesentry of sheep, poultice, skin disease, narcotic, fish poisoning, ear infection, Indigestion in cattle) | 1.30 | 1.50 | | 1.40 | | 0.1167 | | 1.33 | |  |
|  | **Simaroubaceae** |  |  |  |  |  |  |  |  | |  | |  | |  | |  |
| 743 | *Ailanthus altissima* (Mill.) Swingle | Ramban | Upto 2100 | T | Lf |  | Fodder | 0.10 | 0.30 | | 0.30 | | 0.0050 | | 0.33 | |  |
| 744 | *Brucea javanica* (L.) Merr | Hala | 600-1200 | T | Fr, Bk | Least Concern | chutney (Sauce) | 0.10 | 0.30 | | 0.30 | | 0.0050 | | 0.33 | |  |
|  | **Smilacaceae** |  |  |  |  |  |  |  |  | |  | |  | |  | |  |
| 745 | *Smilax aspera* L. | Kukardara | 1100-1600 | S | Rt |  | Medicinal (Skin eruptions, sores, wounds, diuretic, diaphoretic, rheumatic-arthritis); Fuel | 0.70 | 1.20 | | 0.28 | | 0.0373 | | 1.17 | |  |
|  | **Solanaceae** |  |  |  |  |  |  |  |  | |  | |  | |  | |  |
| 746 | *Atropa acuminata* Royle ex Miers | Belladona/ Jharka | 2500-3000 | H | Lf , Rt |  | Medicinal (Antidote, sedative, narcotic, anodyne); Fodder | 0.50 | 0.30 | | 0.04 | | 0.0013 | | 0.42 | |  |
| 747 | *Datura innoxia* Mill. |  | 800-1600 | H | Lf, Sd, Fr |  | Medicinal (Antihydrophobic, boils, sedative, asthma, anodyne, skin diseases, boils, rheumatism, parkinson's disease, excess causes giddiness, dry mouth, hallucinations and coma, fistulas, abscesses wounds and severe neuralgia) | 1.40 | 0.60 | | 0.50 | | 0.0167 | | 0.58 | |  |
| 748 | *Datura stramonium* L. | Datura | 1100-1600 | H | Lf, Sd, Fr |  | Medicinal (Asthma, dislocation of joints, jaundice, rheumatism, stomach complaints, toothache, veterinary lactation) | 0.70 | 0.60 | | 0.50 | | 0.0167 | | 0.58 | |  |
| 749 | *Hyoscyamus niger* L. | Khurasani Ajwain | 1600-3000 | H | Sd, Lf , Fl, Bk |  | Medicinal (Astringent, hysteria, muscular pain, sedative, toothache, whooping cough) | 0.60 | 0.30 | | 0.04 | | 0.0013 | | 0.42 | |  |
| 750 | *Nicotiana tabacum* L. |  | 800-1800 | H | Wp |  | Medicinal (Eczema, antispasmodic, asthma, itching, toothache, wounds, antihelminthic) | 0.70 | 0.60 | | 0.40 | | 0.0133 | | 0.58 | |  |
| 751 | *Physaiis minima* L. |  | 1000-1500 | H | Wp |  | Medicinal (Abdomen disease, earache, fever, gastric disease, stomachache); Edible | 0.60 | 0.30 | | 0.30 | | 0.0050 | | 0.33 | |  |
| 752 | *Physochlaina praealta* (Decne) Miers. |  | 3500–4600 | H | Wp, Sd, Lf |  | Medicinal (Epilepsy, liver complaints, boils, ulcers) | 0.40 | 0.30 | | 0.20 | | 0.0033 | | 0.33 | |  |
| 753 | *Solanum americanum* Mill. |  | 1400-2500 | H | Fr, Lf, Fl, Sd |  | Medicinal (Boils, cough, skin diseases) | 0.30 | 0.30 | | 0.30 | | 0.0050 | | 0.33 | |  |
| 754 | *Solanum indicum* L. |  | 800-1800 | H | Fr |  | Medicinal (Asthma, fever, colic) | 0.30 | 0.60 | | 0.50 | | 0.0167 | | 0.58 | |  |
| 755 | *Solanum nigrum* L. | Budhi ki cheer, Makoy | 800-3000 | H | Lf , Fr, Wp |  | Medicinal (Antidote to opium toxic, Headache, boils, cough, dysentery, ear complaints, fever, eye complaints, skin diseases, urinary complaints, piles, rheumatism, sprain, stomachache, swell, throat trouble, ulcer in mouth); Edible | 1.80 | 0.90 | | 0.16 | | 0.0160 | | 0.92 | |  |
| 756 | *Solanum viarum* Dunal | Jungali bhindi | 900-2600 | H | Fr, Sd |  | Medicinal (Respiratory disorders of goats) | 0.10 | 0.30 | | 0.30 | | 0.0050 | | 0.33 | |  |
| 757 | *Withania somnifera* (L.) Dun. | Ashvagandha, Ganda | 1100-1500 | S | Rt |  | Medicinal (Tumors, carbuncles, ulcers, leucoderrna, bronchitis, asthma, tonic, increases sperm count, sexual potency, increases the iron content in the blood, curing AIDS, weakness in children and old age peoples, cures pain in backache, weak eyesight, arthritis, vitality, antioxidant, diabetes, anti-inflammatory, immune-modulating, anti stres s, aphrodisiac, anthelmintic, psoriasis, bronchitis, ulcers, scabies, marasmus ofchildren, insomnia, debility, lumbago, asthma, boils, bronchitis, cough ,dropsy, eye complains, wounds, skin diseases, swelling of hands, feets, thirst); Edible; Misc. (Leaves are mixed with tea used to prepare tea) | 4.40 | 0.90 | | 0.34 | | 0.0504 | | 1.00 | |  |
|  | **Sterculiaceae** |  |  |  |  |  |  |  |  | |  | |  | |  | |  |
| 758 | *Helicteres isora* L. |  | 700-1000 | Sh | Ap, St |  | Medicinal (Diarrhoea, antispasmodic); Fibre | 0.30 | 0.30 | | 0.30 | | 0.0050 | | 0.33 | |  |
| 759 | *Melochia corchorifolia* L. |  | 700-1000 | Sh | Lf |  | Edible | 0.10 | 0.30 | | 0.30 | | 0.0050 | | 0.33 | |  |
|  | **Symplocaceae** |  |  |  |  |  |  |  |  | |  | |  | |  | |  |
| 760 | *Symplocos chinensis* (Lour.) Decne | Lash, Lojj | 1000–2800 | T | Bk, Lf |  | Medicinal (Astringent, astringent, diarrhoea); Fuel; Agricultural tools | 0.50 | 0.90 | | 0.05 | | 0.0072 | | 1.00 | |  |
| 761 | *Symplocos paniculata* (Thunb.) Miq. | Lodge | 1500-2000 |  |  |  | Medicinal (Menorrhagia, bowel complaints, , sprains and muscular swellings, eye diseases and ulcers) | 0.50 | 0.30 | | 0.30 | | 0.0050 | | 0.33 | |  |
|  | **Taxaceae** |  |  |  |  |  |  |  |  | |  | |  | |  | |  |
| 762 | *Taxus wallichiana* Zucc. | Birmi/Yew leaves/Rakhal/Nagdaun/Brahmi | 1800-3400 | T | Lf , Fr, Bk |  | Medicinal (Anticancerous, blood purifier, swelling, asthma, contraceptive); Flavouring tea. | 0.60 | 0.90 | | 0.16 | | 0.0160 | | 0.92 | |  |
|  | **Teliaceae** |  |  |  |  |  |  |  |  | |  | |  | |  | |  |
| 763 | *Grewia asiatica* L. |  | 700-1100 | T | Fr, Bk, Lf |  | Medicinal (Astringent, cooling, demulcent, rheumatism, antibacterial, rich in Vitamin A and C) | 0.60 | 0.30 | | 0.30 | | 0.0050 | | 0.33 | |  |
| 764 | *Grewia oppositifolia* Buch.-Ham. ex D.Don | Bihul | 800-1600 | T | Lf, St, Fr, Bk |  | Medicinal (Child birth, stomachache); Edible; Fodder; Agricultural Tools; Misc. (Making ropes) joint pains | 0.60 | 0.60 | | 0.024 | | 0.0032 | | 0.83 | |  |
| 765 | *Grewia optiva* Drummond ex Burret | Dhaman |  | T | Lf |  | Medicinal (Joint pains, child bith, stomachache); Edible; Agricultural tools; Making roops | 0.30 | 0.60 | | 0.012 | | 0.0016 | | 0.83 | |  |
| 766 | *Triumfetta rhomboidea* Jacq. |  | 700-1200 | Sh | Wp, Fiber |  | Medicinal (Abortifacient, childbirth, stomachache); Fodder | 0.40 | 0.30 | | 0.04 | | 0.0013 | | 0.42 | |  |
|  | **Thymeleaceae** |  |  |  |  |  |  |  |  | |  | |  | |  | |  |
| 767 | *Daphne papyracea* Wall. ex G. Don | Nera | 1400–2300 | S | Rt, Lf |  | Medicinal (Intestinal complaints, hypotensive, purgative, fever, cough, cold, intestinal complaints) | 0.70 | 1.20 | | 0.80 | | 0.0533 | | 1.08 | |  |
| 768 | *Wikstroemia canescens* Meisn. | Bhojishel | 1800-3000 | S | Lf, Br |  | Pisticicidal | 0.10 | 0.30 | | 0.30 | | 0.0050 | | 0.33 | |  |
|  | **Trillidiaceae** |  |  |  |  |  |  |  |  | |  | |  | |  | |  |
| 769 | *Trillium govanianum* Wall. | Satwa | 2500-3800 | H | Tu |  | Medicinal (Dysentery, arthritis) | 0.20 | 0.90 | | 0.60 | | 0.0300 | | 0.83 | |  |
|  | **Ulmaceae** |  |  |  |  |  |  |  |  | |  | |  | |  | |  |
| 770 | *Celtis australis* DC. | Kharik | 1100-1800 | T | WP, Lf, Rt, Bk |  | Medicinal (Menorrhea, colic pain, leprosy, Bone fracture, pimples contusions, sprains and joint pain); Fodder; Fuel | 0.80 | 0.60 | | 0.50 | | 0.0167 | | 0.58 | |  |
| 771 | *Ulmus villosa* Brandis ex Gamble | Chor | 1500-2000 | T | Lf, Wd |  | Fodder; Fuel | 0.20 | 0.30 | | 0.04 | | 0.0013 | | 0.42 | |  |
| 772 | *Ulmus wallichiana* Planch. |  | 2000–3000 | T | Bk |  | Medicinal (Bone fracture, dislocation of joints) | 0.20 | 0.30 | | 0.30 | | 0.0050 | | 0.33 | |  |
|  | **Urticaceae** |  |  |  |  |  |  |  |  | |  | |  | |  | |  |
| 773 | *Boehmeria platyphylla* Jacq. | Siar | 1200-1500 | S | Lf, St, Br |  | Medicinal (Dysentery, eczema) | 0.20 | 0.60 | | 0.50 | | 0.0167 | | 0.58 | |  |
| 774 | *Debregeasia longifolia (Burm. f.) Wedd.* | Shyaru | 1400-2000 | Sh | Bk, Lf |  | Fodder; Fibre; Fuel | 0.30 | 0.30 | | 0.30 | | 0.0150 | | 0.50 | |  |
| 775 | *Debregeasia salicifolia* (D.Don.) Rendle. |  | 1500-2000 | Sh | Bk, Lf |  | Fodder; Fibre; Fuel | 0.30 | 0.30 | | 0.01 | | 0.0006 | | 0.50 | |  |
| 776 | *Girardinia diversifolia* (Link) Friis | Bichhubutti, Jarahan | 2000-3000 | H | Lf , Twing, Rt, Br |  | Medicinal (Cuts, wound, urinary disease, gonorrhoea, bone fracture, muscle cramp) Fibre | 0.60 | 0.90 | | 0.70 | | 0.0350 | | 0.83 | |  |
| 777 | *Parietaria debilis* Frost. |  | 800-2000 | H | Rt |  | Medicinal (Dandruff, hair problems) | 0.20 | 0.60 | | 0.60 | | 0.0200 | | 0.58 | |  |
| 778 | *Parietaria umbrosa* Blume |  | 1800-2600 | H | Lf |  | Medicinal (Wounds) | 0.10 | 0.30 | | 0.30 | | 0.0050 | | 0.33 | |  |
| 779 | *Urtica dioica* L. | Ain, Kungsh, aahan | 3000-4500 | S | Lf , Wp |  | Medicinal (Anthelmitic, antiseptic oils, wounds, dandruff, diarrhea, gout, rheumatism, sciatica, jaundice, nephritis, sprain, throat diseases, swelling, injuries, blood purifier, jaundice, skin eruption, astringent, dog bite wounds, kidney disease, postnatal tonic, throat pain, tonic); Edible | 2.40 | 1.20 | | 0.30 | | 0.0400 | | 1.17 | |  |
| 780 | *Urtica hyperborea* D. Don |  | 3000-4500 | S | Wp |  | Medicinal (Rheumatism, stomachache) | 0.20 | 0.60 | | 0.50 | | 0.0167 | | 0.58 | |  |
|  | **Valerianaceae** |  |  |  |  |  |  |  |  | |  | |  | |  | |  |
| 781 | *Nardostachys grandiflora* DC. | Jatamansi | 3000–4000 | H | Rt |  | Medicinal (Blood purifier, cooling, cough, diarrhoea, tonic, ulcer, snakebite) | 0.70 | 0.60 | | 0.50 | | 0.0167 | | 0.58 | |  |
| 782 | *Nardostachys jatamansi* (D.Don) DC. | Nihani | 3500-5000 | H | Rf, Rt | Critically Endangered | Medicinal (Bitter tonic, stimulant, antipyretic, antispasmodic, antiseptic, anti-lipid, anti-malarial, anti-rhytmic, sedative, antidepressant, laxative, stomachic, memory, blood purifier, cooling, cough, diuretic, tonic, ulcer, snake bite) | 2.00 | 1.50 | | 1.30 | | 0.1083 | | 1.33 | |  |
|  | **Verbenaceae** |  |  |  |  |  |  |  |  | |  | |  | |  | |  |
| 783 | *Callicarpa macrophylla* Vahl | Nagdhava | 700-2000 | Sh | Lf, Rt |  | Medicinal (Cough, cold, relieves stomach ailments, rheumatism, indigestion, ulcers, diuretic) | 0.70 | 0.30 | | 0.30 | | 0.0050 | | 0.33 | |  |
| 784 | *Caryopteris foetida* (D.Don) Thell. | Rumri | 1500-2000 | Sh | Lf |  | Medicinal (Wounds) | 0.10 | 0.30 | | 0.30 | | 0.0050 | | 0.33 | |  |
| 785 | *Lantana camara* L. |  | 700-1700 | Sh | Lf, Fr |  | Medicinal (Itching, malaria, rheumatism, ringworm); Edible; Fuel | 0.60 | 0.30 | | 0.04 | | 0.0013 | | 0.42 | |  |
| 786 | *Verbena officinalis* L. | Ach | 2000-2500 | H | Fr, Rt |  | Medicinal (Ear drops, snake bite, stomachache, rheumatism and joint problems, nerve tonic, nervous disorders, epilepsy, some respiritory problems such as whooping cough, urinary tract problems, sedetive, detoxification, mild fever, sore throats, skin complaints, bruising, eye tonic) | 1.60 | 0.30 | | 0.30 | | 0.0050 | | 0.33 | |  |
| 787 | *Vitex negundo* L. | Bana | 100-1300 | S | Lf , Wp |  | Medicinal (Blister, bone fracture, bodyache, cold, colic, diarrohea, epilepsy, fever, gout, gum trouble, headache, itch, mental disturbance, piles, joint pain, reduce sex desire, skin problem, tonic, ulcer); Insect repellent; Religious | 2.10 | 0.60 | | 0.15 | | 0.0150 | | 0.75 | |  |
|  | **Violaceae** |  |  |  |  |  |  |  |  | |  | |  | |  | |  |
| 788 | *Viola biflora* L. | Banafsha | 2100-4500 | H | Lf, Fl, Sd |  | Medicinal (Sore throa, Antiseptic, antispasmodic, cold, cough, diaphoretic, laxative, leucoderma, skin disease) | 0.90 | 0.60 | | 0.50 | | 0.0167 | | 0.58 | |  |
| 789 | *Viola canescens* Wall. | Banafsha | 1400-2600 | H | Lf, Fl, Sd |  | Medicinal (Asthma, bronchitis, cold, cough, eye diseases, stomachache, malarial, emetic, demulcent, cuts, wound) | 1.10 | 1.20 | | 0.70 | | 0.0467 | | 1.08 | |  |
| 790 | *Viola pilosa* Blume | Vanaksa | 1200-3000 | H | Fls, Wp |  | Medicinal (Fever, cough, cold) | 0.30 | 0.30 | | 0.30 | | 0.0050 | | 0.33 | |  |
| 791 | *Viola serpens*Wall | Banafsha | 2100-4500 | H | Fl, Lf , Sd |  | Medicinal (Asthma, bronchitis, cold, cough, wounds, eye disorder, laxative, leucoderma) | 0.80 | 0.60 | | 0.50 | | 0.0167 | | 0.58 | |  |
|  | **Vitaceae** |  |  |  |  |  |  |  |  | |  | |  | |  | |  |
| 792 | *Ampelocissus latablia* (Vahl) Plach. |  | 700-1000 | Sh | Rt |  | Medicinal (Bone fracture, dysentery, fever, pain in stomach, pneumonia, snake bite) | 0.50 | 0.30 | | 0.30 | | 0.0050 | | 0.33 | |  |
| 793 | *Cissus repanda* Vahl. |  | 1400-1800 | S | Lf |  | Medicinal (Wounds) | 0.10 | 0.30 | | 0.30 | | 0.0050 | | 0.33 | |  |
| 794 | *Parthenocissus hitnalayana* Planch. |  | 1200-2000 | H | Fr |  | Edible; Fodder | 0.20 | 0.30 | | 0.03 | | 0.0010 | | 0.42 | |  |
| 795 | *Parthenocissus semicordata* (Wall.) Planchon | Amru bail | 1800-3300 | Climber | Ap & Rt |  | Medicinal (Leucorrhoea, wounds, boils); Edible; Fodder | 0.30 | 0.30 | | 0.30 | | 0.0150 | | 0.50 | |  |
|  | **Woodsiaceae** |  |  |  |  |  |  |  |  | |  | |  | |  | |  |
| 796 | *Hypodematium crenatum* (Forssk.) | Budhi kebaal | 1800-3300 | Fern | Rf |  | Medicinal (Antibacterial, conception) | 0.20 | 0.30 | | 0.30 | | 0.0050 | | 0.33 | |  |
|  | **Zingiberaceae** |  |  |  |  |  |  |  |  | |  | |  | |  | |  |
| 797 | *Alpinia calcarata* (Haw.) Roscoe | JungliElaayachi | 800-1300 | H | Rh |  | Medicinal (Bronchitis, dyspepsia) | 0.20 | 0.30 | | 0.30 | | 0.0050 | | 0.33 | |  |
| 798 | *Alpinia zerumbet* (Pers.) B.L.Burtt & R.M.Sm. | JungliAdra | 800-1300 | H | Rh |  | Medicinal (Stimulant, anti- bactieria) | 0.20 | 0.30 | | 0.30 | | 0.0050 | | 0.33 | |  |
| 799 | *Amomum subulatum* Roxb |  |  | H | Sd | Data deficient | Edible | 0.10 | 0.30 | | 0.04 | | 0.0013 | | 0.42 | |  |
| 800 | *Cheilocostus speciosus* (J.Koenig) C.D.Specht | Kide ki chali | 900-1800 | H | Rh,St |  | Medicinal (Dysentery, fever, asthma, intestinal worms, purgative) | 0.20 | 0.30 | | 0.04 | | 0.0007 | | 0.33 | |  |
| 801 | *Curcuma angustifolia* Roxb. | Ban Haldi, Chudidar Haldi | 800-1300 | H | Rf |  | Medicinal (Bone fracture, diarrohea, fever, jaundice, swellings, joint pains, eye pain, urinary inflammation) | 0.80 | 0.60 | | 0.60 | | 0.0200 | | 0.58 | |  |
| 802 | *Globba marantina* L. | Banse | 800-1700 | S | Lf |  | Medicinal (Sores) | 0.10 | 0.30 | | 0.30 | | 0.0050 | | 0.33 | |  |
| 803 | *Globba racemosa* Sm. |  | 800-1700 | H | Rt |  | Medicinal (Headache) | 0.10 | 0.30 | | 0.20 | | 0.0033 | | 0.33 | |  |
| 804 | *Hedychium acuminatum* Roscoe | Kapur Kachri/ Kachur/ Van Haldi | 800-2800 | H | Rf, Rt |  | Medicinal (Insect repellant, aromatic, anti-arthritic, apptiser, cardiac, stimulant, carminative, hair tonic, asthma, diarrhoea, dysentery, headach, hairfall, skin diseases, and vomiting); Incense; Dye | 1.70 | 0.30 | | 0.03 | | 0.0014 | | 0.50 | |  |
| 805 | *Hedychium spicatum* (Ham-ex-Smith) | Vanhaldi | 1000-2500 | H | Rf |  | Medicinal (Asthma, blood purifier, bronchitis, nausea); Fodder, Misc. (Mats) | 0.60 | 1.20 | | 0.08 | | 0.0160 | | 1.25 | |  |
| 806 | *Roscoea alpina* Royle |  | 200–3500 | H | Rt |  | Medicinal (Wounds, cuts of cattle, tonic) | 0.30 | 0.30 | | 0.20 | | 0.0033 | | 0.33 | |  |
| 807 | *Roscoea purpurea* Sm. |  | 1500–3000 | H | Rt |  | Medicinal (Wounds, cuts of cattle, tonic, veterinary) | 0.40 | 0.30 | | 0.20 | | 0.0033 | | 0.33 | |  |
|  | **Zygophyllaceae** |  |  |  |  |  |  |  |  | |  | |  | |  | |  |
| 808 | *Equisetum arvense* L. |  | 1800-3050 | H | WP |  | Medicinal (Healing) | 0.10 | 0.30 | | 0.30 | | 0.0050 | | 0.33 | |  |
| 809 | *Tribulus terrestis* L. | Gokharu | 1100-1600 | H | WP |  | Medicinal (Urinary diseases in painful micturition, suppression of urine, cough, asthma) | 0.40 | 0.30 | | 0.30 | | 0.0050 | | 0.33 | |  |
| 810 | **Lichens** | Chalora/Chharila/Chreda phool mendhi |  |  |  |  | Medicinal (Antibiotics, Preservatives, and Toxins); Edible;  Spices; Dyes; Ingredients in Perfumes and Deodorants | 0.70 | 0.30 | | 0.05 | | 0.0040 | | 0.67 | |  |
| 811 | **Mosses** | Green Moss Ghass |  |  |  |  | Medicinal (Hepatic disorders, skin diseases, cardiovascular diseases, asantipyretic, antimicrobial, wound healing) | 0.60 | 0.30 | | 0.40 | | 0.0067 | | 0.33 | |  |
