# Supplementary material for: Richness of non-timber forest products in Himalayan communities—diversity, distribution, use pattern and conservation status
Source: J Ethnobiol Ethnomed. 2020 Sep 23;16:56. doi: 10.1186/s13002-020-00405-0 (PMC7513279; doi:10.1186/s13002-020-00405-0)
Supplement: Supplementary file 2 — Additional file 2. List of Source of literature (references) used in this study for synthesizing information. [file 13002_2020_405_MOESM2_ESM.docx]

**List of Source of literature (references) used in this study for synthesizing information**

1. Ahuja PS. Medicinal Plants in India: Report and Directory. Institute of Economic and Market Research, New Delhi, 2003.
2. Anonymous. Appendix I, II and III to Convention on International Trade in Endangered Species of Wild Fauna and Flora. Arlington VA: US Fish & Wildlife Services; 2001:32 pp.
3. Anonymous. Conservation Assessment and Management Plan Workshop Process. WWF, India; 1997
4. Anonymous. Wealth of India, Raw Materials Vol. 1- 11. Publication and Information Directorate, CSIR, New Delhi, 1945-1976.
5. Arora A, Sharma M, Gupta S. Ethnomedicinal studies of some tree species of Chamba district (Himachal Pradesh), India. *Annals of Plant Sciences*, 2018; 2056–2062.
6. Arora RK, Pandey A. Wild edible plants of India: Diversity, conservation and use; 1996. Available at https://www.researchgate.net/publication/236278305
7. Aryal KP, Poudel S, Chaudhary RP, Chettri N, Chaudhary P, Ning W, et al. Diversity and use of wild and non-cultivated edible plants in the Western Himalaya. J Ethnobiol Ethnomedicine. 2018;14:10.
8. Aswal BS, Mehrotra BN. Flora of Lahaul-Spiti (A cold desert in North West Himalaya). Bisen Singh Mahendra Pal Singh, Dehradun, India, 2009, 761 p.
9. Aswal BS, Mehrotra BN. Flora of Lahaul-Spiti. (A Cold Desert in Northwest Himalayas). Dehradun: Bishen Singh Mahendra Pal Singh; 1994
10. Atkinson ET. Economic Botany of the Himalayan Region. Cosmo Publications, New Delhi, 1882.
11. Badola HK, Pal M. Threatened medicinal plants and their conservation in Himachal Himalaya. Ind For 2003;129:55–68
12. Badola HK. Biodiversity Conservation Study of Kanawar wildlife sanctuary in Himachal Pradesh, In: Research for Mountain Development: Some Initiatives and Accomplishments, Gyanadoya Prakashan, Nanital; 1998; 407-430
13. Badola HK. Medicinal plant diversity of Himachal Pradesh. In: Samant SS, Dhar U and Palni LMS (eds), Himalayan Medicinal Plants: Potential and Prospects. Nainital: Gyanodaya Prakashan; 2001; 87–116
14. Balokhra JM. The Wonderland Himachal Pradesh. H.G. Publications, New Delhi; 1995.
15. Balokhra JM. The Wonderland–Himachal Pradesh. H.G. publications, New Delhi, 1995, 718 p.
16. Bhatia H, Sharma YP, Manhas RK, Kumar K. Ethnomedicinal plants used by the villagers of district Udhampur, J&K. India. Journal of Ethnopharmacology. 2014;151(2):1005–1018.
17. Bhattacharya UC, Uniyal BP. A Botanical tour to Pangi and Trilokinath in upper Chenab. Journal Bombay Natural History Society 1982;79:57-78
18. Boktapa NR, Sharma AK. Wild Medicinal Plants used by local communities of Manali, Himachal Pradesh, India. Ethno Botanical Leaflets. 2010; 14:259-67.
19. Butola JS, Badola HK. Threatened Himalayan Medicinal plants and Their Conservation in Himachal Pradesh. J. Trop. Med. Plants. 2008; 9(1):125-142..
20. Butola JS, Vashistha RK. An overview on conservation and utilization of Angelica glauca Edgew. in three Himalayan states of India. Med Plants. 2013;5:171–177.
21. Chauhan NS. (). Important medicinal and aromatic plants of Himachal Pradesh. Indian Forester. 2003;129(8):979–998.
22. Chauhan NS. Ethnobotanical Study of medicinal plants of Himachal Pradesh. In: Kaushik P (ed.), Indigenous Medicinal Plants. New Delhi: M/s Today & Tomorrows Printers & Publishers; 1988;187–98
23. Chauhan NS. Medicinal and Aromatic Plants of Himachal Pradesh, Indus Publishing Company, New Delhi, 1999; 500
24. Chauhan NS. Medicinal and aromatic plants of Himachal Pradesh, Indus Publishing Co., New Delhi, 1999.
25. Chauhan NS. Medicinal orchids of Himachal Pradesh. Journal of Orchids Society of India. 1990; 4:99–105
26. Chauhan NS. Plant resources of economic use in Himachal Pradesh. Solan: DEE-UH & F; 1996:77 pp.
27. Chauhan NS. Potential of aromatic plants flora in Himachal Pradesh. Indian Perfumes 1989;33(2):118–22
28. Chauhan PP, Nigam A, Santvan VK. Ethnobotanical study of wild fruits in Pabbar Valley, District Shimla, Himachal Pradesh. J. Medic. Plants Stud. 2016; 4(2):216-220.
29. Chauhan, NS, Khosla PK. Commercially important medicinal plants of Himachal Pradesh In: PK Khosla (Ed) Tends in Tree Sciences ISTS Publications 1988; 81-89.
30. Chopra RN, Nayar SL, Chopra IC. Glossary of Indian medicinal plants. CSIR, New Delhi. 1956; 330 p.
31. Chowdhery HJ, Wadhwa BM. Flora of Himachal Pradesh, Vols. 1–3. Culcutta: Botanical Survey of India; 1984
32. Chowdhury AR. (). GC/MS studies of volatiles from *Artemisia vestita* aerial parts. Journal of Essential Oil-Bearing Plants. 2003;6:210–213.
33. Collett H. Flora Simlensis. Thacker Spink and Co., London (Reprinted 1971). Bishen Singh Mahendra Pal Singh, Dehra Dun, India, 1902, 652 p.
34. Dhaliwal DS, Sharma M. Flora of Kullu District (Himachal Pradesh). Bishen Singh Mahendra Pal Singh Dehradun. 1999
35. Dhaliwal DS,Sharma M. Phytogeographic comments on the flora of Kullu district. In Sharma TA, Saini SS, Trivedi ML and Sharma M (eds), Current researches in plant sciences Vols. I–II. Bishen Singh Mahendra Pal Singh Dehradun. 1997;1:169–75
36. Dhar U and Samant SS. Endemic diversity of Indian Himalaya. I. Ranunculaceae and II. Paeoniaceae. Journal of Biogeography. 1993;20:659–68
37. Dhar U, Manjkhola S, Joshi M, Bhatt ID, Bisht AK and Joshi M. Current status and future strategy for development of medicinal plants sector in Uttaranchal, India. Current Science. 2002;83(8):56–64
38. Dhar U, Rawal, RS and Upreti J. Setting prioritization for conservation of medicinal plants: a case study in the Indian Himalaya. Biological Conservation 2000;95:57–65
39. Dutt B, Nath S, Chauhan NS, Sharma KR, Sharma SS. Ethno-medicinal plant resources of Tribal of Pangi valley in district Chamba, Himachal Pradesh, India. Inter Jour of Bio-resource and Stress Management. 2014;5(3), 416–421.
40. Gammie GA. A botanical tour in Chamba and Kangra. Records of Botanical Survey of India 1898; I;183–214
41. Gaurav, Thakur P, Kumar K. Mehta P. Farmers' perceptions on production and marketing of medicinal and aromatic plants in Kullu District of Himachal Pradesh-India. Int. J. Pure App. Biosci. 2018;6(3):665-675.
42. Gautam AK, Bhadauria R. Homeopathic Flora of Bilaspur District of Himachal Pradesh, India: A Preliminary Survey. Ethnobotanical Leaflets. 2009;13:123-30.
43. Gouri, Mudgal S, Morrison E, Mayers J.. Policy influences on forest-based livelihoods in Himachal Pradesh, India. International Institute for Environment and Development, London. 2004; 115 p.
44. Gulati AK, Pandey S and Gupta S. A Guide to National Parks and Wildlife Sanctuaries of Himachal Pradesh. Himachal Pradesh: Wildlife Wing, Forest Department; 2004
45. Guleria V, Vasishth A. Ethnobotanical uses of wild medicinal plants by Guddi and Gujjar tribes of Himachal Pradesh. Ethnobotanical Leaflets. 2009;13:1158-1167.
46. Gupta R. Medicinal and aromatic plants of Bhandal ranges, Churah forest division, Himachal Pradesh. Journal of Bombay Natural History Society.1971;68:791–803.
47. Gupta R. Survey record of medicinal and aromatic plants of Chamba Forest Division of H.P. Indian Forester. 1964; 90: 454-468.
48. Jain SK. Dictionary of Indian Folk Medicine and Ethnobotany. Deep Publications, New Delhi, 1991.
49. Jain SK. Dictionary of Indian Folk Medicine and ethnobotany. New Delhi: Deep Publications; 1991
50. Joshi SG. Medicinal plants in India. Oxford and IBH Publishing Co. Pvt. Ltd., New Delhi, 2000; 491 p.
51. Kala CP, Mathur VB. Patterns of plant species distribution in the Trans-Himalayan region of Ladakh, India. J Veg Sci. 2002;13:751–754.
52. Kala CP, Sajwan SB. Revitalizing Indian systems of herbal medicine by the National Medicinal Plant Board through institutional networking and capacity building. Current Science 2007;93(6): 797–806
53. Kala CP. Medicinal Plants of the high altitude cold desert in India: diversity, distribution & traditional uses. International Journal of Biodiversity Science & Management 2006a;2(1):43–56
54. Kala CP. Medicinal plants: potential for economic development in the State of Uttaranchal, India. International Journal of Sustainable Development & World Ecology 2006b;13(6):492–8
55. Kala CP. Status and conservation of rare and endangered medicinal plants in the Indian trans-Himalaya. Biol Conserv. 2000;93:371–379.
56. Kala, C.P. Status and conservation of rare and endangered medicinal plants in the Indian trans-Himalaya. Biological Conservation, 2000;93(3):371-379.
57. Kapahi BK. Ethnobotanical investigations in Lahaul (Himachal Pradesh). Journal of Economic & Taxonomic Botany 1990;14:49–55
58. Kapur SK. Traditionally important medicinal plants of Bhaderwah hills - Jammu Province III. J. Econ. Tax. Bot., Addl. Series. 1996;12:62-69.
59. Kaur H, Sharma M. Flora of Sirmaour Himachal Pradesh. Bishen Singh Mahendra Pal Singh, Dehradun, 2004; 770 p.
60. Kaur I, Sharma S, Lal S. Ethnobotanical survey of medicinal plants used for different diseases in Mandi district of Himachal Pradesh. International Journal of Research in Pharmacy and Chemistry, 2011;1(4):1167-1171.
61. Khare CP. Encyclopedia of Indian medicinal plants. Springer Verlag, Berlin, Heidelberg, New York, 2004; 523.
62. Khare CP. Indian Medicinal Plants: An Illustrated Dictionary. Springer Science+Business Media, LLC, 2007.
63. Kirtikar KR, Basu BD. Indian medicinal plants, 2nd edn., 1988; pp. 1162–1163.
64. Kishor A, Kumar A, Tomer V, Kumar V, Gupta K.. Wild food plants of Himachal Pradesh: a review. Plant Archives. 2018;18(2):2737-2751.
65. Kumar N, Jakhar AK, Choyal RR. Ethno Medicinal uses of some plants of Hamirpur district of Himachal Pradesh for The treatment of piles. Life Sciences Leaflets. 2014; 50:1-10.
66. Kumar S, Chand G, Sankhyan P. Herbal folk remedies for curing various ailments in Lug valley of district Kullu, Himachal Pradesh (NW Himalaya). International Journal of Ayurvedic and Herbal Medicine. 2013a; 3(5):1308-1314.
67. Kumar M, Bandu Sharma. Commonly used medicinal plants in Tehsil Baijnath, district Kangra, Himachal Pradesh, India; Research in Pharmacy; 2014;4(2):11-15
68. Kumar, S. 2019. Wild edible plants consumed by rural communities in district Bilaspur, Himachal Pradesh, India. J. Biol. Chem. Chron. 2019;5(2):2454-2476.
69. Kumari P, Samant SS, Puri S. Diversity, distribution, indigenous uses and conservation of medicinal plants in central Himachal Pradesh, North Western Himalaya. J Med Plants. 2018;6:45–68.
70. Kumari S, Batish DR, Singh HP, Negi K, Kohli RK. An ethnobotanical survey of medicinal plants used by Gujjar Community of Trikuta Hills in Jammu and Kashmir. Indian Journal of Medicinal Plants Research. 2013:7(28):2111–2121.
71. Kuniyal CP, Rawat YS, Oinam SS, Kuniyal JC, Vishvakarma SC. Kuth (*Saussurea lappa*) cultivation in the cold desert environment of the Lahaul valley, northwestern Himalaya, India: arising threats and need to revive socio-economic values. Biodivers Conserv. 2005;14:1035–1045.
72. Lal B, Singh KN. Indigenous herbal remedies used to cure skin disorders by the natives of Lahaul-Spiti in Himachal Pradesh. Indian Journal of Traditional Knowledge. 2008;7(2):237-241.
73. Maheshwari JK. Ethnobotany and medicinal plants of Indian subcontinent. Scientific Publishers, Jodhpur, India, 2000; 672.
74. Man SR, Samant SS. Diversity, indigenous uses and conservation status of medicinal plants in Manali wildlife sanctuary, North Western Himalaya. Indian Journal of Traditional Knowledge. 2011;10(3):439-459.
75. Mishra ML, Sood S, Shukla UN. Phyto-nutritional and mineral composition of Indian Horse Chestnut (*Aesculus indica*) seeds. J. Pharmacogn Phytochem. 2018;7(1):2159-2162.
76. Nair NC. Flora of Bashahr Himalayas. International Biosciences Publishers, Hissar, Madras, India, 1977; 360.
77. Nayar MP. Sastry ARK. Red Data Book of Indian Plants, Vol. I–III. Calcutta: Botanical Survey of India; 1987, 1988, 1990.
78. Negi PS, Subramani SP. Wild edible plant genetic resources for sustainable food security and livelihood of Kinnaur district, Himachal Pradesh, India. Int J Conserv Sci. 2015;6(4):657-658.
79. Radha, Puri S, Kumar S. Diversity and use of wild edible plants by migratory shepherds in the Himachal Pradesh of the Western Himalayas, India. J Med Pl Res. 2018;12(30):601-610.
80. Rana D, Bhatt A, Lal B, Parkash O, Kumar A, Uniyal SK. Use of medicinal plants for treating different ailments by the indigenous people of Churah subdivision of district Chamba, Himachal Pradesh, India. Environ Dev Sustain. 2020;1–80.
81. Rana D, Bhatt A, Lal B. Ethnobotanical knowledge among the semi-pastoral Gujjar tribe in the high altitude (Adhwari’s) of Churah subdivision, district Chamba, Western Himalaya. J Ethnobiol Ethnomedicine. 2019;15:10.
82. Rani S, Chahota RK, Sharma TR. Ethnobotanical survey and traditional knowledge of some medicinal and aromatic plants of Himachal Pradesh, North-West Himalayas. International Journal of Applied and Pure Science and Agriculture.2015;1(12):1–22.
83. Rani S, Rana JC, Rana PK. Ethnomedicinal plants of Chamba district, Himachal Pradesh, India. J Med Pl Res. 2013; 7(42):3147-3157.
84. Rani S, Rana JC, Rana PK. Ethnomedicinal plants of Chamba district, Himachal Pradesh, India. J Med Pl Res. 2013;7(42):3147-3157.
85. Rathore S, Shashni S, Sharma A, Sundriyal RC. () Ethnomedicinal study on medicinal plants used by the tribal people of Lahaul and Spiti district, Himachal Pradesh , North West Himalaya. Indian Forester. 2019:145(12):1182-1189.
86. Rau MA. On a collection of plants from Lahaul. Bulletin Botanical Survey of India 1960;2:45–56
87. Rawat DS, Kharwal AD. Study on traditional herbal pediatrics practices in Jaisinghpur, district Kangra (Himachal Pradesh, India). Global Journal Research Medicinal Plants and Indigenous Medicine. 2013;2(4):219-230.
88. Samant SS and Pal M. Diversity and conservation status of medicinal plants in Uttaranchal State. Indian Forester 2003;129(9):1090–108
89. Samant SS, Butola JS and Sharma A. Assessment of diversity, distribution, conservation status and preparation of management plan for the medicinal plants in the catchment area of Parbati Hydro-Electric Project Stage III in North Western Himalaya. Journal of Mountain Science 2007;4(1): 34–56
90. Samant SS, Dhar U and Palni LMS. Himalayan Medicinal Plants: Potential and Prospects. Nainital: Gyanodaya Prakashan; 2001
91. Samant SS, Dhar U and Palni LMS. Medicinal Plants of Indian Himalaya: Diversity Distribution Potential Values. Nainital: Gyanodaya Prakashan;1998
92. Samant SS, Dhar U. Diversity, endemism and economic potential of wild edible plants of Indian Himalaya. International Journal of Sustainable Development & World Ecology 1997;4:179–91
93. Samant SS, Dhar U. Diversity, endemism and economic potential of wild edible plants of Indian Himalaya. Int. J. Sust. Dev. World. 1997;4(3):179-191.
94. Samant SS, Palni LMS. Diversity, distribution and indigenous uses of essential oil yielding medicinal plants of the Indian Himalayan region. Journal of Medicinal and Aromatic Plant Sciences, 2000;22:671-684.
95. Samant SS, Pant S. Diversity, distribution pattern and conservation status of plants used in liver diseases/ailments in Indian Himalayan Region. Journal of Mountain Science 2006;3(1):28–47
96. Samant SS, Pant S. Diversity, distribution pattern and traditional knowledge of Sacred Plants in Indian Himalayan Region. Indian Journal of Forestry 2003;26(3):201–13
97. Samant,SS, Pant S, Singh M, Lal M, Singh A, Sharma A, et al. Medicinal plants in Himachal Pradesh, North Western Himalaya, India. International Journal of Biodiversity Science and Management, 2007; 3:234-251.
98. Seth MK, Jaswal S. An enumeration of Plant Resources of Shimla (Himachal Pradesh) in the N.W. Himalayas. International Book Distributors & Publishers, Dehradun, 2004; 206.
99. Shabnam SR. Medicinal plants of Chamba. Indian Forester, 1964; 90:50–63.
100. Sharma B, Sharma S, Bhardwaj SK, Kimani C, Dutt B. Ethnobotanical uses of common plant species growing along the National Highway 5 from solan to Shimla in Himachal Pradesh. World Journal of Pharmacy and Pharmaceutical Sciences. 2015;4:1210-1218.
101. Sharma K, Sharma R, Nisha Devi. Non-Timber Forest Products (NTFPs) and livelihood security: an economic study of high hill temperate zone households of Himachal Pradesh. Economic Affairs. 2019; 64(2):305-315.
102. Sharma KR, Sood M. Important Medicinal and Aromatic Plants of Himachal Pradesh. Directorate of Extension Education and Department of Forest Products, Dr. YS Parmar University of Horticulture and Forestry, Nauni, Solan (H.P.), 2007.
103. Sharma M, Dhaliwal DS. Additions to the Flora of Himachal Pradesh from Kullu District. Journal of Bombay Natural History and Society 1997a;94(2):447–50
104. Sharma M, Dhaliwal DS. Biological Spectrum of the flora of Kullu district (Himachal Pradesh). J Ind Bot Soc 1997b;76:283–4
105. Sharma M, Singh H. Phytogeographic observations on the Flora of Chamba District (Himachal Pradesh) Part-II. Neo Botanica 1996;23:103-12
106. Sharma M, Sood SK. Ethnobotanical survey for wild plants of District Solan, Himachal Pradesh. India. International Journal of Environmental Biology, 2013;3(3):87-95.
107. Sharma P, Agnihotry A, Sharma PP, Sharma L. Wild edibles of Murari Devi and surrounding areas in Mandi district of Himachal Pradesh, India. International Journal of Biodiversity and Conservation 2013; 5(9):592-604.
108. Sharma P, Agnihotry A, Sharma PP, Sharma L. Wild edibles of Murari Devi and surrounding areas in Mandi district of Himachal Pradesh, India. International Journal of Biodiversity and Conservation, 2013;5:592–604.
109. Sharma PK, Chauhan NS, Lal B. Studies on plant associated indigenous knowledge among Malanis of Kullu district, Himachal Pradesh. Indian Journal of Traditional Knowledge. 2005; 4(4):403-408.
110. Sharma PK, Lal B. Ethnobotanical notes on some medicinal and aromatic plants of Himachal Pradesh. Indian Journal of Traditional Knowledge, 2005;4(4):424–428.
111. Sharma PK, Thakur SK, Manuja S, Rana RK, Kumar P, Sharma S, et al. Observations on traditional phytotherapy among the inhabitants of Lahaul valley through amchi system of medicine-A cold desert area of Himachal Pradesh in North Western Himalayas, India. Chinese Medicine, 2011; 2:93-102.
112. Sharma S, Gautam AK, Bhadauria R. Some important food plants and wild edible fungi of upper hilly region of district Shimla (Himachal Pradesh). Ethnobotanical Leaflet. 2009; 13:1020-1028.
113. Sharma S, Rana M. Commonly used medicinal plants in Tehsil Pachhad, district Simour, Himachal Pradesh. Pharma Tutor. 2016; 4(3):34-38.
114. Sharma S, Thakur A, Verma P, Kumari S, Sharma S, Arya V. Ethnomedicinal wisdom among local tribes in Hamirpur valley, Himachal Pradesh, India. Journal of Medical and Allied Sciences, 2012;2(2):88–94.
115. Shashni S, Rathore S, Sundriyal RC. Ethnomedicinal plants used for curing various Gynaecological Problems in North Western Himalayan District Kullu of Himachal Pradesh. Journal of non-timber forest products. 2019;26(2):1-5.
116. Shashni S, Sharma S, Rathore S, Samant SS, Sundriyal RC. Traditional uses and potential to develop an enterprise of wild rose species *Rosa brunoniisyn R. moschata* in Kullu district of North Western Himalaya. Journal of Non Timber Forest Products 2017; 24(3):137-139.
117. Singh DK, Hajra PK. Floristic diversity. In: Gujral GS, Sharma V eds. Biodiversity Status in the Himalaya. New Delhi: British Council; 1996;23–38
118. Singh GS, Prospects of indigenous medicinal plants of Himachal Himalaya. Indian Forester. 2004; 130(1): 62-70.
119. Singh GS. Ethnobiological and bio-medicinal wealth of western Himalaya, India. Journal of medicinal and Aromatic plant Sciences, 2004; 26:517–526.
120. Singh JK, Thakur KA. Medicinal Plants of the Shimla hills, Himachal Pradesh: A Survey. International Journal of Herbal Medicine, 2014;2(2):118-127.
121. Singh KH, Thakur AK. Medicinal Plants of the Shimla hills, Himachal Pradesh: A Survey. International Journal of Herbal Medicine. 2014; 2(2):118-127
122. Singh KK, Kumar K. Ethnobotanical Wisdom of Gaddi Tribe in Western Himalaya. Dehradun: Bishen Singh Mahendra Pal Singh Publishers. 2.000
123. Singh KN, Lal B. Ethnomedicines used against four common ailments by the tribal communities of Lahaul-Spiti in Western Himalaya. Journal of Ethnopharmacology, 2008; 115:147-159.
124. Singh SK, Rawat GS. Flora of Great Himalayan National Park; Himachal Pradesh. Dehradun: Bishen Singh Mahendra Pal Singh; 2000.
125. Singh SK. Ethnobotanical study of useful plants of Kullu district in north western Himalaya, India. Journal of Economic and Taxonomic Botany 1999; 23:185-198
126. Singh V, Chauhan NS. Traditional practices of herbal medicines in the Lahaul valleys, Himachal Himalayas. Indian Journal of Traditional Knowledge, 2005; 4(2):208-220.
127. Smith E, Smith H.B. Shimla Flowers: An Annotated List of Flowers Collected in the Neighbourhood of Simla and Mashobra. Privately Printed, Simla and Calcutta, 1899.
128. Sood SK, Nath R and Kalia DC. Ethno botany of cold desert tribes of Lahaul–Spiti (N. W. Himalaya). New Delhi: Deep Publications; 2001;161 p.
129. Srivastava TN, Kapahi BK, Sarin YK. Ethnobotanical studies in Lahaul and Spiti, Himachal Pradesh. Ancient Science of Life, 1992;119:126-130.
130. Stainton A. Flowers of the Himalayas: A Supplement. Oxford University Press, New Delhi, 1988.
131. Sultan J, Singh CJ. Herbal Market Spectrum. Sirmor, Himachal Pradesh: Grameen Vikas Evam Paryavaran Jagran Samiti, Paonta Sahib; 2006: 1(2):1–7 Ved Prakash. Indian Medicinal Plants; Current Status. In Samant SS, Dhar U, Palni, LMS (eds), Himalayan Medicinal Plants: Potential and Prospects. Nainital: Gyanodaya Prakashan; 2001; 45–65
132. Thakur A, Singh S, Puri S. Exploration of wild edible plants used as food by Gaddis-a tribal community of the Western Himalaya. Scientific World Journal, 2020, 6 p., Article ID 6280153.
133. Thakur AK. Common Medicinal Herbs of Shimla City, Himachal Pradesh, India. In Medicinal Plants: Distribution, Utilization and Significance. Discovery Publishing House Pvt. Ltd., New Delhi. 2015; 113-125.
134. Thakur AK. Study of Ethnobiological aspects of the Life of Nomadic and Pastoral Gujjars of Himachal Pradesh. A Project Report Submitted to University Grants Commissions, New Delhi, 2013.
135. Thakur K, Puri S. Ethnobotanical plants of bandli wildlife sanctuary, mandi, himachalpradesh. International Journal of Advanced Research 2016;4(6):106-108.
136. Thakur KS, Kumar M, Bawa R, Bussmann RW. Ethnobotanical study of herbaceous flora along an altitudinal gradient in Bharmour forest division. District Chamba of Himachal Pradesh, India: Evidence based Complementary and Alternative Medicine. 2014; https://doi.org/10.1155/2014/946870.
137. Thakur M, Asrani RK, Thakur S, Sharma PK, Patil RD, Lal B, et al. (). Observations on traditional usage of ethnomedicinal plants in humans and animals of Kangra and Chamba districts of Himachal Pradesh in North-Western Himalaya, India. Journal of Ethnopharmacology, 2016; 191:280–300.
138. Uniyal MR, Chauhan NS. Medicinal plants of Vhat valley in Kangra forest. Indian Journal of Medical Research, 1971;6:287–299.
139. Uniyal SK, Singh KN, Jamwal P, Lal B. Traditional use of medicinal plants among the tribal communities of Chhota Bhangal, Western Himalaya. J. Ethnobio. Ethnomed. 2006;2:2-14.
140. Upadhyay D. Ethno-botanical Important Plants in the parts of Shivalik Hills of Kangra district, Himachal Pradesh. Int. J. Sci. Res. Publ., 2013; 3(6):50.
141. Ved DK, Kinhal GA, Ravikumar K, Prabhakaran V, Ghate U, Vijaya Shankar R, Indresha JH. Conservation assessment and management prioritization for the Medicinal plants of Jammu & Kashmir, Himachal Pradesh & Uttaranchal. Bangalore, India: Foundation for Revitalisation of Local Health Traditions; 2003.
